# Genome-wide Identification, Expression Profiling and Evolutionary Analysis of Auxin Response Factor Gene Family in Potato (*Solanum tuberosum* Group Phureja)

**DOI:** 10.1038/s41598-018-37923-7

**Published:** 2019-02-11

**Authors:** Shuangwei Song, Liaoyang Hao, Pan Zhao, Ya Xu, Naiqin Zhong, Hongji Zhang, Ning Liu

**Affiliations:** 1 College of Plant Protection, Yunnan Agricultural University, Kunming, 650201 China; 2 State Key Laboratory of Plant Genomics, Institute of Microbiology, Chinese Academy of Sciences, Beijing, 100101 China; 3 School of Agriculture, Ningxia University, Yinchuan, 750021 China

## Abstract

Auxin response factors (ARFs) play central roles in conferring auxin-mediated responses through selection of target genes in plants. Despite their physiological importance, systematic analysis of *ARF* genes in potato have not been investigated yet. Our genome-wide analysis identified 20 *StARF* (*Solanum tuberosum* ARF) genes from potato and found that they are unevenly distributed in all the potato chromosomes except chromosome X. Sequence alignment and conserved motif analysis suggested the presence of all typical domains in all but StARF18c that lacks B3 DNA-binding domain. Phylogenetic analysis indicated that potato ARF could be clustered into 3 distinct subgroups, a result supported by exon-intron structure, consensus motifs, and domain architecture*. In silico* expression analysis and quantitative real-time PCR experiments revealed that several *StARF*s were expressed in tissue-specific, biotic/abiotic stress-responsive or hormone-inducible manners, which reflected their potential roles in plant growth, development or under various stress adaptions. Strikingly, most *StARF*s were identified as highly abiotic stress responsive, indicating that auxin signaling might be implicated in mediating environmental stress-adaptation responses. Taken together, this analysis provides molecular insights into *StARF* gene family, which paves the way to functional analysis of *StARF* members and will facilitate potato breeding programs.

## Introduction

The phytohormone auxin provoke almost every aspect of plant growth, development, stress responses, including apical dominance, defense responses against pathogens, leaf morphology, flower/fruit development, root elongation, vascular differentiation and embryogenesis^1–3^. Some phytopathogens, such as Agrobacterium, Pseudomonas, Streptomyces, and so on, can produce indole-3-acetic acid (IAA) and/or its analogs to modulate or manipulate plant responsive behaviors through auxin-induced signaling cascades and gene expressions^4,5^.

Considerable progress has been made over recent years in understanding how the cellular auxin response machinery was triggered in plants^6^. Genetic studies have revealed that, in the auxin signaling pathway, the core components are the F-box-containing TRANSPORT INHIBITOR RESPONSE 1 (TIR1) and its homologous AUXIN-SIGNALING F-BOX PROTEINS (AFBs) proteins, the transcriptional co-repressors AUXIN/INDOLE-3-ACETIC ACID (Aux/IAA), and the transcription factors AUXIN RESPONSE FACTOR (ARFs)^7–9^.In the absence of auxin, interaction between Aux/IAA and ARF proteins inhibited the transcription of auxin-responsive genes; upon the perception of auxin signals, TIR1/AFB protein forms SCF^TIR1/AFBs^ complex with AtCUL1, AtRbx1 and ASK1/2, and recruits Aux/IAA proteins, leading to their subsequent degradation via ubiquitin-proteasome-dependent pathway. The removal of Aux/IAA conduces to ARF-ARF dimerization or interaction with other transcriptional regulators, which determines the transcription of auxin-responsive genes^10^. As the last step in auxin signaling hierarchy prior to gene regulation, ARFs play central roles in conferring specificity to auxin response through selection of target genes.

The ARF proteins are a set of plant-specific transcription factors, whose typical architecture consists of a conserved amino-terminal DNA binding domain (DBD), a highly conserved carboxyl-terminal domain (CTD) and a variable middle region (MR)^11^. The DBD is composed of plant-specific B3-type motif, responsible for specifically binding to cis elements like AuxREs (TGTCTC) or its variant (TGTCCC or TGTCAC) in ARF-regulated genes, and an ancillary motif (Auxin_Resp) of unknown function^12^. The C-terminal Phox and Bem1 (PB1) domain, which were originally described as motif III and IV in Aux/IAA family of proteins, facilitates homo- and hetero-dimerization with Aux/IAA proteins as well as between ARF proteins^13^. The non-conserved MR is enriched by biased amino acid like glycine (Q), leucine (L), serine (S), and/or proline (P) residues, and the amino acid composition of MR sequence determines the transcriptional ability to activate or repress auxin-responsive genes^14,15^. To date, *ARF* gene family has been investigated in several plant species^16^, including *Arabidopsis thanliana*, *Oryza sativa*, *Solanum lycopersicum*, *Zea mays*, *Glycine max* and *Gossypium raimondii*^17–21^. The genome of model plant Arabidopsis contains 23 ARF genes, suggesting that ARFs are encoded by a moderate-sized gene family. Hereafter, 25, 51, 36, 21 ARF members were identified in rice, soybean, corn and tomato, respectively^17,19–22^. Given that multiple members in ARF family, there might be significant functional diversification between individual members.

Current knowledge of the biological roles of individual ARFs is largely from the characterization of model plants, Arabidopsis and rice. In Arabidopsis, ARF3 identified as a mediator of crosstalk between self-incompatibility (SI) signaling and pistil development, acts non-cell-autonomously to enhance the SI response and simultaneously attenuate auxin responses in stigma epidermal cells, probably by regulating a mobile signal derived from the stylar vasculature^23^; ARF5/MONOPTEROS directly binds to promoter region of *ATHB8*, a homeodomain-leucine zipper transcription factor gene involved in procambial cell fate acquisition, and is also required for the expression of the auxin efflux facilitator PIN1, further mediating organ and vascular tissue formation^24,25^; ARF6, redundantly with ARF8, dynamically regulates the flower maturation including stamen filament elongation, anther dehiscence, stigmatic papillae elongation, gynoecium maturation and flower bud opening^26,27^. In rice, down-regulation of *OsARF1* leads to growth retardation, short curled leaves and sterile phenotype in transgenic plants, suggesting its essential role in vegetative organs and seed development^28^; OsARF12 is regarded as one of major player in phosphate-induced auxin responses, indicating that ARFs might be involved in phosphate homeostasis in crops^29^.

Potato (*Solanum tuberosum*), the third most important global food crop, is regarded as the most widely grown non-cereal crop, and over 1 billion people have potato as daily diet^30^. *Solanum tuberosum* Group phureja DM1-3 516 R44 (hereafter referred to as DM) is a homozygous doubled-monopoloid potato, ~727 Mb genome of which was annotated at Potato Genome Sequencing Consortium (PGSC)^31^. Considering that ARFs are implicated in plant growth, development and stress adaptions, information on potato ARF gene family is needed for better understanding molecular mechanism between auxin signaling and physiological processes in this crop plants. Yet potato ARF gene family, to our knowledge, still remains unexplored. In this study, taking advantage of the DM potato reference genome, we conducted a genome-wide, comprehensive analysis of ARF family genes in potato. A total of 20 *StARF* (*Solanum tuberosum ARF*) genes was identified, and the physical and chemical characteristics, genomic structures, chromosomal locations, evolutionary relationship/scenario, expression profiles and protein-protein interaction network of StARF family were investigated in detail. These analyses will contribute to the better understanding the role of ARF genes in auxin-involved responses in potato and will also provide a robust database for the potato research community.

## Results

### Genome-wide identification of ARF genes from *Solanum tuberosum*

In order to identify ARF genes in potato, we first used combination of B3 DNA binding (Pfam 02362), Auxin_Resp (Pfam 06507) and PB1 (Pfam 02309) domains as the query to perform local BLAST searches against the potato genome database in Phytozome as well as Potato Genomics Resource. After removing the non-representative splicing forms of same gene locus, sequences of non-redundant ARF candidates were further verified by a Hidden Markov Model (HMM)-based for the presence of both B3 DNA binding and Auxin_Resp motifs, and a total of 20 ARF members were identified in the genome of *Solanum tuberosum*. The nomenclature for *StARF* members in the present study was given according to the homologies against Arabidopsis *ARF*s (Supplementary Fig. S1). Information on the gene name, loci number chromosomal location, predicted characteristics of putative proteins of individual *StARF* genes are listed (Table 1). Of these, the molecular weight of putative StARF proteins ranged from 40.1 to 130.1 kDa. It is noteworthy that the majority of *StARF*s encoded weakly acidic proteins as supported by the predicted isoelectric points. The results reinforced the assumption that these StARFs might participate diverse biochemical processes under disparate *in vivo* environments.

**Table 1:** List of putative ARF gene family members of *Solanum tuberosum* Group phureja.

Gene Name^a^	Locus ID^b^	Chromosomal Location^c^			Gene models^d^	Putative Proteins^e^		
		Chr	Chr_start	Chr_end		Length (aa)	pI	MW (kDa)
*SpARF1*	PGSC0003DMG400020711	01	79831315	79836550	4	654	6.34	72.73
*SpARF2a*	PGSC0003DMG400014179	03	57974781	57979999	2	845	6.57	93.93
*SpARF2b*	PGSC0003DMG400014452	12	48741766	48746463	2	829	6.67	92.63
*SpARF3*	PGSC0003DMG400031769	02	33155735	33161595	3	748	7.70	81.68
*SpARF4*	PGSC0003DMG400008065	11	41113192	41121535	2	811	6.04	90.32
*SpARF5*	PGSC0003DMG400003771	04	70600583	70605639	1	929	5.52	102.94
*SpARF6a*	PGSC0003DMG400015919	07	42117999	42125364	2	892	6.25	98.78
*SpARF6b*	PGSC0003DMG400028826	12	5941545	5948365	4	884	6.33	97.97
*SpARF8*	PGSC0003DMG401018664	03	2956825	2963831	5	752	6.30	84.28
*SpARF10a*	PGSC0003DMG400008081	11	41585187	41588350	1	699	7.96	77.26
*SpARF10b*	PGSC0003DMG400024320	06	55805274	55807974	1	676	5.86	75.42
*SpARF13*	PGSC0003DMG400023345	05	51547425	51552730	5	650	5.46	74.26
*SpARF16*	PGSC0003DMG400021560	09	5683172	5685932	4	696	6.91	76.42
*SpARF17*	PGSC0003DMG400027798	11	9698606	9700138	1	361	7.82	40.05
*SpARF18a*	PGSC0003DMG400000118	01	73618325	73621833	3	685	6.31	76.55
*SpARF18b*	PGSC0003DMG400012261	08	56233992	56237935	2	655	6.68	73.49
*SpARF18c*	PGSC0003DMG400005794	08	5265463	5267318	1	382	4.81	42.29
*SpARF19a*	PGSC0003DMG400002392	07	40015771	40022951	2	1157	6.42	130.15
*SpARF19b*	PGSC0003DMG400009773	07	9124673	9131581	1	1114	6.38	123.66
*SpARF19c*	PGSC0003DMG400013686	05	43161023	43166783	1	1097	6.30	121.26

^a^Name referred to systematic designation to *S. tuberosum* ARFs in this work.

^b^Gene accession number in PGSC database.

^c^Chromosomal location of the *StARF* genes in the DM1-3 potato genome (V4.3).

^d^Isomer numbers.

^e^Length (number of amino acids), molecular weight(kilodaltons), and isoelectric point (pI) of the deduced polypeptides were calculated using Lasergene Molecular Biology Suite (Version 7.0).

### Chromosomal distribution of *StARF* genes

**Figure 1:**
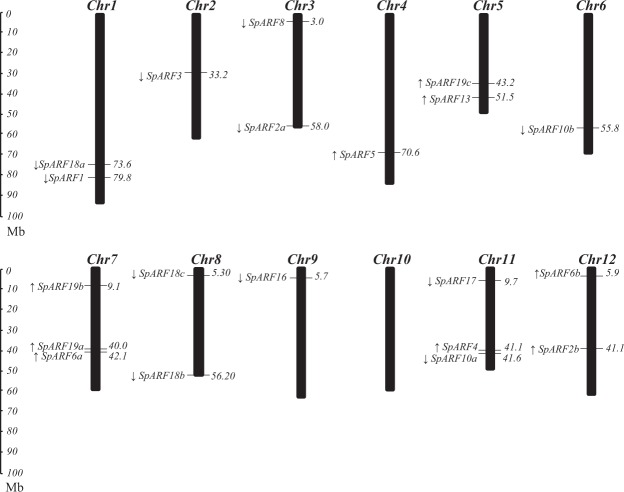
Genomic distribution of *StARF* genes on DM1-3 chromosomes. The chromosome numbers and size are indicated at the top and bottom of each bar, respectively. The arrows next to gene names show the transcription directions. The number on the right side of the bars designated the approximate physical position of the first exon of corresponding *StARF* genes on potato genome.

The chromosomal location and direction of transcription for each *ARF* gene were established in DM potato. As shown in Fig. 1, 20 *StARF* genes were positioned on all chromosomes except the chromosome X, however the number of *StARF* genes were unevenly distributed to potato chromosomes. Three each *StARF*s were present in chromosome VII and XI; two each were on chromosome I, III, V, VIII and XII; one each located on chromosome II, IV, VI and IX.

Gene duplication is one of the most important mechanisms for acquiring new genes and creating genetic novelty in organisms. Although the member of *ARF* genes is similar between potato and Arabidopsis, five possible duplication events were observed as sequence alignment suggested that some *StARF* genes exhibited extreme-high sequence similarity. Among them, *StARF2a-2b*, *StARF6a-6b* and *StARF10a-10b* form twin pairs, *StARF18a/b/c* and *StARF19a/b/c* from triplet pairs. Surprisingly, no tandem duplication events were identified because most duplicated *StARF* genes were positioned to different chromosomes, or they were separated by at least several megabases.

### Conserved motif and domain analysis of StARFs

**Figure 2:**
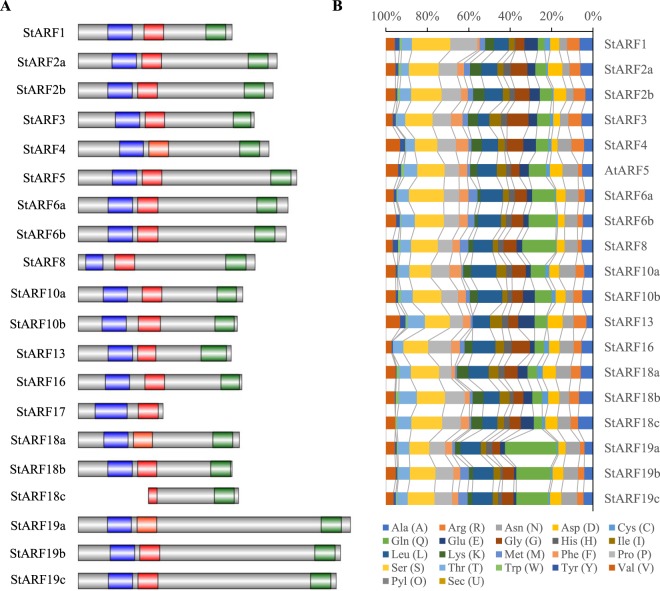
Analysis of conserved domains in StARF porteins. (**A**) Schematic organization of conserved domains in StARF proteins. The B3 DNA-binding domain, Aux_Resp domain, and PB1 domain are shown in blue, red and green, respectively. (**B**) Amino acid composition of MR domains in StARF proteins. Bars represent the percentage of different amino acid residues in MR domains of StARFs, and each color represents one kind of amino acid.

It is reported that most ARF proteins carry a conserved N-terminal DNA-binding domain composed of a plant specific B3-type and Auxin_Resp motifs, and a highly conserved C-termianl PB1 domain corresponding to motif III and IV of the Aux/IAA proteins^32,33^. To better understand the structural similarity of these ARFs, a multiple sequence alignment was conducted with the deduced amino acid sequences of *StARF* genes (Supplementary Fig. S2). The results revealed that all StARF proteins contained highly conserved and intact DBD domains, except that StARF18c had a truncated DBD domain (Fig. 2). Although the partial sequence of Auxin_Resp motif was retained, it is plausible that the DNA-binding ability of StARF18c could be impaired due to the lack of DNA-binding domain.

Unlike conserved DBD and CTD regions, the protein sequence of the Middle Regions (MRs) are high variable. However, the amino acid composition of the MRs exhibits some non-conserved, unique features between ARF proteins^32^. It has been proposed that ARF proteins whose middle regions are rich in S, P, L and G might act as transcriptional repressors, whereas those rich in Q, Sand L in MR might be activators^34,35^. Composition analysis of StARF proteins suggested that the MRs of StARF6a/b, StARF8, StARF19a/b/c are abundant in Q residues, suggesting they might be transcriptional activators (Fig. 2B). Surprisingly, it seems that the MR of StARF17 was completely missing, which made it impossible to speculate its transcriptional activity (Fig. 2).

### Structural analysis of *StARF* genes

**Figure 3:**
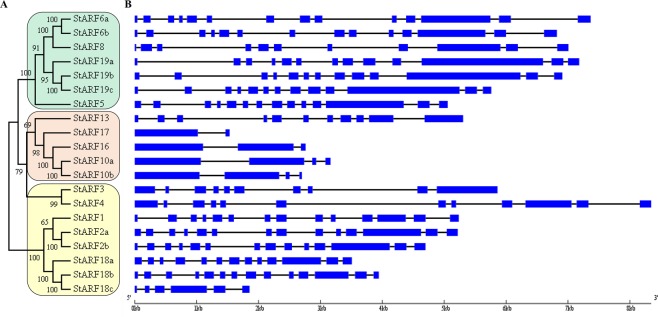
Classification of *S. tuberosome* ARF proteins. (**A**) Neighbor-joining tree were generated using MEGA 7.0 to determine the phylogenetic relationship between *S. tuberosome* ARF proteins (left). According to classification proposed by Finet *et al*. (2013), StARFs were divided into three subgroups: (**A**–**C**) Shadow colors were used to distinguish different StARF subgroups. (**B**) The intron-exon organization of *StARF* genes was plotted using Gene Structure Display Server (Version 2.0). Blue boxes represent exons, and grey lines represent introns.

In addition to conserved protein domains, the pattern of intron positions between ARF subgroups can also provide some clues on evolutionary relationships. To identify the introns-exons structure of individual StARF member, a comparison of the full-length cDNA sequences with the corresponding genomic DNA sequences was performed. Accordingly, we constructed a neighbor-joining phylogenetic tree using the amino acids sequences of 20 StARF members, resulting in 3 major ARF clades with very high bootstrap confidence values (Fig. 3A). In the members of *StARF5/6/8/19* clade, there are 10–13 introns, and members of *StARF1/2/3/4/18* clade have 9–13 introns except that truncated *StARF18c* contains 5 introns (Fig. 3B). Consistent with previous findings from Arabidopsis and rice, members of *StARF10/16/17* clade have relatively lower number of introns (Fig. 3B). Strikingly, the exon-intron structure is conserved within subclades including *StARF6a/b*, *StARF19a/b/c*, *StARF10a/b*, *StARF2a/b* and *StARF18a/b*, although the sizes of introns vary greatly for different members; however, the exon-intron structure varies between subclades, which was also supported by the phylogenetic analysis of StARF proteins.

### Phylogenetic analysis of StARFs

**Figure 4:**
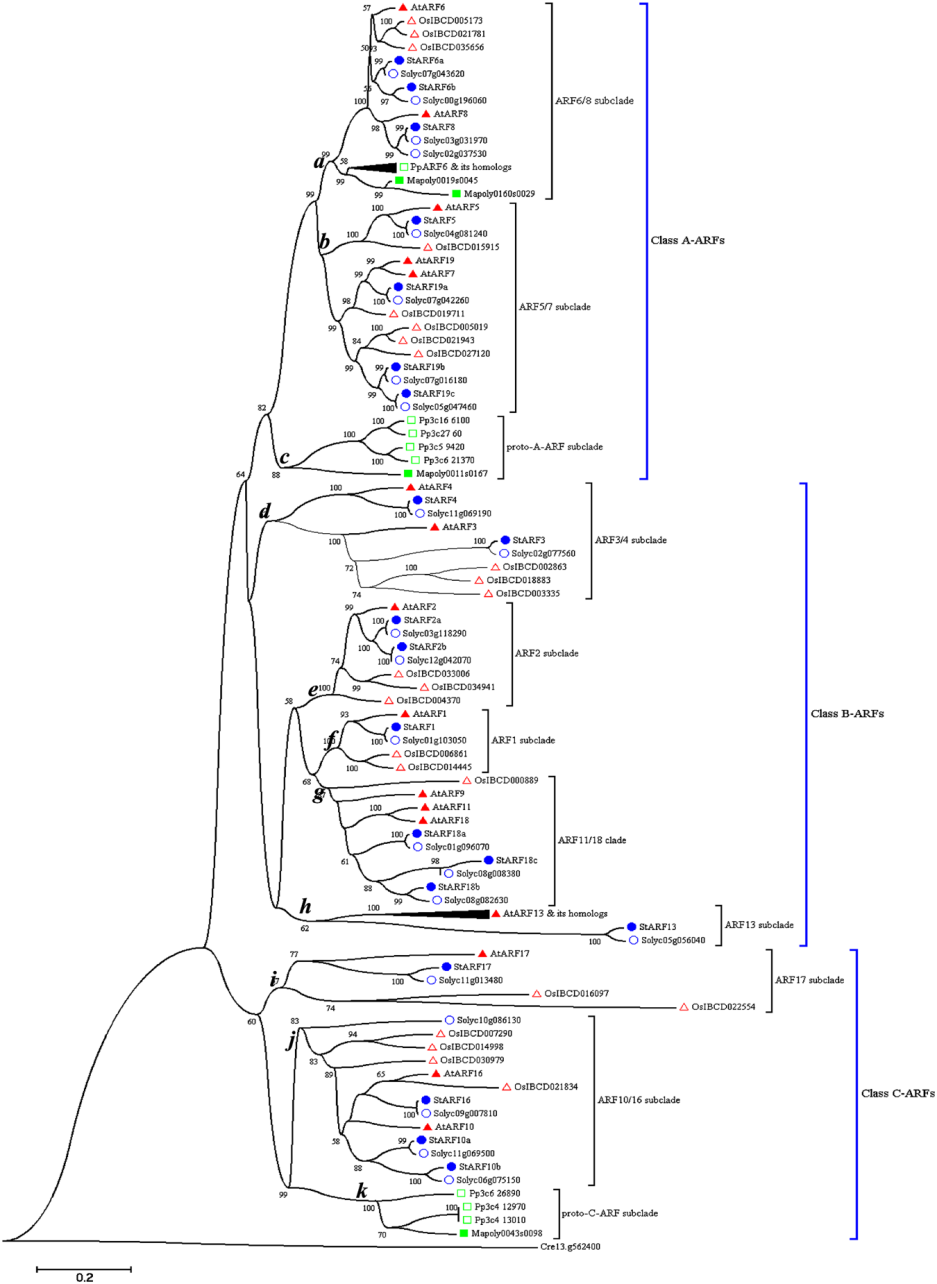
Phylogenetic analysis of ARF proteins in liverwort, the moss, and flowering plants. Neighbor-joining tree was constructed based on the alignment of ARF protein sequences from *Marchantia polymorpha*, *Physcomitrella patens*, *Oryza sativa*, *S. lycoperiscum*, *S. tubersomem* and *Arabidopsis thaliana*. The position of the root was determined from an outgroup consisting of an ARF-like sequence from green algae *Chlamydomonas reinhardtii*. The percent bootstrap support for 500 replicates is shown on each branch with >50% support. Some nodes were designated with letters in bold and italic. Sequences used in phylogenetic analysis were provided in Supplementary Data.

To gain the insight into the evolutionary scenario of StARFs and their homologs in other plant species, we expanded the neighbor-joining analysis to include ARF sequences from other taxa: 4 ARF sequences from the liverwort *Marchantia polymorpha*, 15 sequences from the moss *Physcomitrella patens*, 24 sequences from tomato, 23 sequences from rice, and 23 from Arabidopsis (Fig. 4). A B3 domain-containing sequence from green algae *Chlamydomonas reinhardtii* was used as outgroup. In this analysis, 111 of ARF sequences were joined in a moderately well-supported phylogenetic tree. Cre13.g562400 from *C. reinhardtii* is the most divergent gene in the overall organization as it showed sequence similarity to ARF genes in B3 DNA-binding region and lacks other motifs. Consistent with analyses by Mutte *et al*. (2018) and Finet *et al*. (2013), the ARFs can be classified into 3 major clades on their phylogenetic relationship. Clade A had 49 members, which were orthologs of AtARF5, AtARF7, AtARF6/8 and AtARF13; Clade B included 33 members, and they were homologous to AtARF1, AtARF2, AtARF3 and AtARF4; the remaining ARFs belonged to Clade C, which consisted of AtARF17 and other 20 homologs.

We further analyzed the phylogeny of the ARF family in more detail (Fig. 4). Clade A contained 3 subclades, which were represented by nodes *a*, *b* and *c* in Fig. 3, and the relationships of subclades in clade A to one another were well supported by bootstrap percentage. The node *a* (ARF6/8 subclade) subclade was placed as twin-sister to the node *b* (ARF5/7 subclade), while node *c* subclade, containing class A-ARFs of bryophytes, was basal to other two subclades, suggesting they might represent precursors of class A-ARFs of land plants. Clade B could be further divided into 5 subclades, represented by node *d* (ARF3/4 subclade), *e* (ARF2 subclade), *f* (ARF1 subclade), *g* (ARF11/18 subclade) and *h* (ARF13 subclade). Strikingly, clade B consisted of ARF members only from land plant species, indicating that duplication and diversification class B-ARFs might occur in euphyllophytes. Clade C was composed of node *i* (ARF17 subclade), *j* (ARF10/16 subclade) and *k* (bryophyte C-ARFs). As observed in clade A, we found that node *k* subclade was only represented by members from bryophytes, implying they might be a group of proto-C-ARFs.

### Expression profiles of *StARF*s among various tissues and developmental stages

**Figure 5:**
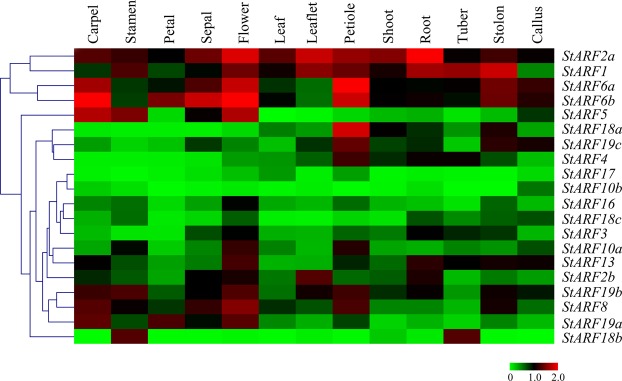
Expression profiles of *StARF* genes with hierarchical clustering in different tissues. The Illumina RNA-Seq data were obtained from PGSC database, and the FPKM value of representative transcripts of *StARF* genes were log_2_ transformed for further analysis. The normalized expression data was used to generate heatmap with hierarchical clustering based on the Manhattan correlation with average linkage using MeV software package. Color scale below heatmap shows the expression level; red indicates high transcript abundance while green indicates low abundance.

To understand the tissue-specific expression profile of *StARF* genes, FPKM (Fragments per Kilobase of transcript per Million mapped reads) values generated by PGSC were used to assess their expression levels across different organs and developmental stages. The 13 RNA-seq database could be divided into 4 major groups: floral (carpel, petals, sepals, stamens and mature flower), leaf (whole leaf, leaflet and petiole), tuber (tuber, stolon), and others (shoot, root and callus)^36^. As shown in Fig. 5, of these 20 *StARF* genes, *StARF1/2a/6* were ubiquitously abundant in most tissues, suggesting that they might execute some universal roles in auxin-regulated plant growth and development processes. On the contrary, we also found that expression of several *StARF*s, including StARF3/10b/16/17/18a, were relatively lower.

With respect to floral tissues, transcripts of *StARF2/5/6/8/19b* were abundant in carpels, *StARF1/2a/5/6/18b/19b* were preferentially expressed in stamens, *StARF6b/19a* were stamen-specific ARFs, and *StARF2a/6/8* is highly expressed in sepal tissues. The different expression domains of *StARF*s implied that they might coordinate the expression of target genes involved in the flower development. In addition to *StARF1/2a*, *StARF4/6/8* were highly expressed in leaf or petiole tissues, whereas *StARF2b/13* were abundant in root tissues, implying they might play roles in auxin-involved organ development.

Potato tubers develop from underground stolons, and in an *in vitro* tuberization system, exogenously applied auxin can stimulate the tuberization process. Therefore, ARFs could play a role in tuber growth. We found that a relative high level of expression of *StARF1/2a/6* was observed in stolons, which might contribute to the swelling of stolons. However, *StARF1/2a/18b* expression was much higher than other *StARF*s, suggesting they might function in root growth.

### Differential expression of *StARF*s during biotic and abiotic stresses

**Figure 6:**
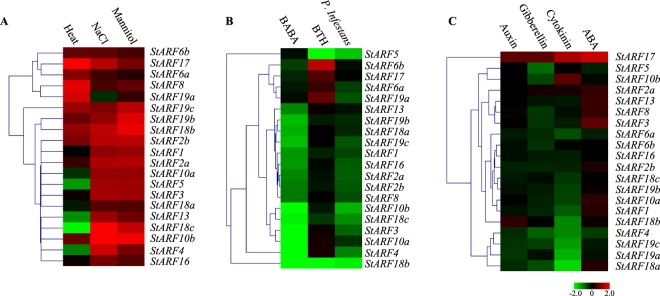
Heatmap representation and hierarchical clustering of *StARF* genes under abiotic stresses (**A**), biotic stresses (**B**), and phytohormone treatments (**C**). The Illumina RNA-Seq data were obtained from PGSC database, and the relative expression of *StARF* genes was calculated with respect to control samples using FPKM values of representative transcripts corresponding to *StARF* genes. Fold changes of *StARF* expression were log_2_ transformed, and the normalized expression data was used to generate heatmap with MeV software package using the same parameters in Fig. 4. Color scale below heatmap shows the expression level; red indicates high transcript abundance while green indicates low abundance.

Though auxin was generally regarded as one of major growth-promoting hormones, accumulated evidence highlighted its role in mediating stress responses through cross-talking with other signaling pathways^37,38^. ARF transcription factors might be one of potential targets in face of abiotic/biotic stresses and hormones. Therefore, the expression patterns of *StARF* genes under various stress, hormone (and their analog) treatments were investigated by analyzing the log2 fold change between treatments and controls. All 20 *StARF*s, with the exception of *StARF19a*, showed strong up-regulation in response to salt stress and mannitol treatment, whereas only half were highly expressed upon heat stress (Fig. 6A). We also found that expression of *StARF5/10a/10b/13/18a/18c* was decreased in response to thermal conditions (Fig. 6A). Thus these results implied that responsive behavior of *StARF* genes were associated with plant adaption to diverse environmental stimuli.

The oomycetes *P. infestans* infection, as well as BABA treatment, resulted in the inhibition of most *StARF*s (Fig. 6B), clearly suggesting that a majority of ARF-regulated genes were suppressed, and thus auxin could promote disease susceptibility. However, when plants treated with benzothiadiazole S-methyl ester (BTH), an SA analog, only *StARF6/17/19a* showed strong up-regulation, while *StARF5/18b* went in the opposite way. Likewise, expression of other *StARF* members was slightly changed, implying that BTH could induce disease resistance via a molecular mechanism that differs from BABA.

Similarly, we also examined the expression responses of *StARF*s under various phytohormone treatments (Fig. 6C). Surprisingly, only *StARF17/18b* were induced by auxin treatment, whereas expression level of other *StARF* members was slightly decreased or even unchanged. Despite that *StARF17/2a* expression was induced by GA3, most *StARF* members slightly decrease their expression in response to gibberellin. In presence of the 6-Benzylaminopurine (BAP), expression of *StARF1/4/6a/18a/18b/19a/19c* was attenuated, while expression of *StARF10b/17* was induced by cytokinin applications. Intriguingly, abscisic acid (ABA), instead of auxin, could induce the expression of nearly half of *StARF* members like *StARF3/13/17*, while the induction was not observed in other members. Therefore, these results indicated that some StARF transcription factors, possibly through regulating their target genes, participated diverse abiotic signaling pathways, and were major players in environmental stresses.

### Validation of *StARF*s expression patterns

**Figure 7:**
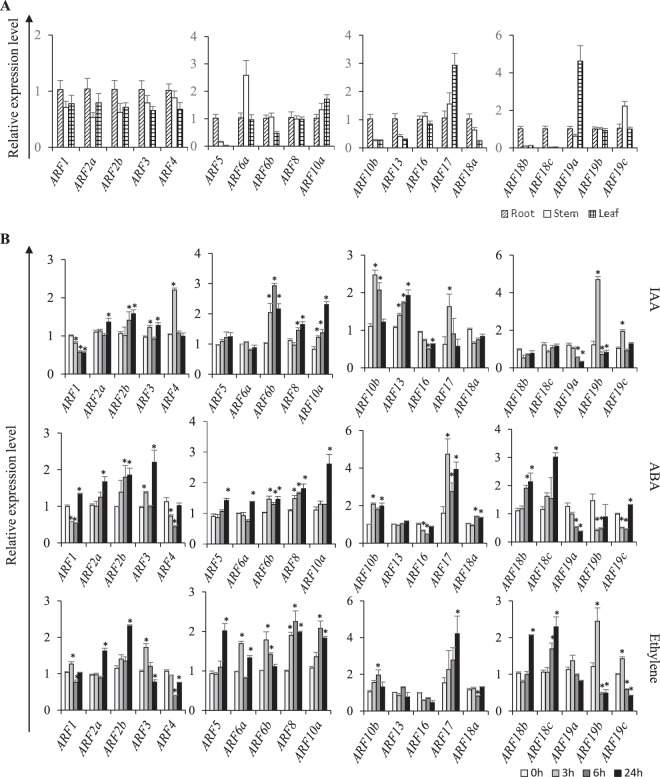
qRT-PCR analysis of *StARF* genes in different tissues (**A**) and phytohormones (**B**) StARF transcript levels measured by real-time qRT-PCR from the various tissues or under phytohormone treatments at indicated time points. Data are means of three biological replicates (8 pooled plants each), and error bars denote SE. Potato elongation factor *StEF-1α* gene was used as an internal control. Stars above the error bars indicate significant differences between treatments and controls (according to student’s t-test). qRT-PCR primers for *StARF* and *StEF-1α *genes were provided in Supplementary Table S1.

Although Illumina RNA-seq data provides preliminary information on the expression behavior of *StARF* genes, in some instances, there are some discrepancies between *in silico* analysis and experimental data. Thus, we carried out quantitative real-time RT-PCR analysis of *StARF* genes in several tissues or under several representative conditions, and potato cultivar Shepody was used for the validation experiments. The qRT-PCR results of *StARF* genes in root, stem and leaf tissues were largely in good agreement with RNA-seq data (Fig. 7A). However, we also found that some members of *StARF* gene family showed different expression patterns. For example, expression level of *StARF4* was similar between root and leaf tissues, which was contradicted the RNA-seq analysis. The inconsistency was due to the differences in collecting potato tissue samples, or probably was the consequence of genotype-dependent expressions.

In another experiment, qRT-PCR experiments were conducted on selected conditions to validate the expression patterns of *StARF*s in response to IAA and ABA treatments (Fig. 7B). By and large, expression patterns of *StARF*s under selected conditions were similar to RNA-seq results. For example, we observed that *StARF17* was strongly induced upon IAA and ABA treatments, and expression of *StARF3* was induced by ABA, but kept unchanged to auxin treatment, which is consistent with expression patterns revealed by RNA-seq analysis. Furthermore, we also investigated the expression patterns of *StARF*s under ethylene (ETH) treatment (Fig. 7B), as it is implicated in regulating plant growth, defense response and senescence. A noteworthy observation in this analysis most members of *StARF* gene family exhibited up-regulation in response to ETH treatment; contrary to this, we also found several *StARF* members, including *StARF4*, *StARF10b*, *StARF16*, and *StARF18a*, showed decreased expression or were unable to respond. The varying responses of *StARF* genes to ETH treatment could thus be attributed to their diverse roles in complex regulatory cross-talk between auxin and ethylene signaling.

### Interaction network of StARFs

**Figure 8:**
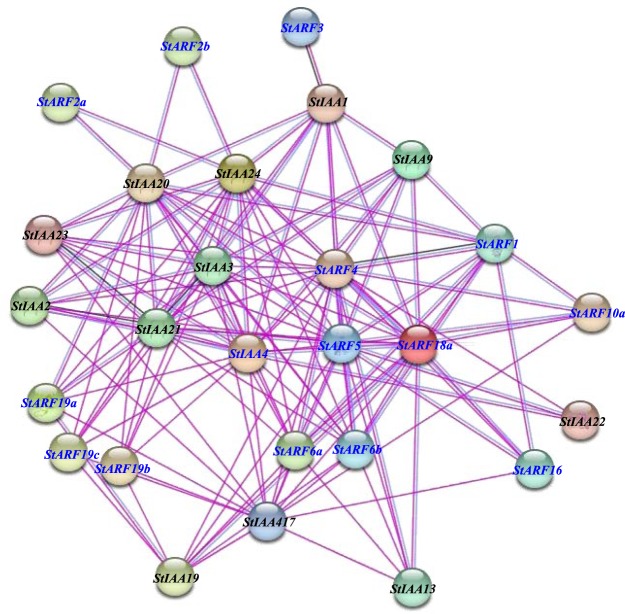
Predicted protein-protein interaction network of StARFs and StIAAs. Each node represents either one StARF protein (blue label) or StIAA (black label) protein. The disconnected nodes in the network were hidden due to the lack of supporting information. Edges between nodes represent protein-protein associations predicted by experimentally determined (pink line) or from curated databases (blue line).

Both ARF and AUX/IAA proteins contain PB1 domain, which contributes to the interaction of ARF-ARF, ARF-IAA, and IAA-IAA. To better understand gene functions, the STRING database was employed to generate the protein-protein network between ARF and AUX/IAA protein families in potato^39^. As shown in Fig. 8, large protein-protein interaction pairs were predicted between StARFs and StIAAs, indicating that members from the two protein families could homo- or hetero-dimerize to mediate ARF-regulated gene expression. Moreover, several StARFs were found to be core nodes in the whole network, indicating that they might be involved in different physiological functions by working with other PB1-domain containing proteins. For example, StARF4 was speculated to be associated with StIAA1/2/9/13/19/20/22/23 and StARF1/6/8/10a/16. On the other hand, a few StARFs could only interact with specific StIAAs, like StARF3-StIAA1, StARF2a-StIAA20/24, and StARF2b-StIAA20/24 pairs. Thus, these results illustrated how members of StARF and StIAA protein families might form functional transcription factor complexes, mediating the expression of most auxin-responsive genes in potato.

## Discussion

Auxin response factors are key players in auxin signaling cascade, which controls many physiological and development process in plants^2,40^. ARFs exert their roles by mediating the expression of downstream genes, thus regulating the cellular responses to plant hormone IAA or its analogs^41^. Previously, the ARF gene family has been intensively studied in model plant Arabidopsis^18^, and also to varying degree in a number of other plant species, such as rice, tomato, and soybean^17,21,22^. In our analysis, total 20 *StARF*s were identified from potato genome. Given that the genome size of DM1-3 potato was 740 Mb, nearly 5 times larger than Arabidopsis genome^31^, it is surprising that the number of *StARF* genes was much less than that of Arabidopsis. Apparently, our observations on *ARF* gene family contradicted with genome complexity between Arabidopsis and potato. With respect each clade A- and C-ARFs, there were 7 and 4 members of in potato respectively, whereas only 5 and 3 Arabidopsis counterparts were identified. Clade B-ARFs contained 9 members in potato, and 14 members in Arabidopsis, which leads to the big difference in the overall number of ARF genes between potato and Arabidopsis. One possible explanation for the paradox is that several independent, small-scale, segmental duplication events and chromosome rearrangements occurred at *ARF13* loci resulted in multiple members of *AtARF13* subclade, finally leading to the expansion of ARF gene family in Arabidopsis.

To assess the evolution trajectory of ARF genes, we also searched the available genome data of other plant lineages including green algae, bryophytes (liverwort and moss) and flowering plants, and nearly one hundred of ARF candidate sequences were obtained with similar blast method previously mentioned. Despite the occurrence of several genes encoding B3, III or IV motifs, Auxin_Resp domain was unable to be identified in algae genome. Nevertheless, Auxin_Resp and other three domains (B3, III and IV) in a single protein could be recognized from liverwort and moss genomes, thus reinforcing the idea that canonical ARF proteins were probably established during the evolutionary transition from algae to bryophyte plants.

Furthermore, we conducted a comprehensive phylogenetic approach using the conserved domains on our 111-ARF sequences from algae to flowering plants. Consistent with previous reports, ARF genes were classified into three main clades: clade A (ARF5/6/7/8), clade B (ARF 1/2/3/4/9) and clade C (ARF10/16/17). Among the three main ARF clades, we found that only clade B consisted of ARF sequences only from angiosperms, strongly suggesting the clade might be evolved from flowering plant species. Conversely, bryophyte ARF sequences were present in clade A and C, indicating that the ARFs had diverged into at least two group of transcriptional factors in the common ancestor of bryophyte, and this ancient event possibly predated the divergence of liverwort and moss. Strikingly, we detected that 5 and 4 ARF protein sequences from bryophyte, in our phylogenetic analysis, were loosely associated clade A and C, respectively, and these ARF sequences formed 3 independent clusters on basal of each subclade. As determined by morphology data, fossil evidence and molecular phylogenetic analyses, bryophytes diverged earlier that other land plants^42–44^. With these observations, it is likely that these bryophyte ARFs might resemble the counterparts of ancestral ARF genes in land plants.

Accumulating evidence suggested a link between plant growth hormone auxin and disease resistance^45,46^, while the behavior of auxin in pathogenesis is complicated. Most studies indicated that auxin promote disease symptoms in many plants. Exogenous IAA applications aggravated the disease progression during the infection of rice with *Xanthomona oryzae*^47^. Similarly, IAA application attenuated oligogalacturonide-induced resistance to *Botrytis cinerea* in Arabidopsis^48^. However, there were also some findings that auxin played positive roles of in disease resistance. For example, a metabolic profiling approach revealed that endogenous auxin level substantially increased during pathogen infection of Arabidopsis with *Pseudomonas syringae* DC3000 in comparison with mock plants^49^. *In silico* analysis also suggested that, in tomato plants, most ARF genes were differentially expressed in response to various pathogen infections of bacteria and virus^50^. Consistent with its complicated behavior of auxin, our findings revealed that expression patterns of *StARF* genes were varying when potato challenged by pathogen *P. infestans* or treated with disease resistance-priming agent BABA and BTH. Thus, we conclude that the different expression patterns of StARFs might reflect their complicated roles in plant immune response.

In our analysis, we found that expression of most *StARF* genes was closely associated with heat, salt and osmotic stresses, and expression of *StARF3/13/17* were also substantially induced by abscisic acid known as the abiotic stress hormone. Similarly, a whole genome microarray indicated that, among the 186 transcription factor genes, nine *OsARFs* were identified as drought- or salinity-responsive genes^51^. In soybean (*Glycine max*), expression of *GmARF33* and *GmARF50* was induced by water deficit. In sorghum (*Sorghum bicolor*) roots, *SbARF10*, *SbARF16* and *SbARF21* were induced by salinity, whereas expression of others was repressed^29^. Interestingly, *SbARF* genes were strongly induced by salt stress in sorghum leaf tissues, which was corroborated by the evidence that most *StARF* genes were induced upon abiotic stresses^29^. Additionally, Arabidopsis *ARF19* was proved to be an important player linking hormonal crosstalk of auxin and ethylene-mediated responses^52^. Our qRT-PCR experiments on *StARF* genes also revealed that in addition to *StARF19b*, there were a few *StARF* genes whose expressions were activated by ethylene treatment, and thus it is likely ARFs might be interaction nodes between auxin and ethylene signaling. Therefore, these observations strongly suggested that ARFs could be a class of potential key players, regulating spatio-temporal expression of stress-responsive genes to mitigate the negative effects of environmental or biotic stresses.

Auxin response factors is implicated in a plethora of developmental processes and stress-adaption responses. In this study, genome mining revealed the existence of 20 *StARF* genes from potato. Chromosomal distribution, conserved domains/motifs, and structural analysis phylogenetic studies have provided valuable insights into the evolution aspects of *StARF* gene family. Expression profiling of *StARF*s in different tissues gave us a cue on how these ARF transcriptional regulators participate auxin-signaling cascade in plant development. Moreover, the diverse responsive behaviors of *StARF*s in response to stresses, phytohormones and chemical inducers suggested that they were actively involved in the regulation of stress responses to biotic and abiotic stimulus. Protein-protein interaction analysis illustrated the translational regulation relationships of StARFs and its interacting proteins. In summary, our results provide new insights of *StARF* gene family, which will facilitate future selection and breeding for stress-tolerant potato cultivar.

## Materials and Methods

### Identification of ARF genes in *Solanum tuberosum* Group phureja

Phytozome (https://phytozome.jgi.doe.gov/), Potato Genome Sequencing Consortium (PGSC, http://solanaceae.plantbiology.msu.edu/), and NCBI (http://blast.ncbi.nlm.nih.gov/) online resources were searched to identify the entire family of ARF in *Solanum tuberosum* Group phureja DM1-3. The hidden Markov model (HMM) profiles of the ARF gene family which includes B3 DNA-binding domain (Pfam 02362), Auxin_Resp (Pfam 06507) and AUX/IAA family (Pfam 02309) were used to identify ARF candidates from potato genome. The potato ARF protein sequences were further confirmed using SMART (http://smart.embl-heidelberg.de/smart/batch.pl) and Pfam (http://pfam.xfam.org/search) to confirm the presence of conserved motifs mentioned above. In order to obtain non-redundancy ARF sequences, potato ARF sequences were further used as queries to blast against Phytozome database, and any redundancy was manually removed.

### Analysis of gene structure and conserved domains

Based on the genome annotation of DM assembly available in Phytozome, the intron-exon structure of individual *StARF* genes was predicated, and its genomic organization was visualized using Gene Structure Display Server 2.0 (GSDS, http://gsds.cbi.pku.edu.cn/)^53^.

Conserved domains in protein sequences were verified using ScanProsite (http://pro-site.expasy.org/scanprosite/) which provides information about positions of different domains in the protein sequence. This information was used to draw visual representation of distribution of domains in the deduced amino acid sequences of proteins using Illustrator for Biological Sequences (Version 1.0)^54^.

### Sequence alignment and phylogenetic construction

Phylogenetic analysis of ARF protein sequences used only the conserved N-terminal DNA-binding domain and the conserved C-terminal region corresponding to the Aux/IAA motif III-IV region. Neighbor-joining analyses of the Arabidopsis Aux/IAA and ARF sequences were conducted in PHYLIP 3.5 (http://evolution.genetics.washington.edu/phy-lip.html) using the PAM matrix of Dayhoff, with 500 bootstrap replicates and randomized sequence input order. One B3 domain-containing sequence from *Chlamydomonas reinhardtii* was used as outgroup.

### Expression profiling of ARF genes in different tissues or under various stresses

Transcriptome analyses were performed using the RNA-seq sequence information generated by the PGSC^36^. For developmental stages, the corresponding FPKM (fragments per kilobase per million reads) values for *StARF* genes were obtained for 13 tissues representing major organs and developmental stages, including floral (carpel, petals, sepals, stamens and mature flower), leaf (whole leaf, leaflet and petiole), tuber (tuber, stolon), and other organs (shoot, root and callus).

Abiotic stress-treated tissues included 24-h *in vitro* grown whole plants exposed to heat (35°C), NaCl (150 mM) and Mannitol (260 mM). Hormone treatments of *in vitro* grown whole plants consisted of abscisic acid (ABA, 50 mM), indole-3-acetid acid, (IAA, 10 mM), gibberellic acid (GA3, 50 mM), and 6-benzylaminopurine (BAP, 10 mM). Biotic stress were represented by detached leaves challenged by *Phytophthora infestans*, BABA (DL-β-amino-n-butyric acid), BTH (6-benzylaminopurine), and samples were pooled at 24, 48 and 72 h. Control plants were grown in parallel with each stress or hormone treatment. Similarly, FPKM values for abiotic or biotic stress-treated potato plants were analyzed by calculating the fold change of expression levels between treatments and the corresponding controls.

The normalized expression data was used to generate heatmap using the Institute for Genomic Research MeV software package (http://mev.tm4.org), and hierarchical clustering analysis (HCA) was built on the basis of the Manhattan correlation with average linkage method.

### RNA extraction and quantitative real-time RT-PCR

*Solanum tuberosum* (cultivar “Shepody”) plants were *in vitro* micropropagated on Murashige and Skoog (MS) medium plus 30 gL^−1^ sucrose and 0.8% agar (Sigma-Aldrich), with pH adjusted to 5.8. Potato seedlings were routinely subcultured as two-node segments every 3–4 weeks and incubated at 23°C with 16 h photoperiod under cool with fluorescent lamps (~70 μmol m^−2^ s^−1^ photon flux density). 3-week old plants were subjected to IAA (50 μM), ABA(100 μM) or ethylene (1 mM) treatments. The plant tissues were collected at designated points and immediately frozen in liquid nitrogen. Sample collections were performed on separate days for the replicates. Total RNA was extracted with Trizol (Invitrogen Inc., USA) as described by the manufacturer. RNA quantity and quality were assessed using a NanoDrop8000 (Thermo Scientific™, USA). Total RNA isolation and reverse transcription with oligo(dT)_18_ (18418-012; Invitrogen, USA) were performed as described previously^26^. The amounts of individual genes were measured with gene-specific primers by real-time PCR analysis with a cycler IQ real-time PCR instrument CFX96 (Bio-Rad, USA) and SYBR Green mixture (Bio-Rad, USA). The relative expression of specific genes was quantitated with the 2^−ΔΔ^Ct calculation method^55^, where ΔΔCt is the difference in the threshold cycles and the reference gene, which was potato *eIF-1α* for expression analyses. The sequences of specific primers are shown in Supplementary Table S1.

### Construction of interaction network of StARFs

Protein–protein interaction (PPI) analyses of StARF family were performed on the STRING website (http://www.string-db.org) to predict protein interactions^39^. Briefly, StIAAs previously identified^56^ were included as potential interacting-partners of StARFs. Amino acid sequences of the two families were used as query sequences with Arabidopsis as the model. In confidence mode, the online program was run with parameters as follows: the box “active interaction sources” was chosen to “Experiments”; the box “minimum required interaction score” was set to 0.9 (highest confidence); other boxes used default settings.

## Supplementary information

Supplmentary data
