# Supplementary material for: Genome-wide Identification, Expression Profiling and Evolutionary Analysis of Auxin Response Factor Gene Family in Potato (Solanum tuberosum Group Phureja)
Source: Sci Rep. 2019 Feb 11;9:1755. doi: 10.1038/s41598-018-37923-7 (PMC6370904; doi:10.1038/s41598-018-37923-7)
Supplement: Supplementary file 1 — Supplmentary data [file 41598_2018_37923_MOESM1_ESM.pdf]

Genome-wide Identification, Expression Profiling and Evolutionary Analysis of Auxin Response Factor Gene Family in Potato (*Solanum tuberosum* Group Phureja)

Shuangwei Song[1, 2 †], Liaoyang Hao[2 †], Pan Zhao[2], Ya Xu[2], Naiqin Zhong[2, 3], Hongji Zhang[1*], Ning Liu[2*]

[1] College of Plant Protection, Yunnan Agricultural University, Kunming 650201, China

[2] State Key Laboratory of Plant Genomics, Institute of Microbiology, Chinese Academy of Sciences, Beijing 100101, China

[3] School of Agriculture, Ningxia University, Yinchuan 750021, China

* To whom correspondence should be addressed: Tel. +86-10-6486 1547

Email: liuning@im.ac.cn or zhanghongji111@163.com

† These authors contributed equally to this work.

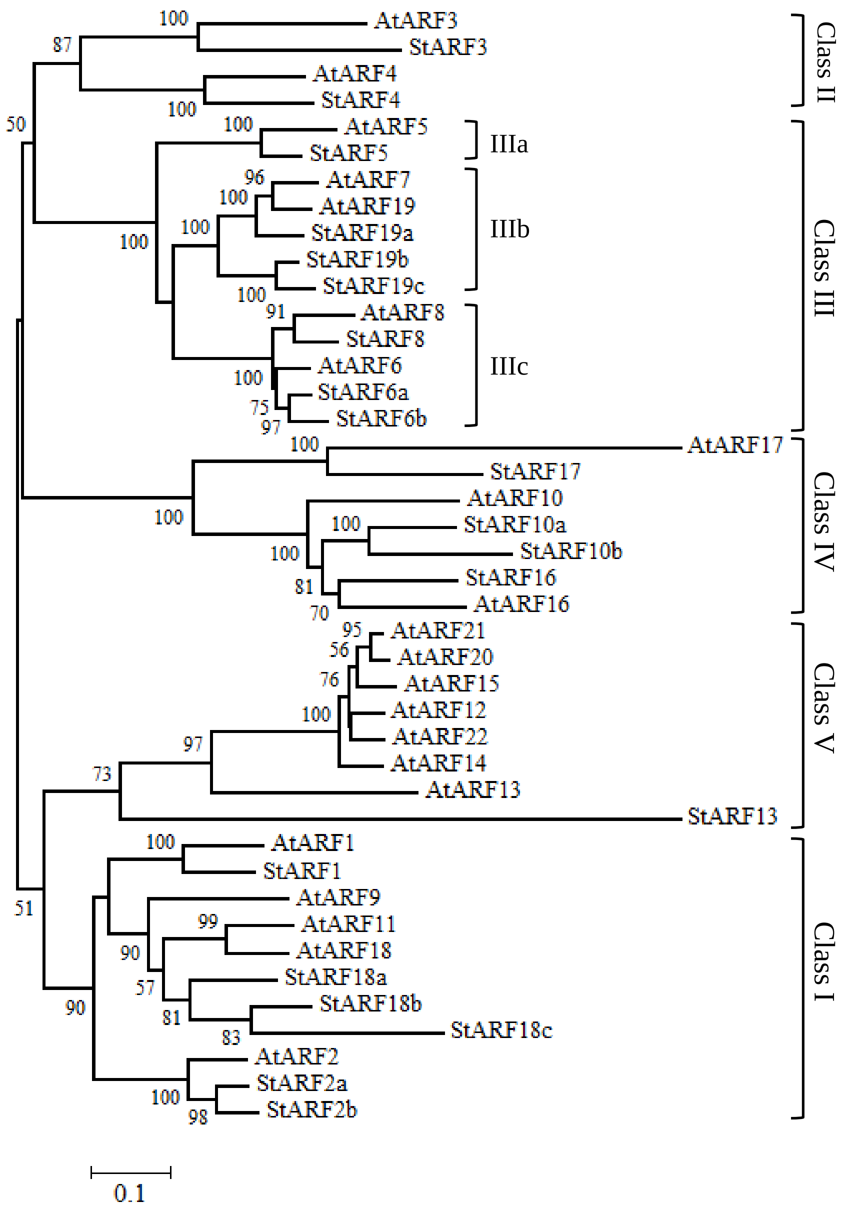

Supplementary Figure S1. Phylogenetic analysis of ARFs from Arabidopsis and potato. With the aid of MEGA program (version 10), Amino sequences of full-length putative StARF and AtARF proteins were aligned, and neighbor-joining tree was generated with 500 bootstrap replications. Bootstrap values are shown on branches.

Supplementary Table 1. Primers used in qRT-PCR experiments

Name	Sequences (5'→3')
StARF1-F	TAAAAGGGGCTCAAGCAGGC
StARF1-R	ACTGTTCTTGCTCTTCTTCAACA
StARF2a-F	ATCCAACTGCTTTGGGCAGA
StARF2a-R	ATGGACAGGCCAATGACCAA
StARF2b-F	TTAATCTCCATCCTCCGCCC
StARF2b-R	TGGGACAATAAACACATTTCACAA
StARF3-F	TGCACAAGTCTCACTAGTTCCT
StARF3-R	CGAGGGACAGAGAAACCACC
StARF4-F	TACTGGACGGCCATTTTGCT
StARF4-R	GGGCGGACTGGTTTATCACA
StARF5-F	AGCATATTGAACTTTGCCTTCC
StARF5-R	GCAGCTCTCGCAAACAGAT
StARF6a-F	CTCAGCAAGAGGAAGCAGGG
StARF6a-R	GTTGAGGCGGTAAACCAGGA
StARF6b-F	TGGGGTCAAGTCTCATTTGAA
StARF6b-R	TGAGCACAAGAGCTTGTAAGT
StARF8-F	AGGCTCAGGACACATGTGATTTA
StARF8-R	TGGTTCTGGAGAAACCTTTTCG
StARF10a-F	GTTCCTACTGCGATGGTGCT
StARF10a-R	TGACGAACATGTCCATGTCAA
StARF10b-F	TTGCGTTAGGGCCTCTTCTG
StARF10b-R	GATGGGGTCAGCAACCTGAA
StARF13-F	ACTTCGACTCTGTTCCCTGC
StARF13-R	TGGCTATCCAACTTTTCACCT
StARF16-F	CATAAAAAGCTCGTCGCGGG
StARF16-R	CAGGTCCACCTCCAATTCCC
StARF17-F	TGAAGGCACAAGCGGTAGAG
StARF17-R	ACTTGTCGGTCCTGAAGCAT
StARF18a-F	TGTTGACAACTTTTGGTGGGG
StARF18a-R	TCGCTCTTTTCCACTCAGCG
StARF18b-F	TCTATTCGCGCGTTAACCTGT
StARF18b-R	ACAAGTCTCACAGAAAGGGCA
StARF18c-F	ATCTCATGCTGTGACGACCC
StARF18c-R	CCAAGGCTCCCCTTGACATC
StARF19a-F	TGCTTCCAAAATTGCGACCT
StARF19a-R	CTTCATTGGTTGCGGCAGTG
StARF19b-F	TCCCGCCTCTGGATTACTCA
StARF19b-R	GAGTCACCAGCAAAGAGCCT
StARF19c-F	GCGGAATTTGCAGGTTGGTT
StARF19c-R	TCCTCTTGAACATGCCGTCC
StEF1α-F	AGGTTCTAGTTCTCTGCCTTGTATGTC
StEF1α-R	CGCCACCGCCTATCAAGTAC

Supplementary data. Alignment of deduced amino acid sequences of *StARF* genes. Alignment was generated using ClustalW program. The conserved N-terminal DNA-binding domain (DBD) and C-terminal domain (CTD) is underlined in blue and red, respectively.

1. *Marchantia polymorpha* ARFs

>Mapoly0011s0167

MSEASSITRHPYKANTGPLLKFQQSSDASSLPPMARPMASRQLATSHTAASNVSVAGDDGIDAELWYACAGPQKALPPV
GSVVAYLPQGHIEQVASFNNQELDAQIPRYNLPAVIPCMLNDIQLSADPDSDEVYATLTLCPMSEQHEDSSDCAEPPPP
PKRKSRSFTKTLTVSDTSTHGGFSVPRRAADDCLPKLDMSLNPPNQELVAKDLHGNEWRFRHIFRGQPKRHLLTTGWSV
FVSQKRLVAGDAVLFLRGENGQLRVGVRRAPRQQQLQPKVLTSPTMHIGVLAAAAHAATEKSRFSLIYNPRSCPSEFVI
PYSKYLKAVKSNFNVGQRFKMKFESEDPSDRRHTGTITGICDFDPARWPGSEWRSLQVNWDESSSSERQERVSPWEVEP
FSPSTTITPSVSTRKRLRPVTQPHSESVNRNAVETSKAQTQTMRLARAFHGGHEMLPSSAEEEDAESLSAKMSWIKRED
NFKSEAQSVGSRQGPDSWMSIRRPDPVQVPDMFRNLPASGVPDLRGMIGIERRQQEHLKFCVKQYRENKDEISGTTLQL
SSPRPPNLQNYVKSSTDLNLSVSSPASSNKGSSLLWSNSQSVTLPSYNGHESTNASSWLSFRPGQSDVAASSPHCTTLS
MSNLPPADSETSSHPSTPKSYLWEKRIRMEPDTNRAAAPVQSEQKCKIFGVPLDKPTPIVIPSQVPGSKAVRSTDDGSG
PSSSRGLEKVVSPSPTSSAVGGQEQDKGPQRSNKTSQNFQQGPVRSYTKIHKQGSFGRSIDVQSYDGYTDLLRKVENMF
ELNGELFDKKSGWQLVYTDHEDDVLLVGDDPWMEFVSCVRTLRLLSPGEASSSGKSGQSHDEDAGAGKDGAKRCDSSSP
SAGARGDDM

>Mapoly0019s0045

MYSCSPMRLSASGYAQHQTMTGEQRSLNSELWHACAGPLVSVPPVGSRVVYFPQGHSEQVAASTQKEADVHIPSYPSLP
SRLICLLDNVTLHADMETDEVYTRMTLLPMSGSPEKELVIVPDITRDTKQPTDFFCKTLTASDTSTHGGFSIPRRAAEK
VFPPLDYSQQPPAHPAQELVARDLHDQEWHFRHIYRGQPRRHLLTTGWSVFVSAKRLQAGDSVLFIRDDKGQLLLGIRR
ANRQQTAMPSSVLTSDSMHIGVLAAANHAAATNSRFTIFYNPRASPSEFVIPLAKYNKAIYHTQVSVGMRFRMVFETEE
SGVRRYMGTITGIGDVDPLRWPSSHWRSLKVGWDESTAGERQRRVSLWEIEPLTTPFLICPPLTLRAKRPRASSRGHSG
MSGEDEEGDGSVKKSSMWLRDDERDGLSNLPFRGLGMDPWTPRLPQSPDSVAPGTPTDFYRVMAAAALQEIRGNETSKQ
LLQRQPQQSLQNHQMQFRPQQQQPPLQQQEQQQQQQQQQQQQQQLNMQQQQQQVLQSPQPLPLPDVTGPLLQLSSSRPQ
SPMQLSTLRASSGYTDSDVHLSPASTASGSFPLQSLLSRTQQGSVLTTEDTNQFSNLLRSNQSAMQQATMLPGSAMVGR
DSPVSSAWYSMRDSTTSDCAHPSSRMGRADSSPSSAPTYALSSNEPCQSGLTALPMQNPTYAVFRDNSQEQDQVQSDPR
SHLLFGVSIDQMPNGAGGLGSRGFGKAKDNQARFAGGSLLPAPYCSSAGQDLPISPGIISHGSINDSQFMQRGFMAPVS
SPQRSYTKVYKLGSVGRSLDVAQFTNYTDLRVHLARMFGLEGQLEDPQRSGWQLVFVDNEQDVLLVGDDPWDEFVNCVR
SIRILSPSEVMHMSQEGLELLNIVPPTAPRPTSSGSEDGATMPAGFEKSCGNDAQRGCP

>Mapoly0043s0098

MPGPSPGCGTMSGTNIKMEKSEESMGGGGKGWGGGRDRDSSSDGGGGSGENTGLDPQLWHACAGGMVQLPPVGAKVIYF
PQGHGEQAATPPEFPRMMGPQGTIGCRVVSVSFLADTETDEVYARIRLQPLEREAAMSIADSTLDADGGPSSPPPEKPA
SFAKTLTQSDANNGGGFSVPRYCAETIFPPLDYSIDPPVQTVLAKDVHGERWKFRHIYRGTPRRHLLTTGWSTFVNQKK
LVAGDAIVFLRTASGELCVGVRRSMRGTGGADSSTWSGGSSTSHHRPNRWEVKGTESFSDFLGNDSAAGGGSVSSAGSA
AGPGGPRAGPGGSNSGPGIGIPGPSTTSSFARNRARVTAQSVLEAASLAVQGQPFEVVYYPRASTAEFCVKAQAVKAAL
DHTWFPGMRFKMAFETEDSSRISWFMGTISAVQPADSLWPKSPWRVLQVTWDEPDLLQGVSRVSPWQVELVSTLPMQLP
PFSLPKKKLRAAQPSDMNMQGQGLMGMPITLPSVFGQINPWAHGLTMEEVTAGMQGARHDRVFGLALSEKFRPGKLPGG
FFSAAEGYYPDHAGRGGGGEPHLSAFPLQDRASNISSLISSLGSVPPSGDHGSAGALLVPAGSWSGGSANNKSASTQLV
LFGQAINTDSNKSQPQHSGGSSCDGPSLQHLKEESSGKRKDSPSESNQNENFERGQKYMSGSLSNGKQSDLIVGEFQKW
VPGLDKERGAGEKLAASPEILQSPQGAW

>Mapoly0160s0029

MCEPLVSVPTVGSRMIYFPQGHSEQVAASIQKEADVHISNYPSLPLRLNCLLDNVTLHADMETDEVYTRMTLLPMSGSP
EKELWYFRHIYRGQPRIHLLTTRCSVFVNAKRLQAGDFISVEMRFKMVFETKESDVQRYMETITGIGDVGPLRWPSSH

2. *Physcomitrella patens* ARFs

>Pp3c1_14440V3.1.p

MVAVSTQKEADTHIPNYPNLRPHLVCTLDNITLHADLETDEVYAQMVLIPSQDPDKETMLLPDAVVQNKQPTEYFCKTL
TASDTSTHGGFSIPRRAAEKVFPTLDWHFRHIYRGQPRRHLLTTGWSVFVSAKRLQAGDAVLFIRDDKGQLLLGIRRAN
RLQTMMPSSVLSSDSMHIGILAAASHAAQTSSRFTIFYNPRQSPSEFVIPLAKYQKAVYSTQVTVGMRFRMVFETEEST
VRRYMGTVTGIGDLDPVRWPNSHWRSLKVGWDESTAGERQLRVSLW

>Pp3c1_14480V3.1.p

MYSHASMRGSGYGPPAMDQGERRINSELWHACAGPLVSLPPVGSQVVYFPQGHSEQVAVSTQKEADTHIPNYPNLRPHL
VCTLDNITLHADLETDEVYAQMVLIPSQDPDKETMLLPDAVVQNKQPTEYFCKTLTASDTSTHGGFSIPRRAAEKVFPT
LDYNQQPPAQELVARDLHDQDWHFRHIYRGQPRRHLLTTGWSVFVSAKRLQAGDAVLFIRDDKGQLLLGIRRANRLQTM
MPSSVLSSDSMHIGILAAASHAAQTSSRFTIFYNPRQSPSEFVIPLAKYQKAVYSTQVTVGMRFRMVFETEESTVRRYM
GTVTGIGDLDPVRWPNSHWRSLKVGWDESTAGERQRRVSLWEIEPLTTPFLSCPPPLASRSKRARGIHGEDEDLEALMK
KSQLWPNSCDTLGPLSFGMEQPWMRMSQQRAGNEYYRTFAAATALQEFRDPSKQVVTDSMPNSQILYRQPQPQLQNQHN
QQLMPQMNDVPGPLLQLSTTQASMDLGSSVRPSSSYSDSEIHLPSPSSRSFSIQGMMGRTLGNVPTNSGDGNQFPSLMR
PNQTGLSPCGLIAGNHQVSPSWFPTLQNPSQAESQSTASPRLHRVEVPNGGGVPGSYGFTQHGEPDVQPSLEHLPTYGF
RSNGAESDQLQTDRSHLLFGVPIDQPLGTSSGLPSPNFGKAKDLSGNTMLPGSFCSPATPGDSGIMPSERIDDNVMLPR
GVGWPTVQPAPPVRSFTKVHKLGSVGRSLDINKFSNYAELRKELAHMFHLECLMEDPQQSDWLIVYVDNENDTLLLGDG
PWEAFVSCVRSIKILSPVEVAQMSQEMPAAVLGQQLRPSNSNSEDAPNSQAGGGALDH

>Pp3c13_4720V3.1.p

MYSSSPARLSASGYGPATMDPGERRSLNSELWHACAGPLVSLPPVGSRVVYFPQGHIEQVAASTQKDADAHIPNYPSLP
SKIICLLDNVTLHADPETDEVYAQMILLPIQISEKEALLSPDLEVVNKQPTEYFCKTLTASDTSTHGGFSIPRRAAEKV
FPPLDFTRVPPAQELVARDLHDQEWHFRHIYRGQPRRHLLTTGWSVFVSAKRLQAGDSVLFIRDDKGNLLLGIRRANRQ
QTVMPSSVLSSDSMHFGVLAAASHAAATSSRFKIFYNPRQSPSEFVIPLTKYHKALYNTQFTVGMRFRMVFETEESSVR
RYVGTITGLGDLDPIRWPKSHWRSLKVGWDESTAGERQHRVSLWEIEPLTTPFLLCPPPLALRSKRPRGMPGEDDLEML
MKKSHMWPNDSDPLGLQKFGGLAMNTSWMRLPQQNVGSMVPSSQNEQYRALAAAALQEIRTADSSKQLLAQSPLLSQLH
FRQHQSHPQPQQLMQHINDVPGPQLQMSTCQTQTTDLGNPARSSSYRESDVQLGPPATTSNSFSYQEMMGRAPSSNPLT
SNGGHQLTSMMRTSQNGLQPCGTMQEFIQGSTPWFPNQPDSQSLILSQHTRVEAPSGGVPGSFALPQNNESALQDVTGK
NVLLSAYGPPATPDSMGNGMMSGEVLDENGLFQRNTGWPPASSQRTFTKVHKLGSVGRSLDVRIFNTYAELRKELAKMF
HLDCLMEDPPTSGWQIVFVDNENDTLLLGDDPWEDFLNCVRSIKILSPSEVTQISQDQLKMLETVPVQHLQPQRLISSD
SGEVYPQSSLPDPSSGL

>Pp3c1_40270V3.1.p

MEVVAEEESRPCGATVWSGFLRVERRSPTSELWHACAGPLVSLPPIGSRVVYFPQGHTEQVAASTQREAETHIPNYPSL
PSRLVCLLDNVTLHADLETDEVYAQMTLIPVPPANEKEALMSPDIGIRSRQPTDYFCKTLTASDTSTHGGFSIPRRAAE
KVFPPLDYSQTPPAQELKARDLHDQEWHFRHIYRGQPRRHLLTTGWSVFVSAKRLQAGDAVLFIRDDKGQLQLGIRRQN
RQQTVMPSSVLSSDSMHIGVLAAANHAAATSSRFTIFYNPRQSPSEFVIPVAKYQKAICNLQVSVGMRFRMVFETEESS
VRRYMGTITGMGDLDPIRWPNSHWRSLKVGWDESTAGERQRRVSLWEIEPLTTPFLLCPPPLTFRAKRPWGGRVDEEMD
SMLKKASFWSGDSGSHMDALGALNLRNFGMSSWMRTPQQRVEPGLPAQQNEYYRAFAAAALQEIRCSDASKHAMSHAQP
SLSTSQIEFRSQSPQSNQHTAQHIPNTAGPVLQLSSSRPESPLDVGMNMAQCSGYSESDTHMTSSAYTPGSYPLHSMLG
RTHLGCENGQMTYMMRPTQSAQQSQPESIIHGGSVREPQFSSSWYPSNRDTSEHDVSARMNQLDTSPTSRVSSCFPFPQ
ESQVNGQSGLTGLPVPTSSFVYRENGQEQDSVQSDRHLLFGVSIEQPLVGSNSVTSLQPHAFAKSKDPQSRFSGNTVLQ
GSYYPSGNADIPTMNGVGLDENGIFMRNASWSAMAPTSRTFTKVHKLGSVGRSIDVQKFQNYSELRVELARLFNLEGLL
DDPQRSGWQLVFVDNENDTLLVGDDPWEEFVNCVRSIKILSPNEILQMSQEQLEILNSVPMQQRPTCSNSEDARTQTSP
VNTSNLSMEHGHSGGR

>Pp3c14_16990V3.1.p

MYLCNERLPSVGPSVGSMAQPAEKVEKRSLNSELWHACAGPLVLLPPVGSRVVYFPQGHTEQVAASMQKEVDAHIPNYP
NLPSRLVCLLDNVTLHADIETDEVYAQMTLIPVPPTNEKDAHMSPDIEMRSRQPTEYFCKTLTASDTSTHGGFSIPRRA
AEKVFPPLDYAQIPPAQELKARDLHDQEWHFRHIYRGQPRRHLLTTGWSVFVSAKRLQAGDAVLFTRDNKGQLQLGIRR
QNRQQRVMPSSVLSSDSMHIGVLAAANYAAATSSRFTIFYNPRQSPSEFVIPVAKYQKAICHLQVSVGMRFRMVFETEE
SSVRRYMGTITGMGDLDPIRWPNSHWRSLKVGWDESTAGERKKRVSLWDIEPLTFPYVLCPQPLAFRAKRPRGGRDEET
DSMLKKSSFWSGENSTDALGRLTFRTPSLDSWMRPKQPGLPMQENEHYRALEAAALEEIRTTSTAKNPMSRVQSSIPSS
QMQFQPPHVLRQSSQHGELHMPEASGPLLQLSSSRPQSPFEVKVPSKIYLEPLFMEAVQLCVTLRYLHLGIHLIEI

>Pp3c16_6100V3.1.p

MYGYGGGMMRGPIKNQGLSKMDELDYELWHACAGPLTSVPPVDSLVMYWPQGHIEQVIACTPDSAGAADVREASSHFKL
PSHLLCKIGQLELQADPHTDEVFAQMDLLPQDEGSLTKEMKDATHVAKQNNVKMFCKTLTASDTSTHGGFSVPRRSAED
CLPSLDYTANPPCQELVAKDLHGHEWKFRHIYRGHPRRHLLTTGWSAFVSAKKLVAGDTVIFLRGENGQLRVGVRRASK
QQPQARSTNFSSTNLHLGVLAAASHASREGMRFSVIYNPRTSPSEFVIPYNKFLKAMDNKLAVGSRFRMKFESEESSER
RFAGTITEVSDADPVRWPNSLWRSMKVEWDEVISASERHERVSPWEIEPFVQISTLPPPQLGPRQKRRLPTLVKESPLG
TSQSVLDSYQSNKLAKILQGFDPRNESSMEDDEDGDVACSGNTSWNVKPEPPPKGLPQAEIVQQSWFPRPDISSYGIPG
DPYQPLISPRVSSVTSFAPPLAAESMDLQLSVKSARSEQAPSKDYSSAPWSDFSGICPSNRTGASFPVRESPCPSINPS
WLYSASTSRAVFERSILTNSLHSESAPEMLPPQAVPAFEVRDKAQLEKVDTTPGLVQPCKLFGFNLADKIVPTLAPLPP
SQGDVSEGLVDSIRTDSTSPTSGGPFGSKVAAESSQVSQAHAAPMRSGIKVYQQGKVGRTIDLRKCESYDGLRRVLANL
FNLQGQLDDVTKGWQLVYTDHENDVLLVGDDPWEEFCGCVRSLKILSPQDAAGQTVGRIPASSCEEDDEWH

>Pp3c17_19900V3.1.p

MYLSSERPPSLGPIMTSMAQPMEQVERRSLNSELWHACAGPLVSLPPVGSRVVYFPQGHTEQVAASTQKEADAHIPNYP
NLPSRLVCLLDNVTLHADLETDEVYAQMTLIPVLPANEKEALISPDIGMRSRQPTEYFCKTLTASDTSTHGGFSIPRRA
AEKVFPPLDYTQTPPAQELKARDLHDQEWHFRHIYRGQPRRHLLTTGWSVFVSAKRLQAGDAVLFIRDDKGQLQLGIRR
QNRQQTVMPSSVLSSDSMHIGVLAAANHAAATSSRFTIFYNPRQSPSEFVIPVAKYQKAICSLQVSVGMRFRMVFETEE
SSVRRYMGTITGMGDLDPIRWPNSHWRSLKVGWDESTAGERQRRVSLWEIEPLTTPFLLCPPPVAFRTKRPRGGRDEEI
DSTSKKSSFWSGDEDTGVLGGLNFRNLSMDSWMRPQQPGLPTQQNEYYRALAAAALQEFRTPDCSKHPTSRSQPSISPQ
MQFRSQPQMQSGHHGVQHVSEACGPLLQLSSSRPQSPLEVGMNMPQCSGYSEEDIQMASSPSASGSYPLHSMLGRTHLG
CETGQVGLMMRPTQNAQQSQSGPILHGGSTIGHEPQVSSSWYPSNRDPNQHDVSARMNQLDTSPTSRVSGSFSLSQQSE
GNGQSGLTGMPVPASSFMFRESGQEQDSVQSDRHLLFGVSIEQQPLGASNPVASIHSQSYPKNKDVHNRFSGNNMLQGS
YCSSTMPDISTMNGVGLDENGMCQRGAPWATMSPAPVRTFTKVHKLGSVGRSIDVQKFQNYSELRAELARLFNLDNLLD
DPQRTGWQLVFVDNENDTLLVGDDPWEEFVNYVRSIKILSPNEIQQMRQEQLEILNTVPMQQRPTCSNSEDARTQTSPP
NTSTLSLEHGHSGPG

>Pp3c2_25890V3.1.p

MYSHVPMRGSGFASSTIVQGEKRINSELWHACAGPLVSLPPVGSQVVYFPQGHSEQVAVSTQKEADIHIPNYPNLRPHL
ICTLENVTLHADLETDDVYAQMVLIPTQDPEKETMLLPDVVVQNKQPTEYFCKTLTASDTSTHGGFSIPRRAAEKVFPT
LDYTQQPPAQELVARDLHDQDWHFRHIYRGQPRRHLLTTGWSIFISAKRLQAGDAVLFIRDDKGQLLLGIRRANRLQTI
MPSSVLSSDSMHIGILAAASHAAQTSSRFTIFYNPRQSPSEFVIPSAKYQKAVYSTQITVGMRFRMMFETEESTVRRYM
GTVTGIGDLDPVRWPNSHWRSLKVGWDESTAGERQRRVSLWEIEPLTTPFLICPPPIVLRSKRARGIHGEDEDLETLMK
KSPMWPSLGFGTDSPWMGILPQRPGHGVTSSLNEYNRTLAVAASQEFREPSKQVVTESMSNPQNLYRQQQLQTQQHSHR
NPQSIAQINDVPGPLLQLSTSQASIDLGSSVRPISSYSDSEIYVSSPSPRPFSTQAMMSRIPGNVPVSSADGNQFPSLM
RTNQNGLPLYGSVTGNHQVSSSWLPTLQNQSQAEPQSASPRFHRAEASIGGGVPGLYGPIQHGEAGDAGILPNEALDEN
IMLQRNVGWPAVATAPPVRSFTKVHKLGSVGRSLDINKFSNYVELRKELAHMFHLECLMEDSQQSSWKIVFVDNENDTL
LLGDEPWEEFVSCVRSIKILSPAEVAQMNQHVLAAVSGQHLRPSNSNSEDTPSSQAGVGVVDH

>Pp3c26_11550V3.1.p

MYSNPPARLSASGHVSSTMDPGGRRSLNSELWHACAGSLVSLPPVGSRVVYFPQGHIEQVAASTQKEADVPIPNYPSLP
SRLFCLLDNVSLHADHETDEVYAQMTLLPIQNSEKEALLAPDSVIPNKQPSEYFCKTLTASDTSTHGGFSIPRRAAEKV
FPPLDFTKSPPAQELVARDLHDQDWHFRHIYRGQPRRHLLTTGWSVFVSIKRLQAGDSVLFIRDDKDHLLLGIRRANRQ

QSVMPSSVLSSDSMHFGVLAAASHAAATSSRFKIFYNPRQSPSEFVIPLAKYQKALYNTQVTLGMRFRMAFETEESNVR
KYMGTITCIGDLDPARWPKSDWRSLKVGWDESIAGDRQLRVSLWEIEPTPTPFLLCPPPVALRSKRPRGMQEEEDALEM
LMKKSHMWPHGSDPSVSLKVGGLRLDPLWMRLPQPRLGPMVSSPQSGYYRALAAAALQEIRSVDPPTQLLPQSASSSQL
FFGQPQSQSQPQQLMQHINDIPGPLLQLTTSQTQTVDLANPVGPSSSYRESDMHMTSPTTMSNSLALQGMMRRASTSAT
LSFSEGNQITSLMRKSHNGLQPYGTVQGVIQASTSWFSNQSDSQALISSQQSRVEASSGGVPESYALPQNNESSLHQGL
GHQLPFGFRSNDQDSDQLQADRSHLLFGMSIDQPLGGSSVVSPQSYRKSKDDTGNNMLLTAYGPPVTPDSMNNGILSGE
GLDGNGLFQRNSGWPAMPVAIPPRTFTKVHKLGSVGRSLDVRNFSNYTELRQELARRFQLDCLMEDPSSSGWQIVFVDN
EDDTLLLGDDPWE

>Pp3c27_60V3.1.p

MYGYGGGMMRGPMMNQGLSKIDDLDYELWHACAGPLTSLPPVDSLVIYWPQGHIEQVIACTPDSAGAADVREASSHFKL
PSHLLCRIGQLELQADPNTDEVFAQMDLLPQDEGSVTKEMKEAPSAIKQSSVKMFCKTLTASDTSTHGGFSVPRRSAED
CLPLLDYNMNPPCQELVAKDLHGQEWKFRHIYRGYPRRHLLTTGWSTFVSAKKLVAGDTVIFLRGENGQLRVGVRRASK
QQPQAHSTYFSSTNLHLGVLAAASHASREGMRFSVIYNPRTSPSEFVIPYHKFLKAMDYNLAVGSRFRMKFESEESSGR
RYAGTITEVNDADPLRWPNSLWRSMKVEWDEVISASDRHERVSPWEIEPFVPISTLPPPLGGARQKRRSPTLVKDPLLG
TSQSVLDSYQSNKLFKVLQGFDSRNEFSMEDEEDEDVVCSRNTSWNVKPEPPPKTVPQAMPAQQSWFPRADMPSFGVPV
APHQPLISPRVPSENMNLQLSVNPASSEQAPGNDHSSASWSDFSGGCLNFNWLYNASTPRPVFERSNLPTTSLNNESAS
KMLPPQVIPPFEARDKVELVKLRTTPALVQPCKLFGFDLADKKVPSSVTLAPSHGEVSEVTVELLRTDLTSGGHLGNKL
TAESCQATQAHVTPMRSGIKVYQPGKVGRTIDLKKCESYTGLRRVLANLFNLQGQLDDVTKGWQLVYTDHENDVLLVGD
DPWEEFCGCVRSLKILSPQDVAGQSVGKFPTSSCENDDGRQ

>Pp3c4_12970V3.1.p

MLEPGLRLSMGIKTEKLDDSGGRERSMASGVGESVDRLDAQLWHACAGGMVQLPQVGAKVIYFPQGHGEQAATTPDFSA
SMGPSGTIPCRVVSVNFLADTETDEVFARMRLQPEGLHGLNDMTEEAPSSPPPEKPASFAKTLTQSDANNGGGFSVPRY
CAETIFPPLDYSSDPPVQTVLAKDVHGDVWKFRHIYRGTPRRHLLTTGWSTFVNQKKLVAGDAIVFLRSASGELCVGVR
RSMRGASGGDSSTWHSSANATRASRWEVKGTESFSDFFAAVGDNGHGGSSNGVSRSGSQGASTTSSFARNRARVTAKSV
LDAAALAVAGKPFEVVYYPRASTAEFCVKAGLVKQALDHTWYAGMRFKMAFETEDSSRISWFMGTIAAVKPADPLLWPN
SPWRVLQVTWDEPDLLQGVSRVSPWQVELVATLPMQLPPFSYPKKKLRAVQPQELQLQAPGLLNLPLAGSSSFAGQLPT
PWGGPALLENASAGMQGARHDRFNGPPSMDFRYSNYKRAREHPSENQYAEQGQTSPAGSARVVLSDPLCGDSHHQFSFL
SSGQAHNGGQQQQQQQSSQMSGSGLTMGLMPGGSPTRDDGGSNSKSKLKSSPAPTTFLLFGQSIDPSSNSKAAQEQCV
ASASSSVEGSSLFQEGLRVSPTSYSSSDNTLEHKDRMRRFNADLNGASGGTGDGAMSRYRQNEGGPWPELSIGTEVGSL
KWFKEQRLEKEKGSNEALQHCKVFREGDEVGRTLDLANFKSYEEVYDRLAGMFSVPAASFKNRVVYQDGEGCTLPVGAE
PYG

>Pp3c4_13010V3.1.p

MLEPGLRLSMGIKTEKLGDSGGRERSMASGVGESVDRLDAQLWHACAGGMVQLPQVGAKVIYFPQGHGEQAATTPDFSA
SMGPSGTIPCRVVSVNFLADTETDEVFARMRLQPEGLHGLNDMTEEAPSSPPPEKPASFAKTLTQSDANNGGGFSVPRY
CAETIFPPLDYSSDPPVQTVLAKDVHGDVWKFRHIYRGTPRRHLLTTGWSTFVNQKKLVAGDAIVFLRSASGELCVGVR
RSMRGASGGDSSTWHSSANATRASRWEVKGTESFSDFFAAVGDNGHGGSSNGVSRSGSQGASTTSSFARNRARVTAKSV
LDAAALAVAGKPFEVVYYPRASTAEFCVKAGLVKQALDHTWYAGMRFKMAFETEDSSRISWFMGTIAAVKPADPLLWPN
SPWRVLQVTWDEPDLLQGVSRVSPWQVELVATLPMQLPPFSYPKKKLRAVQPQELQLQAPGLLNLPLAGSSSFAGQLPT
PWGGPALLENASAGMQGARHDRFNGPPSMDFRYSNYKRAREHPSENQYAEQGQTSPAGSARVVLSDPLCGDSHHQFSFL
SSGQAHNGGQQQQQQQSSQMSGSGLTMGLMPGGSPTRDDGGSNSKSKLKSSPAPTTFLLFGQSIDPSSNSKAAQEQCV
ASASSSVEGSSLFQEGLRVSPTSYSSSDNTLEHKDRMRRFNADLNGASGGTGDGAMSRYRQNEGGPWPELSIGTEVGSL
KWFKEQRLEKEKGSNEALQHCKVFREGDEVGRTLDLANFKSYEEVYDRLAGMFSVPAASFKNRVVYQDGEGCTLPVGAE
PYG

>Pp3c5_9420V3.1.p

MHYGGMHGGMRSFDEGATKMDELNCELWHACAGPLTQLPPVDSLVMYWPQGHIEQVVACTPDSAGAADVYQASKQFSNL
PAHLLCRISKIELQADPQTDEVFAQMDLTPQYETELSKETKDAPSPIQQSNVRSFCKTLTASDTSTHGGFSVPRRAAEE
CLPLLDHNMVPPCQELVAKDLHGKDWSFRHIYRGHPRRHLLTTGWSVFVSQKRLVAGDTVIFLRGENGQLRVGVRRASK
QQPQARSTHFSSANLHLGVLAAASHAATERLRFSVIYNPRTSPSEFVIPYHKYLRSEDNNLTVGSRFKMKFETEESTER

RYSGTIVEISDVDPLKWPSSAWRSMKVEWDESASERHERVSPWEIEPLVPISTLPTPPVGPRPKRRPPTFVTDSATLGP
SQSVLDPFQSNKLARVLHSHDPRNEPSIGDDDDGDAESSRTSSLVVKQEPPPRNGHQIWLQRSDVASYGIPPSPYVNRR
LSSVPPFAAPLPENTTELQLSVNSGSLDQAPSNEDSSVSWASYMGTGAYQFRDPSCNKILPSWLTNSKSANLTSPPVPA
RSQLPITSLNNDPKVLHAHNLSFELWETVEQEQLNASPALEQQCKLFGFNLADKVVPTPVSSAPSLCEDSEGSGPWSSS
DHTSSTSADTRVGMIVTGTYQPLVAPVRSGTKVYYSGKVGRTIDLKKCESYAALRRMLASLFGLEGQLDDVTKGWQLVY
TDHENDVLLVGDDPWEEFCNCVRSLKVLSPQDAAGQSVGKYPMTNCDEDDDWQSAVQSSG

>Pp3c6_21370V3.1.p

MQYYGGMRGFDEGSTKMDELDCELWHACAGPLTQLPPVDSHVMYWPQGHIEQVVACTPDSAGAADVYQASKQFSNLPAH
LLCKISKIELQTEFTKEMKDAPPPTMQKNVRSFCKTLTASDTSTHGGFSVPRRAAEDCLPLLDHSMNPPCQELVAKDLH
GKEWNFRHIYRGHPRRHLLTTGWSVFVSQKRLVAGDTVIFLRGENGQLRVGVRRASKQLPQTRSTHFSNANLHLGVLAA
ASHAATERLRFSVIYNPRTSPSEFVIPYHKYLKTKENNLTVGSRFKMKFESDESTERRYSGTIVEVSDADPLKWPNSAW
RSMKVEWDESASERHERVSPWEIEPFVPISTLPTPSVGPRPKRRPPTFVTDSSPQGTSQSVLDPQQSNKLARILHSHDP
RNELSIGDDDDGDIESSRTSSLAVKQEQPPRTGQQIWLQRPDVSSCGMSASPYSSPRLPSMAPFAPLPADKTDLQLSVK
SGSLDQTLSNDVSSVSCAPYVGPGNHSTAAHHFREPSCNKILPSWLTYPKSTDGTGVTSLPGSGRTQLPITSLNNDPKL
TPAQNLSFGSWDKVEQEKVHTSPALEQQCKLFGFNLVDKAVLAPVSSAPSHCEDSEGSGPWSSTDLTSPTSVDTHVSKL
ANGAYQPPTVPVRSGTKVNYYGKFGRTVDLKKCDSYAALRRMLATLFGLEGQLDDVTKGWQLVYTDHENDVLLVGDDPW
EEFCNCVRSLKILSPQDAAGQNVGKLPMNNCDEDDDWLSAVQSSG

>Pp3c6_26890V3.1.p

MPGPVPLLSMSVKSESLDDIGGHEKKSVTGSEVGGLDAQLWHACAGGMVQLPHVGAKVVYFPQGHGEQAASTPEFPRTL
VPNGSVPCRVVSVNFLADTETDEVFARICLQPEIGSSAQDLTDDSLASPPLEKPASFAKTLTQSDANNGGGFSIPRYCA
ETIFPPLDYCIDPPVQTVLAKDVHGEVWKFRHIYRGTPRRHLLTTGWSTFVNQKKLVAGDAIVFLRIASGELCVGVRRS
MRGVSNGESSSWHSSISNASTIRPSRWEVKGTESFSDFLGGVGDNGYALNSSIRSENQGSPTTSSFARDRARVTAKSVL
EAAALAVSGERFEVVYYPRASTAEFCVKAGLVKRALEQSWYAGMRFKMAFETEDSSRISWFMGTIAAVQAADPVLWPSS
PWRVLQVTWDEPDLLQGVNRVSPWQLELVATLPMQLPPVSLPKKKLRTVQPQELPLQPPGLLSLPLAGTSNFGGHLATP
WGSSVLLDDASVGMQGARHDQFNGLPTVDFRNSNYKRPREFSRDNQYQIQDHQVFHPRPVLNEPPATNTGNYFSLLPSL
QRRPDISPSIQPLAFMSASGSSQLETSSTKTAATSFFLFGQFIDPSCTSKPQQRSTVINNASVAGDGKHPGTNNSSSDN
KSEDKDNCRDVQPILNGIAVRSGFRADIAAKKFQQSDSAHPTEASRGSQVSSLPWWQTQDAHKDQEFHGDSQTPHTPAS
GSQ

3. *Oryza sativa* ARFs

>OsIBCD000889

MSSQGAGGGVGDPELFAVLWRACAGPLVEVPQRDERVFYFLQGHLEQLQEPTDPALLAEQIKMFQVPYKILCKVVNVEL
KAETETDEVFAQITLQPDPDQENLPTLPDPPLPEQPRPVVHSFCKILTPSDTSTHGGFSVLRRHANECLPPLDMSMATP
TQELITKDLHGSEWRFKHIYRGQPRRHLLTTGWSTFVTSKKLISGDAFVYLRSETGEQRVGVRRLVQKQSTMPASVISS
QSMHLGVLASASHAIKTNSIFLVYYRPRLSQSQYIVSVNKYLAASKVGFNVGMRFKMSFEGEDVPVKKFSGTIVGEGDL
SLQWSGSEWKSLKVQWDEVTNVNGPERVSPWEIETCDGTAPAINVPLQSATKNKRPREPSETIDLQSLEPAQEFWLSGM
PQQHEKTGIGSSEPNCISGHQVVWPGEHPGYGAVSSSVCQNPLVLESWLKDFNSSNKGVSPTLSEISQKIFQVTSNEAR
IATWPARSAYQAEEPTSKLSSNTAACGYRTEEVAPNASKVVEGKKEPAMFRLFGVDLMKCTSISTTTDDKSSVGAGEAS
AKGTGSHEDSGQLSAFSKVTKEHIAADESPQEIQSHQNYTARTRIKVQMHGNAVGRAVDLANLDGYEQLMNELEEMFNI
KDLKQKWKVAFTDDEGDTMEVGDDPWLEFCQMVRKIVLYPIEDEKKIEPHPKLLSSANPEQDQKTGF

>OsIBCD002863

MVGIDLNTVEEEEDEEEGGATGTVTAPAEARAGGAVCLELWHACAGPVAPLPRKGSAVVYLPQGHLEHLGAAPGPAAVA
AVPPHVFCRVVDVSLHADAATDEVYAQVSLVADNEEVERRMREGEDGEGEDAVKRPARIPHMFCKTLTASDTSTHGGFS
VPRRAAEDCFPPLDYSLQRPSQELVAKDLHGTEWRFRHIYRGQPRRHLLTTGWSGFINKKKLVSGDAVLFLRGEDGELR
LGVRRAAQLKNVSPFPALHNQISSTSSLSEVAHAVAVKSIFHIYYNPRLSQSEFIIPYWKFMRSFSQPFSVGMRFKLRY
ESEDASERRRTGIIIGSREADPMWHGSKWKCLVVKWDDDVECRRPNGVSPWEIELSGSVSGSHLSTPHSKRLKSCFPQV

NPDIVLPNGSVSSDFAESARFHKVLQGQELLGLKTRDGTVNTASQATEARNFQYTDERSCSINMSNNILGVPRLGVKTP
SGNPGFSYHCSGFGESQRFQEVLQGQEVFRPYRGGTLSDACIRGSGFRPPDGNHASGAAFKWLAPQGCDHHGITTSVLP
QASSPSSVLMFPQTSSKMPGLEYIYGCLDRNENSRHFKIGPTQDMARTDQTLRLWPHLISGKVLDECTRNEKLHSPVGG
AEHESNTNKCLNTNGCKIFGISLTEKAQAGDEVDCGNASYHSRLQSLKPQMPKSLGSSCATVHEQRPVVGRVVDISAVN
TMI

>OsIBCD003335

MGIDLNYTASGGEEDAPAPAPVCRDLWHACAGPVVSLPRRGSAVVYLPQGHLSAAGAGGRIRGEVAVALPPHVACRVVD
VELCVSEPLSLVADAATDEVYARLALRAEGEVFERNLHGGGIEREDDMEDGDEERKSRMLHMFCKTLTASDTSTHGGFS
VPRRAAEDCFPPLDHKQLRPSQELVAKDLHGAKWRFRHIYRGQPRRHLLTTGWSSFVNKKKLVSGDAVLFLRGDDGELR
LGVRRATQLKNEAIFKAFSSESSKMRTLSAVADSLKHGSVFHICYNPRATASEYVVPYWKFVKSFNHPVCIGMRFKFHY
ESEDVNERRSGMIAGVSEVDPIRWPGSKWRSLLVRWEDATDCNSQNRVSPWEIEIVGGSISVAHSLSASSSKRTKLCPQ
GNLDVPALYGNGRPDSVETEKFPRVLQGQELMGSRTHRVTCSPQSIDITKSKSFDAWRFLTDTRSCMLGSSTSRLPVQY
SGYTHQSVSFGESIGFPEVLQGQEISQTVPPIQGMLPDACSAKSRYELKNYVCTPATMNGLSSANEGYCLSLSTVPPSP
PSSLMLYQTGVPQLELASKNNDKSGNDSQPALRQHKLLSETSWDQFKIGKASTPGNATKPGNGGREVDRTSCRLFGFSL
TEKIIPTDKDGEKEIVRINEAIADVK

>OsIBCD004370

MAPPPPPQGSSTGDPLYDELWHACAGPLVTVPRVGDLVFYFPQGHIEQVEASMNQVADSQMRLYDLPSKLLCRVLNVEL
KAEQDTDEVYAQVMLMPEPEQNEMAVEKTTPTSGPVQARPPVRSFCKTLTASDTSTHGGFSVLRRHADECLPPLDMTQS
PPTQELVAKDLHSMDWRFRHIFRGQPRRHLLQSGWSVFVSSKRLVAGDAFIFLRGENGELRVGVRRAMRQLSNVPSSVI
SSQSMHLGVLATAWHAINTKSMFTVYYKPRTSPSEFIIPYDQYMESVKNNYSVGMRFRMRFEGEEAPEQRFTGTIIGSE
NLDPVWPESSWRSLKVRWDEPSTIPRPDRVSPWKIEPASSPPVNPLPLSRVKRPRPNAPPASPESPILTKEAATKVDTD
PAQAQRSQNSTVLQGQEQMTLRSNLTESNDSDVTAHKPMMWSPSPNAAKAHPLTFQQRPPMDNWMQLGRRETDFKDVRS
GSQSFGDSPGFFMQNFDEAPNRLTSFKNQFQDQGSARHFSDPYYYVSPQPSLTVESSTQMHTDSKELHFWNGQSTVYGN
SRDRPQNFRFEQNSSSWLNQSFARPEQPRVIRPHASIAPVELEKTEGSGFKIFGFKVDTTNAPNNHLSSPMAATHEPML
QTPSSLNQLQPVQTDCIPEVSVSTAGTATENEKSGQQAQQSSKDVQSKTQVASTRSCTKVHKQGVALGRSVDLSKFSNY
DELKAELDKMFEFDGELVSSNKNWQIVYTDNEGDMMLVGDDPWEEFCSIVRKIYIYTKEEVQKMNSKSNAPRKDDSSEN
EKGSVKRDDTRGRSHGVLRERCHSAGPSQKDLGPRFIIIAIAPLNSSQLSDVNIYLRSQPPSQK

>OsIBCD005019

MASMKQQQTPASSAVTAAAAASSSATAAVAACEGERKAAAINSELWHACAGPLVSLPPVGSLVVYFPQGHSEQVYKSNI
VAASMQKDVDAHVPSYPNLPSKLICLLHGVNLHADPDTDEVYAQMTLQPVNTYGKEALQLSELALKQARPQMEFFCKTL
TASDTSTHGGFSVPRRAAEKIFPPLDFSMQPPAQELQARDIHDNVWTFRHIYRGQPKRHLLTTGWSLFVSGKRLFAGDS
VIVVRRHCDSNQISLGMRFRMMFETEELGTRRYMGTITGISDLDPVGWDESAAGERRNRVSIWEIEPVAAPFFLCPQPF
FGVKRPRQLDDESEMENLFKRAMPWLGEEVCIKDTQNQNSTAPGLSLVQWMNMNRQQSSSLANTAAQSEYLQALGNPAM
QNLAADELARQLYVQNNLLQQNCIQFNSPKLPQQMQTMNDLSKAAIPLNQLGAIINPQDQKQDAVNHQRQQNSIQVIPL
SQAQSNLVQAQVIVQNQMQQQKPSPTQNPQRINGQRLLLSHQQKDQNLQLQQQLLLQQKQQLQQQQQQQQNQQQLNKS
LGQLVNLASQQSKLFDEELQLQILQKLQQQSLMSQSTSTLSQPPLIQEQQKLITDMQKQLSNSHSLAQQQMMPQQEIKP
SLQATPLLPTVQQEQQQKLLQKQVSLADVSGVAFQPISSTNVIPKTGGAMIISGATQSVVTEEMPSCSTSPSTANGNHF
TQSTKNRHCINTERLPPSTAPMLIPTSIDAVTATPLMTKELPKPNNNVKQSVVNSKLPNVAPGPQNCINHALQTDNLET
SSSATSLCPSRTDGLVHQGFPSSNFNQHQMFKDALPDVEMEGVDPSNSCLFGINNDNLLGFPIETEDLLINALDSVKYQ
NHISTDVENNYPMQKDALQEISTSMVSQSFGQSDMAFNSIDSAINDGAFLNKNSWPAAPLLQRMRTFTKVYKRGAVGRS
IDIGRYSGYEELKHALARMFGIEGQLEDRQRIGWKLVYKDHEDDILLLGDDPWEEFVNCVRCIRILSPQEVQQMSLDGD
LGSNVLPNQACSSSDGVNGWRPRCDQNPGNPSIGPYDQFE

>OsIBCD005173

MKLSPSAGGVSDQPPSPPEVAEEQKCLNSELWHACAGPLVSLPAVGSRVVYFPQGHSEQVAASTNKEMESQIPNYPNLP
PQLICQLHNVTMHADAETDEVYAQMTLQPLSPQELKDPFLPAELGTASKQPTNYFCKTLTASDTSTHGGFSVPRRAAEK
VFPPLDFTQQPPAQELMAKDLHGNEWKFRHIFRGQPKRHLLTTGWSVFVSAKRLVAGDSVLFIWNDSNQLLLGIRRANR
PQTVMPSSVLSSDSMHIGLLAAAAHAASTNSRFTIFYNPRASPSEFVIPLAKYVKAVYHTRISVGMRFRMLFETEESSV
RRYMGTITGISDLDPVRWMNSHWRSVKVGWDESTAGERQPRVSLWEIEPLTTFPMYPSPFPLRLKRPWPTGLPSLYGGK

EDDLASSLMWLRDSQNTGFQSLNFGGLGMSPWMQPRLDSSLLGLQPDMYQTIAAAAALQNTTKQVSPAMLQFQQPQNIV
GRSSLLSSQILQQAQPQFQQMYHQNINGNSIQGHSQPEYLQQPLQHCQSFNEQKPQLQPQQQQESHQQQPQHQQMQQQ
KHLSNFQTVPNALSVFSQLSSTPQSTPSTLQTVSPFSQQHNFPDTNISCLSPSNVSSMHDTLRSFPSEAASDLPGVPRI
TPVPVSDPWSSKRVAVESTITSRPHDISSQIENFDLTPSSIPQNSTLAPLPGRECLVDQDGSSDPQNHFLFGVNIDSQS
LLMQDGIPSLHNENSSSTIPYSTSNFLSPSQDDYPLSQTLTTPGCLDESGYVPCSDNADQVKRPHATFVKVYKSGTVGR
LLDITRFSSYHELRSEVGRLFGLEGQLEDPLRSGWQLVFVDREDDVLLVGDDPWQEFVNSVSCIKILSPQEVQQMGKPG
IELFSTSARRLGNSCDNYMSRQESRSLSTGIASVGSVEF

>OsIBCD006861

MAGSVVAAAAAGGGGTGSSCDALYRELWHACAGPLVTVPRQGELVYYFPQGHMEQLEASTDQQLDQHLPLFNLPSKILC
KVVNVELRAETDSDEVYAQIMLQPEADQNELTSPKPEPHEPEKCNVHSFCKTLTASDTSTHGGFSVLRRHAEECLPPLD
MTQNPPWQELVARDLHGNEWHFRHIFRGQPRRHLLTTGWSVFVSSKRLVAGDAFIFLRGENGELRVGVRRLMRQLNNMP
SSVISSHSMHLGVLATASHAISTGTLFSVFYKPRTSQSEFVVSANKYLEAKNSKISVGMRFKMRFEGDEAPERRFSGTI
IGVGSMSTSPWANSDWRSLKVQWDEPSVVPRPDRVSPWELEPLAVSNSQPSPQPPARNKRARPPASSSIAPELPPVFGL
WKSSAESTQGFSFSGLQRTQELYPSSPNPIFSTSLNVGFSTKNEPSALSNKHFYWPMRETRADSYSASISKVPSEKKQE
PSSAGCRLFGIEISSAVEATSPLAAVSGVGQDQLAASVDAESDQLSQPSHANKSDAPAASSEPSPHETQSRQVRSCTKV
IMQGMAVGRAVDLTRLHGYDDLRCKLEEMFDIQGELSASLKKWKVVYTDDEDDMMLVGDDPWPEFCSMVKRIYIYTYEE
AKQLTPKSKLPIIGDAIKPNPNKQSPESDMPHSDLDSTAPVTDKDC

>OsIBCD007290

MITFADLAEPAPGAERCVDRQLWLACAGGMCTVPPVGAAVYYFPQGHAEHALGLAAPELSAARVPALVPCRVASVRYMA
DPDTDEVFARIRLVPLRAAEDGDVEEDGAAAGEEHEKPASFAKTLTQSDANNGGGFSVPRYCAETIFPRLDYAADPPVQ
TVVAKDVHGVAWNFRHIYRGTPRRHLLTTGWSTFVNQKKLVAGDSIVFLRGDGGDLHVGIRRAKRGFCGGGGGAEEASL
PGWDQYGGLMRGNASPCAAAKGRGKVRAEDVVEAARLASGGQPFEVVYYPRASTPEFCVRAAAVRAAMRVQWCPGMRFK
MAFETEDSSRISWFMGTVASVQVADPIRWPQSPWRLLQVTWDEPDLLQNVKRVSPWLVELVSSMPAIHLSSFSPPRKKP
RIPAYPEFPFEGQLLNPAFPPNPLAHGHHHYHHNHPSFFPFPDVSAPAGIQGARHAQFGPSLSDLHLTHLQSSLMYPGL
RRPDHVGPTSIPPPRISTDLTMGSSPPARALSMGAKKPDDAKPPGLMLFGQRILTERQMSLSGTTSPAATGNSSLNWNT
EKGASEGSGSGVIQNSPTDNTSSERLQWFRENSTVSELGLEPGQCKVFIESDTVGRNLDLSSLASFEQLYGRLSEMFCI
DSAELRSRVLYRGATGEVRHAGDEPFSRDFNKKAQSAQVSCSTVSFFIIINNDQLRVLVDSRVLRCKNPVTTHQRLECR
GPLAQGTSACSMQTQTQRCAISDLSGVLTSRLQPPGEAIPLGSLALAASSLTHDDEICDLHRAAYPWLVES

>OsIBCD014445

MEMAANPGGSGTCSDALFRELWHACAGPLVTVPKRGERVYYFPQGHMEQLEASTNQQLDQYLPMFNLPSKILCSVVNVE
LRAEADSDEVYAQIMLQPEADQSELTSLDPELQDLEKCTAHSFCKTLTASDTSTHGGFSVLRRHAEECLPQLDMSQNPP
CQELVAKDLHGTEWHFRHIFRGQPRRHLLTTGWSVFVSSKRLVAGDAFIFLRGESGELRVGVRRLMRQVNNMPSSVISS
HSMHLGVLATASHAISTGTLFSVFYKPRTSRSEFVVSVNKYLEAKKQNLSVGMRFKMRFEGDEAPERRFSGTIIGIGSV
PAMSKSPWADSDWKSLKVQWDEPSAIVCPDRVSPWELEPLDASNPQPPQPPLRNKRARPPASPSVVAELPPSFGLWKPP
SEAAQTLSFSEPQRAREIFPSIPASIFSASSHVEFNSKNEPSILSNQFYWSMRDSKTDSFSASTNKARVERKQEPTTMG
CRLFGIEISSAVEEALPAATVSGVGYDQTVLSVDVDSDQISQPSNGNKSDAPGTSSERSPLSPKVIMQGMAVGRAVDLT
KLNGYGDLRSKLEEMFDIQGDLCPTLKRWQVVYTDDEDDMMLVGDDPWDEFCSMVKRIYIYSYEEAKLLAPKSKLPVIG
DTIKLSSMNSSHESVDLDNHASVTNRDC

>OsIBCD014998

MLTFMELAGPTEGDGGGSVDSQLWAACAGSMSSVPPVGAAVYYFPQGHAEQASAAVDLSSARVPPLVPCRVVAVRFMAD
AESDEVFAKIRLVPLRPGDAVVDVGEAAAAEARREEENSRPRPTSFAKTLTQSDANNGGGFSVPRFCAETIFPELDYSS
EPPVQSVCAKDVHGVEWTFRHIYRGTPRRHLLTTGWSPFVNKKQLTAGDSIVFMRDEGGNIHVGLRRAKRGFCSIGGDD
ESLSSIPGWDQYRGLMRRNATATATGGRTPPKGKVPPENVLTAATRATTGQPFEVLYYPRASTPEFCVRAAAVRTAMAV
QWCPGMRFKMAFETEDSSRISWFMGTVAGVQASDPVRWPQSPWRLLQVTWDEPELLQNVKRVCPWLVELVSSMPNLHLP
SFSPPRKKPRNPPYAELPLEGQIFTGPVFPPNPMAHDHHHHHGFPFLPFPDSSAQPAGIQGARHAQFASPFPEFHIGNL
QPNLMLYAGIRLPPADRAAPAPRPPRIIISTDLTIGSPGKPDDAACSPSSGGKKIDDTKPRGFLLFGQAILTEEQIKNG
NSDGRPASPNWDAEKAPNTSEGSDSGVTQGSPTKNTTPSWSLPYFGGNNISRASEYELNPGQCKVFVESETVGRSLDLS
ALSSFEELYACLSDMFSIGSDELRSHLVYRSPAGEVKHAGDEPFCAFVKSARKLRILTDAGSDNLGD

>OsIBCD015915

MASSQEKAKTGVLRNAAALLDEMQLMGETQGAKKVINSELWHACAGPLVCLPQRGSLVYYFPQGHSEQVAATTRKIPNS
RIPNYPNLPSQLLCQVHNITLHADKDTDEVYAQMTLQPVNSETDVFPIPTLGAYTKSKHPTEYFCKNLTASDTSTHGGF
SVPRRAAEKLFPQLDYSMQPPNQELIVRDLHDNMWTFRHIYRGQPKRHLLTTGWSLFVGAKRLKAGDSVLFISMHIGVL
AAAAHAASSGSSFTIYYNPRTSPSPFVIPVARYNKATYMQPSVGMRFAMMFETEESSKRRYTGTVVGISDYDPMRWPNS
KWRNLQVEWDEHGYGERPERVSIWDIETPENTLVFPSSTLNSKRQCLPGYGVSVPGMEIGSANMSSFPRAQGNPYGSLQ
HIPAVGSELAIMLLNQSGQTLGSPLSFHQSSYSSIIQNVKQNYIPPLTVSTSACLTKQESLPSDDAQHQFHMANMQNGD
LEGSEVQPVIDSISESKLNATSRDPRNTDSYTSRSTSEQNSKGEPRGKTRRSKKGLPHKTVSEKSDLSSAPSWICDNQQ
VGLESKLVGCDEQVNCGNIEDSSGALTQGNFVGQPHGHQVEQKGVLSPPKVESSKSPDGGKSVNSFPNQGCFSQFIDGL
DWMTQPSYYQDSNVIQPAGVSENIFSSSADIPPSMIADTMETFQASCLSDCLPNSIQEFISSPDLNSLTFLSPDMQNLE
VQLQHDGSNLPSTSNSFVQMSFSEESASQSANLSGLHMESTHRSINTTSCSQPMSTGGFDAGMYSKLPRLKESQILSLP
EIHTNSMGTSACSMDATEYSLDRSAKPMKPPVRTYTKVQKQGSVGRSIDVTGFRNYHELRSAIACMFGLQGKLEHPGSS
EWKLVYVDYENDVLLVGDDPWEEFINCVRCIRILSPSEVQQMSENGMHVLNDCIQAA

>OsIBCD016097

MARPPAATAPPPPPPPPPPPPPPPIDRLVWLACAAPLSRIPVVGTQVSYFPEGHAEQCPAPLPDPLPSAHRFFLCTITAV
DLSADTTTGEPYATISLLPLRHDAPAPAPAAAELAEAESQEFRYYAKQLTQSDANNGGGFSVPRLCADHIFPALNLDDD
PPVQSLTMGDLQGDSWEFRHIYRGTPRRHLLTTGWSKFVNAKQLVAGDTVVFMWCGAPAPERKLLVGVRRAARYSGESA
CNARGRVQPQEVMEAVRLAAEQAAFRVTYYPRHGAGEFVVPRVEVDKGLTTPWRCGMQVRAQVMEAEDTRRLAWLNGTL
TNLRHQQIWRTLEVEWDASAASSSMKNRFVNPWQVQPVDFPPLPMGLKISNNNISAPVCNGDSLLVPPILMHPQPQPPA
DIQGARHNNGHAYADIPSSSTPSMVRTQQLFPRGLQILVPHTDIVTPQNGSPPDNPVNTPLSASDGMKTIQLFGVTITS
PVQGDTNGAFASAQVNQVPEGVDDETATEEASDTSLPDSLTNGHNQDGARL

>OsIBCD018883

MTGIDLNTVEEDEEEAAEEVAANGSSPAPARAGAVCLELWHACAGPVAPLPRKGGVVVYLPQGHLEHLGDAPAAAAAAA
AVPPHVFCRVVDVTLLADAATDEVYAQLSLVPEKEEVARRADDGEGEDGDGMKQRFARMPHMFCKTLTASDTSTHGGFS
VPRRAAEDCFPPLDYSQQRPSQELVAKDLHGTEWRFRHIYRGQPRRHLLTTGWSAFVNKKKLVSGDAVLFLRGDDGELR
LGVRRAAQLKNGSAFPALYNQCSNLGTLANVAHAVATESVFNIYYNPRLSQSEFIVPYWKFMKSLSQPFSVGLRFKMRY
ESEDASERRYTGIITGSGDTDPMWHGSKWKCLLVRWDDDAEFRRPNRVSPWEIELTSSVSGSHLSTPHSKRLKPCLPHV
NPEYMVPRGGGCPDFAESAQFHKVLQGQELLGFKSHGGTAAATSQPCEARHLQYIDERSCSSDASNSILGVPRLGDRAP
LGNPGFSYHCSGFGESQRLQKVLQGQELFRPYRGTLVDASMGSNGFHQQDSPRAPGVVNKWQAQLHGRAAFHGPPALAL
PSQSSSPPSVLMFQQANSKMPRLEFGHGQLDKHENDRRVRFGPSEGIERREQRIPLQPYPTSGEVIDGQVTVEKSHSPG
RHGKDGPDNKAVGTNSCKIFGISLTEKVPAREELDDGDANYSLQSLKQVPKSLGNSCATVHEQRPVVGRVIDISTMDMM
I

>OsIBCD019711

MKDQGSSGVSPAPGEGEKKAINSELWHACAGPLVSLPPVGSLVVYFPQGHSEQVAASMHKELDNIPGYPSLPSKLICKL
LSLTLHADSETDEVYVQMTLQPVNKYDRDAMLASELGLKQNKQPAEFFCKTLTASDTSTHGGFSVPRRAAEKIFPPLDF
TMQPPAQELIAKDLHDISWKFRHIYRGQPKRHLLTTGWSVFVSTKRLLAGDSVLFIRDEKSQLLLGIRRATRPQPALSS
SVLSSDSMHIGILAAAAHAAANSSPFTIFYNPRASPSEFVIPLAKYNKALYTQVSLGMRFRMLFETEDSGVRRYMGTIT
GIGDLDPVRWKNSHWRNLQVGWDESTASERRTRVSIWEIEPVATPFYICPPPFFRPKLPKQPGMPDDENEVESAFKRAM
PWLADDFALKDVQSALFPGLSLVQWMAMQQNPQMLTAASQTVQSPYLNSNALAMQDVMGSSNEDPTKRLNTQAQNMVLP
NLQVGSKVDHPVMSQHQQQPHQLSQQQQVQPSQQSSVVLQQHQAQLLQQNAIHLQQQQEHLQRQQSQPAQQLKAASSLH
SVEQHKLKEQTSGGQVASQAQMLNQIFPPSSSQLQQLGLPKSPTHRQGLTGLPIAGSLQQPTLTQTSQVQQAAEYQQAL
LQSQQQQQQLQLQQLSQPEVQLQLLQKIQQQNMLSQLNPQHQSQLIQQLSQKSQEILQQQILQHQFGGSDSIGQLKQSP
SQQAPLNHMTGSLTPQQLVRSHSALAESGDPSSSTAPSTSRISPINSLSRANQGSRNLTDMVATPQIDNLLQEIQSKPD
NRIKNDIQSKETVPIHNRHPVSDQLDASSATSFCLDESPREGFSFPPVCLDNNVQVDPRDNFLIAENVDALMPDALLSR
GMASGKGMCTLTSGQRDHRDVENELSSAAFSSQSFGVPDMSFKPGCSSDVAVTDAGMPSQGLWNNQTQRMRTFTKVQKR
GSVGRSIDITRYRDYDELRHDLACMFGIQGQLEDPYRMDWKLVYVDHENDILLVGDDPWEEFVGCVKSIKILSAAEVQQ
MSLDGDLGGVPPQTQACSASDDANAWRG

>OsIBCD021781

MRLSSSSGSVLPAQAASPEVGSRVVYFPQGHSEQVAASTNKEMESQIPNYPNLPPQLICQLHNVTMHADAETDEVYAQM
TLQPLSPQELKDPYLPAELGSANKQPTNYFCKTLTASDTSTHGGFSVPRRAAEKVFPPLDFTQQPPAQELIAKDLHGNE
WKFRHIFRGQPKRHLLTTGWSVFVSAKRLVAGDSVLFIWNDNNQLLLGIRRANRPQTVMPSSVLSSDSMHIGLLAAAAH
AASTNSRFTIFYNPRASPSEFVIPLSKYVKAVYHTRISVGMRFRMLFETEESSVRRYMGTITGISDLDAARWPNSHWRS
VKVGWDESTAGERQPRVSLWEIEPLTTFPMYPSPFPLRLKRPWPTGLPSLHGGKDDDLTSSLMWLRDSANPGFQSLNFG
GLGMNPWMQPRFDASLLGLQPDMYQTIAATAFQDPTKQVSPTILQFQQPQNIGGRANTLLPSQILQQVQPQFQQQQYLQ
NINETTIQGHAQSEFLQQQLQRCQSFTEQKPQLQTQQQQQESQQQQQQQSQCMQVPQHQQMQQQKNMTNYQSVPNALSP
FSQLSSPSQSSPMTLQTVLPFSQPQSYPDTSMSSLSPSNTSTMHNALRPFSSEAPSHLSMPRPTAVPVPDPWSSKRVAV
ESLLPSRPQVTSQMEQLDSTAPSIPQSSALAPLPGRGCLVDQDGNSDPQNHLLFGVNIDSQSLLMQGGIPSLQGENDST
AIPYSTSNFLSPLQNDFPLDQTLSSADCLDESGYVPCSQNSDQVINRPPATFVKVYKSGTYGRSLDITRFSSYHELRRE
LGRLFGLEGQLENPLRSGWQLVFVDREDDVLLVGDDPWQEFVNSVSCIKILSPQEVQQMGKPFELLSSAPGKRLGSSCD
DYEIEDGLPFLKDLGILLSPQQSLDDSSISLRSLALGPSICLCSSSDLGLILTTLSLPFPPSTAMEALELHNNESMLQR
SVIISNGQSPAATATLRNIQQQSDQPPYALCTGTGCRWPADGYGG

>OsIBCD021834

MRTENGDLCVGIRRAKKGGVGGPEFLPPPPPPPPPTPAAGGNYGGFSMFLRGDDDGNKMAAAARGKVRARVRPEEVVEA
ANLAVSGQPFEVVYYPRASTPEFCVKAGAVRAAMRTQWFAGMRFKMAFETEDSSRISWFMGTVSAVQVADPIRWPNSPW
RLLQVSWDEPDLLQNVKRVSPWLVELVSNMPAIHLAPFSPPRKKLCVPLYPELPIDGQFPTPMFHGNPLARGVGPMCYF
PDGTPAGIQGARHAQFGISLSDLHLNKLQSSLSPHGLHQLDHGMQPRIAAGLIIGHPAARDDISCLLTIGSPQNNKKSD
AKKAPAQLMLFGKPILTEQQISLGDAASVAVKKSSSDGNAENTVNKSNSDVSSPRSNQNGTTDNLSCGGVPLCQDNKVL
DVGLETGHCKVFMQSEDVGRTLDLSVVGSYEELYRRLADMFSIEKAELMSHVFYRDAAGALKHTGDEPFSEFTKTARRL
NILTDTSGDNLAR

>OsIBCD021943

MAAAAAAAVVGSGCEGEKTKAPAINSELWHACAGPLVSLPPAGSLVVYFPQGHSEQVAASMQKDVDAHVPSYPNLPSKL
ICLLHNVTLHADPETDEVYAQMTLQPVTSYGKEALQLSELALKQARPQTEFFCKTLTASDTSTHGGFSVPRRAAEKIFP
PLDFSMQPPAQELQARDLHDNVWTFRHIYRGQPKRHLLTTGWSLFVSGKRLFAGDSVIFVRDEKQQLLLGIRRANRQPT
NISSSVLSSDSMHIGILAAAAHAAANNSPFTIFYNPRASPTEFVIPFAKYQKAVYGNQISLGMRFRMMFETEELGTRRY
MGTITGISDLDPVRWKNSQWRNLQVGWDESAAGERRNRVSIWEIEPVAAPFFICPPPFFGAKRPRQLDDESSEMENLLK
RAMPWLGEEICIKDPQTQNTIMPGLSLVQWMNMNMQQSSSFANTAMQSEYLRSLSNPNMQNLGAADLSRQLCLQNQLLQ
QNNIQFNTPKLSQQMQPVNELAKAGIPLNQLGVSTKPQEQIHDASNLQRQQPSMNHMLPLSQAQTNLGQAQVLVQNQMQ
QQHASSTQGQQPATSQPLLLPQQQQQQQQQQQQQQQQQQQKLLQQQQQLLLQQQQQLSKMPAQLSSLANQQFQLTDQQ
LQLQLLQKLQQQQQSLLSQPAVTLAQLPLIQEQQKLLLDMQQQLSNSQTLSQQQMMPQQSTKVPSQNTPLPLPVQQEPQ
QKLLQKQAMLADTSEAAVPPTTSVNVISTTGSPLMTTGATHSVLTEEIPSCSTSPSTANGNHLLQPILGRNKHCSMINT
EKVPQSAAPMSVPSSLEAVTATPRMMKDSPKLNHNVKQSVVASKLANAGTGSQNYVNNPPPTDYLETASSATSVWLSQN
DGLLHQNFPMSNFNQPQMFKDAPPDAEIHAANTSNNALFGINGDGPLGFPIGLGTDDFLSNGIDAAKYENHISTEIDNS
YRIPKDAQQEISSSMVSQSFGASDMAFNSIDSTINDGGFLNRSSWPPAAPLKRMRTFTKVYKRGAVGRSIDMSQFSGYD
ELKHALARMFSIEGQLEERQRIGWKLVYKDHEDDILLLGDDPWEEFVGCVKCIRILSPQEVQQMSLEGCDLGNNIPPNQ
ACSSSDGGNAWRARCDQNSGNPSNGSYEQFE

>OsIBCD022554

MAQPPDAAAAAAVPPPVVIDRDVWHACAVPYSGVLPGVGTLVYYIPHGHIEQCAEDPALLLSRLPDPIHPVPCTVADLV
LDVDAESGEAYATISLLPGSHDDTTARRQVPAHGEPGFRFFEKQLSPADVTSNALVLPAGAEHVLPPLDIAAYQTARLF
DVRDLRGKRFEFVHIWDKNIWDKKRCRYMLGDLGVNDNDGWRGFVKAKRLATRDTVVFMRRGGGGDGDGDGELLVGVR
RAPRARGGHHPRPGVEDNKVVSEVWLEMQGVTPFEVTYYPREGTFEFVVSRDEYIGFSFSPFYPFVPGTTVHLRMNPLQ
IAQSISGTVRTFDHLRPWRMLEVDWDQAASPISYRIRRQVNSWQVLHNGAAEAAAADDDDGNGAGHRPRHLARLRHAVL
RPPPRRRLGGLLLPPRPRRAMPHLHDVPDPGQPPSLTLHRHRHRFAVDAQSDDSYAMISLFPGDCYVTHRPLPAARDPV
GGQREFCFFDKKLSPSDAAANGGGSGALFVIPKPSAAEHVLPRIPDLRVTNLQGGRWEFGHTWSDADTDRRSSSHTLAA
GWSAFVKAKRLCVGDTVIFMRRRPGGEPIVGVRRKPHGGMLVGIPDKHVADAWLDAVGTAEFVVRREEVEGSPPLAPGT
RVRLLMNPDDVRRRSQPPVYGTVRDVHSRSKWRMLEVDWDRDSPLAPTMNRRVNSWQVQPVQLALPPQGSDEAAAAATT
STAHAGDATTSAPSLALQLQTMASSSSSSAPIIPSRGSAFRIVNPRDGSQG

>OsIBCD027120

MASSGGGGGGGEEGEGRGATKVNQELWYACAGPLVSLPPQGSLIVYFPQGHSEQADPDTDEVYARMTLQPVSNVTQCDK
ETLLASELALKQTRPQTEFFCKTLTASDTSTHGGFSVPRRAAERIFPRLDFSMQPPAQELQARDLHDNVWTFRHIYRGQ
PKRHLLTTGWSLFVSGKRLLAGDSVLFIRDAKQQLLLGIRRANRQPTNLSSSVLSSDSMHIGILAAAAHAAANNSQFTI
YYNPRASTSEFVIPFAKYQKAVYGNQLSLGMRFRMMFETEESGTRRYMGTITGISDLDPVRWKTSHWRNIQVAWDEAAP
TERRTRVSLWEIEPIIAPFFIYPSPLFTAKRPRLPGMTDDETEMDGLLKRAMPWVGEEICKKDLNIQNSVVPGLNLAQW
MNMQHSSSLPGTVVQPELLNSLSGKPVQNLAAADLSRQISFHPQFLQQNNIQFNTALVPQQNQQTEQLAKVIPTPNQLG
SVIIPQKVVQDCNSEQRQHVVTQPVQGSQPNINIPQPQLVVQAQLQQPQVILQAQLQQPQVVVQAQLQQTQPSVQSHTV
LQGGLQQIQLLQQQQPHVQHQQIPQQLHHQQQQTQQLQPVQQVQQSVQEHQQIKIQPVHVSMDASMNTQVADHQMKLQL
LKALQPQQPLISEQQKMLLDLQQQVINSQSAPQQCVQVTNQAISLHNSNTIQYPTQQKVQSHQVQDLTGNVIPNSKSDI
ATSMGASSLHVAGGRQLLKTDDVPSTSTSPSTNSNPVLLQSIPSSSKNQSLTTAGKTSQSSVVLGPTIEQDTKPYQNVK
QTVMIPKTTEQRPATGQDCINNNPQMDYLDTSSSATSVCLSQADGSLQQNFPPSSFHQHHLLKDTVPDSEFEVTDPRNN
LLFGVNIDGQLGLPLNADLLANDIGTDKYMDQLPGNGISNFISSKDSQQELSSSMISHSFGVADMAFNSIDSAINDTPF
LNRNSRSAAGPAHQRMRTYTKVHKRGAVGRSIDINRYSGYDELKHDVARMFGIEGQLGDQNRVGWKLVYEDHEKDVLLV
GDDPWEDFVKCVRCIRILSPQEEMQMRLVGDFGDSFLPNQACSSSDGGHPWRITGD

>OsIBCD030979

MKEVGEVEEVRCLDPQLWHACAGGMVQMPAPRSRVYYFAQGHAEHADGGGGAAAAAAELGPRALPPLVLCRVEGVQFLA
DRDSDEVYAKIRLAPVAPGEAEFREPDELCPLGAAGDAAEPSPEKPTSFAKTLTQSDANNGGGFSVPRYCAETIFPKLD
YRADPPVQTVLAKDVHGVVWKFRHIYRGTPRRHLLTTGWSTFVNQKKLVAGDSIVFLRTRHGELCVGIRRAKRMACGGM
ECMSGWNAPGYGGGGFSAFLKEEESKLMKGHGGGGYMKGKGKVRMADVVEAASLASSGQPFEVAYYPRASTPDFVVKAA
SVQAAMRIQWCSGMRFKMAFETEDSSRISWFMGTISSVQVADPNRWPNSPWRLLQVTWDEPDLLQNVKCVSPWLVELVS
SIPPIHLGPFSSPRKKLRVPPHPDFPFEGHLLNPIFHGNPLGPSNSPLCCYPDTAPAGIQGARHAQFGLPLTDHQLNKL
HLGLLHSGSFNRLDAITPPSRISKGFVQITSSGSTETLSPGVTGNSAPNGNAHKTGNASDGSGSSICIGFSSQGHEASD
LGLEAGHCKVFMESEDVGRTIDLSVFGSYEELYGRLADMFGIEKEEIINHLHFHDAAGVVKHPGEVPFSDFMKAARRLT
IIAGDRERIERPLIECLVEQA

>OsIBCD033006

MATAEVGGGGGEGDAAAAAVARAGGGGGGGGGGEDALFTELWSACAGPLVTVPRVGEKVFYFPQGHIEQVEASTNQVG
EQRMQLYNLPWKILCEVMNVELKAEPDTDEVYAQLTLLPELKQQEDNGSTEEEVPSAPAAGHVRPRVHSFCKTLTASDT
STHGGFSVLRRHADECLPPLDMSRQPPTQELVAKDLHGVEWRFRHIFRGQPRRHLLQSGWSVFVSAKRLVAGDAFIFLR
GENGELRVGVRRAMRQQTNVPSSVISSHSMHLGVLATAWHAVNTGTMFTVYYKPRTSPAEFVVPYDRYMESLKRNYSIG
MRFKMRFEGEEAPEQRFTGTIVGMGDSDPAGWPESKWRSLKVRWDEASSIPRPERVSPWQIEPAVSPPPVNPLPVPRTK
RLRPNATALPADSSAIAKEAATKVVVESEPNGTQRTFQTQENATPKSGFGNSSELESAQKSIMRPSGFDREKNNTPIQW
KLGSDGWMQMSKPESYSEMLSGFQPPKDVQTPQGFCSLPEQITAGHSNFWHTVNAQYQDQQSNHNMFPSSWSFMPPNTR
LGLNKQNYSMIQEAGVLSQRPGNTKFGNGVYAALPGRGTEQYSGGWFGHMMPNSHMDDTQPRLIKPKPLVVAHGDVQKA
KGASCKLFGIHLDSPAKSEPLKSPSSVVYDGTPQTPGATEWRRPDVTEVEKCSDPSKAMKPLDTPQPDSVPEKPSSQQA
SRNMSCKSQGVSTRSCKKVHKQGIALGRSVDLTKFNGYEELIAELDDMFDFNGELKGPKKEWMVVYTDNEGDMMLVGDD
PWIEFCDMVHKIFIYTREEVQRMNPGTLNSRSEDSHANSMERGSVGREMRGCLSTSSLNSENC

>OsIBCD034941

MSMSMFVSSQSKILRAAWKSQPPRKAQQQQAAAESPEATNHIAPSTASVEASTNQVAEQQGAPLYNLPWKIPCKVMNVE
LKAEPDTDEVYAQLTLLPEKVEEEEVVPPAATERPRVHSFCKTLTASDTSTHGGFSVLRRHADECLPPLYSFFDRLIVA
MPLTSLLDQDMSQHPPTQELVAKDLHGVEWRFRHIFRGQPRRHLLQSGWSVFVSAKRLVAGDAFIFLRGENGELRVGVR
RAMRQQANIPSSVISSHSMHLGVLATAWHAVNTGTMFTVYYKPRTSPSEFVVPRDLYKESLKRNHSIGMRFKMTFEGEE
AAEQRFTGTIVGVGDSDPSGWADSKWRSLKVRWDEAASVPRPDRVSPWQIEPANSPSPVNPLPAPRTKRARPNVLASSP
DLSAVNKEVASKVMANSQQNGLPRAFHSQENMNLRSRFGDSNELNTSQKLTMWSSGSNQEKNNVSVQRELGSQSWMQMR
SPDGSSEILSGFQPLKDTRNPLSSFPSQISGNRSNTWNTINVHYPDQNANHNMYPGTWSLMPPNTGFGVNQQNYLMTPD
ITLPQRSLNAKFGGNGAFTSLRAHGIDQRSSGWLGHIEPSSHIDDASSSLIKPQPLVIDHNVQKAKGSSCMLFGISLDS
PAKPELLISPPSVAFDGKLQQDALEEDECSDPSKTVKPLDGAQHDSATEKHQSCPDGTKNIQSKQQNGSSRSCKKVHKQ
GIALGRSIDLTKFTCYDELIAELDQMFDFNGELNSSSKNWMVVYTDNEGDMMLVGDDPWNEFCNMVHKIFIYTREEVQK
MNPGALNSRSEDSRSTSVERGLVGEGLRGGLSTPSLNSENC

>OsIBCD035656

MKLSPPASADMPQALPENDGEQRCLNSELWHACAGPLVSLPVVRSRVVYFPQGHSEQVAASTNKEVDAQIPNYPNLPPQ
LICQLHNVTMHADAETDEVYAQMTLQPLSPEEQKEPFLPMELGAASKQPTNYFCKTLTASDTSTHGGFSVPRRAAEKVF
PPLDFSQQPPAQELIARDLHDNEWKFRHIFRGQPKRHLLTTGWSVFVSAKRLVAGDSVIFIWNDNNQLLLGIRRANRQQ
TVMPSSVLSSDSMHIGLLAAAAHAAATNSRFTIFYNPRASPSEFVIPLAKYVKAVYHTRVSVGMRFRMLFETEESSVRR
YMGTITSISDLDSVRWPNSHWRSVKVGWDESTTGDKQPRVSLWEIEPLTTFPMYPSAFPLRLKRPWASGLPMHGMFNGG
GNDDFARYSSLMWLRDGNRGTQSLNFQGHGVSPWLQPRIDSPLLGLKPDTYQQMAAAALEEIRYGDPSKQHPATLQYQQ
THNLNSGLNSLFASHVLGQVQFQPQQSPLQVVQQGHCQNTGDSGFLQGQLPRLQLHNTQQLLKEQELQQQQRQHVLQEQ
SSQEMQQQLPSSDHRVADVASESGSAPQAQSSLLSGSSFYNQNLLEGNSDPPLHLHNNFHNFSNQEPSNLLSLPRSSQL
MASDGWPSKRLALESAVHPEAPSMHPKIEKVNHQGISHFPGAFPPQSARGCSIVQDCRADAENRLLSSSFELQDGMTSI
ITDANRETDTMAIPLLRYSGADLTTENTLATSNCLGESGTFNPLNNISVNPSQGATFVKVYKSGSLGRSLDISRFSSYC
ELRSELERLFGLEGQLEDPVRSGWQLVFVDRENDILLVGDDPWQEFANSVWCIKILSPQEVQQLVRGGDGLLSSPGARM
QQSNACDDYSASHNMQNIAGNIASVAPLDY

4. *S. lycoperiscum* ARFs

>Solyc03g118290

MAASEVSIQGYSEPSDGSRPVSETGRSSSGVGIVDADTALYTELWRSCAGPLVTVPREGELVYYFPQGHIEQVEASTNQ
VADQQMPLYNLPSKILCRVVNVLLKAEPDTDEVYAQVTLMPEPNQDENAVKKEPMRPPPPRFHVHSFCKTLTASDTSTH
GGFSVLRRHADECLPQLDMSRQPPTQELVAKDLHGNEWRFRHIFRGQPRRHLLQSGWSVFVSSKRLVAGDAFIFLRGEN
GELRVGVRRAMRQQGNAPSSVISSHSMHLGVLATAWHAIQTKTMFTVYYKPRTSPAEFIVPYDHYMESVKNNYSIGMRF
KMRFEGEEAPEQRFTGTIVGIEDADPQRWLESKWRCLKVRWDENSSIPRPDRVSPWKIEPALSPPALNVPPVARPKRPR
SSILPTSPDSSVLTREGSSRATADHSQASGFPRVLQGQELSTFRGGFAEINETDLSEKPMIWQTSVNDEKNDIHSASKR
YLPDKWLPLGRPESSLTDLLSGFGSSHGFCLPSADQAAFGARLVKQQTQDQEKDFSLLGKPWSLLSSGLSLNLMDSGSK
APGIGGDTPYQMRGDARYSGYGEFSVLPGHRVANQQGSWIMPQPVSPYMQLSSHSREMMHKPSVVKQPEAVKPKEGNYK
LFGIPLTSNVCTDAVMMRKSSLIDPASDMNIGIHPHQSLATDSDQRSEQSKGSKVDDGVAANDHDKQFHTFHLAARDKD
GKGHSSSTRSCTKVHKQGTALGRSVDLAKFNNYDELIAELDQLFDFNGELKARSKSWLVVYTDDEGDMMLVGDDPWQEF
CGMVRKIFIYTKEEVQRMNPGTLNSKGEDTSSVAEGSDAKEVKNLQLPSESGQAES

>Solyc12g042070

MATSENCRNAAGAGKVDAEKALYTELWRACAGPLVTVPCEGELVFYFPQGHIEQVEASTNQASDQQMPVYNLPSKILCR
VINVLLKAEPDTDEVYAQVTLLPEPNQDENVVSKEPMPSPPPRFHVHSFCKTLTASDTSTHGGFSVLRRHADECLPPLD
MSRQPPTQELVAKDLHANEWRFRHIFRGQPRRHLLQSGWSVFVSSKRLVAGDAFIFLRGENGELRVGVRRAMRQQGNAP
SSVISSHSMHLGVLATAWHAIQTKTLFTVYYKPRTSPADFIVPYDQYMESLKNNYSIGMRFKMRFEGEEAPEQRFTGTI
VGIENADLKRWPESKWRCLKVRWDETSAIPRPDRVSPWKVEPALSPPALNPLPIPRQKRPRSNVLPSSPDSSVLTREGS
SKVVVDTSQASGFSRVLQGQEISTLRGNFVENNESDSSEKPPIWQPLLDDEKADVHSASRKCISDKRLPLGRPESSFTD
LLSGFGGQSSSSHGFHSPTGGQTAPASWVKRQALDKETDFSLLAKQWSLVSSGLSLNLMESGLKGADTLYQMRGTSRLN
CFNEYPTFPGHRPDNQQGNWLMPPSVLPYIQMSAHSGEIMPKPMASPQPEAMKPKEGNCKLFGIPLVSKCATIDPVMLR
KNSPIHSTSNMHFGIHPHQFPIIESDQRSEQSKGSKLPDDGFIVHDQEEQFQTSHPGTRDREGKGLVHSTRSCTKVHKQ
GTALGRSVDLAKFNNYEELIAELDHIFDFNGELKARNKNWLVVYTDDEGDMMLVGDDPWEFCGMVRKIFIYTKDEVQRM
NPGTLNSKGEDNSSVAEGSDAKEVKNLQLHIDSSPEDS

>Solyc01g103050

MAHVAANHFGGGTHPGASANNALYKELWHACAGPLVTVPREGERVYYFPQGHMEQLEASTHQGVDQHLPSFNLPAKILC
KVMNVQLRAESETDEVYAQITLLPEPDQGEITSPDPPLPEPEKCTVHSFCKTLTASDTSTHGGFSVLRRHADECLPPLD
MSQQPPWQELVASDLHGNEWHFRHIFRGQPRRHLLTTGWSVFVSAKKLVAGDAFIFLRGDSGELRVGVRRLMRQLNNMP
SSVISSHSMHLGVLATASHAIMTGTLFSVFYKPRTSQSEFIVSVNKYLEARNHKLSVGMRFKMRFEGEEVPERRFSGTI
VGVGDNPSSRWPDSEWRSLKVHWDEPSSILRPDRVSPWDMEPLVAATPTNTQPPQRNKRARPSVLPSPVQELPALGMWK
SPVDSPSSFSYCDPSRGRDLYPSPKLSSAAKGLGYGENGSMPLSTKTMYWSSQSETCTESVAPASEKRPANGCRLFGIE
LLDCPTIDESSSVAMPSAVVEDQPVPSLNVDSDRNSEPSNPIPSVSCEPEKSSLRSTHESQSKQIRSCTKVHMQGKAVG
RAVDLTRLDSYEDLLKKLEVMFEIEGELRGSTKKWQVVYTDDEDDMMMVGDDPWHEFCSMVRKIYVYTAEEAKKLSPKI
KLPVVDVKPVSDGATVGNNEKA

>Solyc08g008380

MENQGSFMSNQQHQQHNFLAEGEDELCQELWRLCAGPLVDVPKNEERVYYFPQGHMEQLEASTNQELNQSIPLFNLQPK
ILCRVLHIQLLAEQDSDEVYAQIALLPEADQVEPTSPDLSLPEPPRPKVHFFCKVLTASDTSTHGGFSILRKHANECLP
PLDMTQATPAQELVAKDLHGFEWHFKHIFRGQPRRHLLTTGWSTFVSSKRLVTGDSFVFLRSGKGEVRIGIRRLARQPS
SMPQSVISSQSMHLGVLATASHAVTTQTMFVVYYKPRTSQFIIGLNKYLEAVKHRYSVGMRFKMKFEGEEIPEKRFTGT
IVGVEDSSSQWKDSKWRSLKVQWDEPASVPRPDRVSPWDIEPFVASVATPLVPPMGVKNKRHRAHNEPKSSEPVPAAAL
AAWIPSAQFNPVIEGQSSDNPFSLHTSQTNSTATNSTFKARVDGIWSASKVNASLNMLLDETEASKSASPRPAFPSFAS
SQFGKQNDLLLPSLDDERKCGTITSCRLFGIDLKCPSFGSVNENPPLEPANNSDGSAEGCSGNLTSAGDSEDNSGLSRD
SEDQKQEQLNPPPKEVHIKQVSSTRTRTKVQMQGVAVGRAVDLTKLNGYDELLRELEELFDIQEELHARNKWEIVFTDD
EGDMMLMGDYPWSEFCNIAKRIFICSSQDMKSFSAGTKSPSCLESETTAST

>Solyc01g096070

MNLGMGTEDLYRDLWKACAGPLVDVPREGERVYYFPQGHIEQLEASTNQAVNQQIPQFNLASKILCHVFHVQLLAETET
DEVYAQITLHPEAEQEEPSKPDPCPPDLPKRTVHSFCKILTASDTSTHGGFSVLRKHANECLPQLDMTQATPTQDLVAK
DLHGYEWRFKHIFRGQPRRHLLTTGWSTFVTSKRLVAGDAFVFLRDDSGELRVGVRRLARQQSPIPQSVISSQSMHLGV
LATASHAITTQTRFVVYYKPRTSQFIVGLNKYLEAVSHGFSVGMRFRMRFEGEDSPERRFTGTIVGTGDISSQWSESKW
RSLKIQWDEPASMVRPDRVSPWEIEPFVASTCVDVAQPGIKSKRPRPLDLPRSEIAVASAASPFWCPGSGLTLEVSHLG
GITEVQSRDNQLFWSSKQSSSLSNGMSNTSCRTHLSGAWQHNMLANGSLNLLRDSIEDNKQLITRSALLDYGSPMSSRV
SSGLLHDQVNRGSKHVISSACRLFGIDLRNNSNNTPSKAKEMLGPNITSNCADEAPVVHDESEVDKDQNVGHLNPSEEK
KQFQLEALPKDTQKQGPTSSRTRTKVQMEGVCVGRAVDLTALSGYDDLISELEKIFDIKGELCPRNKWEVVYTDDEGDM
MLVGDDPWLEFCKMVRRIFIYSSEEVKKMTPRCKLPILSLEGEGTMPSVDSELKAEG

>Solyc08g082630

MNSPGKKDALYHELWQLCAGPVVDVPREGERVYYFPQGHMEQLVASINQEMDQRVPSFNLKSKVLCRVINSHFLAEEDN
DEVYVQITLMPEAPHVPEPTTPDPLIPQDVKPRFHSFCKVLTASDTSTHGGFSVLRKHANECLPPLDLNQQTPTQELIA
KDLHDVEWRFKHIFRGQPRRHLLTTGWSTFVSSKKLVAGDSFVFLRGNNGQLRVGVKRLVRQQSSMPSSVMSSQSMHLG
VLATASHAVTTQTMFVVYYKPRTTQFIVGVNKYLEALKHEYAVGMRFKMQFEAEGNPDRRFMGTIVGIDDLSSQWKNSA
WRSLKVRWDEPAAIARPDRVSPWEIKPYVCSIPNVLVPPTAEKNKRHRLHSEIKISEQPSSSNASAVWNPSLRSPQFNT
FGINSSTNCALASLTESGWQLPHLNTSGMLVDEPEDGRSAPTWCGFPCVLAPQFGQGTNQPIVIPTDGRKCDTKKTCRL
FGIDLKSSSISTTEARLQLQPAGISCVFAERAPPNTVPAGDSDQKSELSVDFKDQMQGHLRLPLKEVQSKQSCSTRSRT
KVQMQGVAVGRAVDLTILKGYDELTKELEEMFEIQGELQSRQKWGILFTDDEGDTMLMGDYPWQDFCNVVRKIFICSSQ
DMKKLTLSRADS

>Solyc07g043620

MRVSSSGFNPQPEEAAGEKKCLNSELWHACAGPLVSLPPVGSRVVYFPQGHSVQVAASTNKEVDAHIPNYPGLPPQLIC
QLHNLTMHADVETDEVYAQMTLQPLSPQEQKDVCLLPAELGIPSKQPTNYFCKTLTASDTSTHGGFSVPRRAAEKVFPP
LDYSQQPPCQELIAKDLHGNEWKLRHIFRGQPKRHLLTTGWSVFVSAKRLVAGDAVIFIWNENNQLLLGIRRANRPQTL
MPSSVLSSDSMHIGLLAAAAHATATNTRFTIFYNPRASPSEFVIPLAKYAKAVYHTRISVGMRFRMLFETEESSVRRYM
GTITGISDLDPVCWPNSHWRSVKVGWDESTAGERQPRVSLWEIEPLTTFPMYPSPFSLRLKRPWPSGLPSLPGFPNVGL
TMNSPLSWLRGDMGDQGMQSLNFQGFGATPFMQPRMDASMLGLQPDILQTMTALDPSKLANQSLMQFQHSIPNSSAPLS
QIQMLQPSHSQHNLIQGFSENHLISQAQMLQQQLQRRQNFNDQQQLLQPQL

>Solyc03g031970

MKLSTSGMGQQAHEGENKCLNSELWHACAGPLVCLPTVGSRVVYFPQGHSEQVAATTNKEVDIHIPNYPNLPPQLICQL
HNVTMHADVETDEVYAQMTLQPLTLQEQKDTYLPVELGIPSRQPTNYFCKTLTASDTSTHGGFSVPRRAAEKVFPPLDF
SQTPPCQELIARDLHDIEWKFRHIFRGQPKRHLLTTGWSVFVSAKRLVAGDSVLFIWNEKNQLFLGIRRATRPQTVMPS
SVLSSDSMHIGLLAAAAHAASTNSCFIVFFNPRASPSEFVIPLSKYIKAVYHTRVSVGMRFRMLFETEESSVRRYMGTI
TGIGDLDPVRWANSHWRSVKVGWDESTAGERQPRVSLWEIEPLTTFPMYPSLFPLRLKRPWYPGTSSFQENNSEAINGM
TWLRGESSEQGPHLLNLQSFGGMFPWMQQRVDPTMLRNDLNQQYQAMLASGLQNFGSGDLMKQQLMQFPQPVQYVQHAG
SVNPQLQQQQQQQETMQQTIHHHMLPAQTQDNLQRQQQQHVSNQTEEQSHQHSYQDAYQIPNSQLQQKQPSNVPSPSFS
KPDIADPSSKFSASIAPSGMPTALGSLCSEGTTNFLNFNIIGQQPVIMEQQQQQKSWMAKFANSQLNMGSSSPSLSGYG

KETSNSQETCSLDAQNQSLFGANVDSSGLLLPTTVSNVATTSIDADISSMPLGTSGFPNPLYSYVQDSTDLLHNVGQAD
AQTVPRTFVKVYKSASLGRSLDITRFNSYHELRQELGQMFGIEGFLENPQRSGWQLVFVDRENDVLLLGDDPWEEFVNN
VWYIKILSPEDVQKLGKEEVGSLNRGPPERMSSNNSADGRDFMSGLPSIGSLDY

>Solyc04g081240

MGSVEEKNKPGSLVSGAHTLLEEMKLLKEMQDHTGGGRKLISSELWHACAGPLVTLPQVGSLVYYFPQGHSEQVAVSTN
RTATSQIPNYPNLASQLLCQVHNVTLHADKETDEIYAQMSLQPVNSEKDVFPIPDFGLKPNKHPTEFFCKTLTASDTST
HGGFSVPRRAAEKLFPPLDYSMQPPTQELVVRDLHDNTWTFRHIYRGQPKRHLLTTGWSMFVGAKRLRAGDSVLFIRDE
KSQLLLGVRRANRQQTSLPSSVLSADSMHIGVLAAAAHAAANRSTFTIFYNPRACPSEFVIPLAKYRKSIYNTQLSVGM
RFGMMFETEESGKRRYMGTISGISDLDPLRWPSSKWRCLQVEWDEPGCGDKQNRVSPWEVETPESLFIFPSLTAGLKRP
YQSTFLGAQTEWDSLMQHRPFMRVPENVYGDLQSSSISNLWSEQLMKMLIRPPPGLTGLQCGVPTVQDIKVALPQEARN
VVQPAGNQKPELITVEATPAQSETNSEVVLNQPVGVVNSISSQQATLQAKSKPPEKVETDIIGKNSEPRKETSNSSVKL
DQFQCNEDKVAIKPASPHDLPTDASVTASHHNSFSQLQASPWLIPHNPQIDSAASNNILQCPTNNEWNMSSLQSAAGLL
KYPVSTSTLTKHDNSFMLPDTIGHGLAPIGQDLWDHQLNDVKCFSQTNLQVPLDITNMQFLPDSYGFKDLSEESHNQSD
IYSCLNFDSNSGSTVIDNSVSSTVLDEFCNLKHTDFQNPSDFLLGNISSSQDVQSQITSASLADSQNFSVQEFADNSGG
ASSSNVNFDECNLLQNSSWQQVAPRVRTYTKIQKTGSVGRSIDVSGFKNYEELRSEIERMFGLEGLLNDTRGSSWKLVY
VDFENDVLLVGDDPWEEFVGCVRCIRILSPTEVQQMGEEGMQLLNSAGLQSINGSTSEFPN

>Solyc03g031970

MKLSTSGMGQQAHEGENKCLNSELWHACAGPLVCLPTVGSRVVYFPQGHSEQVAATTNKEVDIHIPNYPNLPPQLICQL
HNVTMHADVETDEVYAQMTLQPLTLQEQKDTYLPVELGIPSRQPTNYFCKTLTASDTSTHGGFSVPRRAAEKVFPPLDF
SQTPPCQELIARDLHDIEWKFRHIFRGQPKRHLLTTGWSVFVSAKRLVAGDSVLFIWNEKNQLFLGIRRATRPQTVMPS
SVLSSDSMHIGLLAAAAHAASTNSCFIVFFNPRASPSEFVIPLSKYIKAVYHTRVSVGMRFRMLFETEESSVRRYMGTI
TGIGDLDPVRWANSHWRSVKVGWDESTAGERQPRVSLWEIEPLTTFPMYPSLFPLRLKRPWYPGTSSFQENNSEAINGM
TWLRGESSEQGPHLLNLQSFGGMFPWMQQRVDPTMLRNDLNQQYQAMLASGLQNFGSGDLMKQQLMQFPQPVQYVQHAG
SVNPQLQQQQQQQETMQQTIHHHMLPAQTQDNLQRQQQQHVSNQTEEQSHQHSYQDAYQIPNSQLQQKQPSNVPSPSFS
KPDIADPSSKFSASIAPSGMPTALGSLCSEGTTNFLNFNIIGQQPVIMEQQQQQKSWMAKFANSQLNMGSSSPSLSGYG
KETSNSQETCSLDAQNQSLFGANVDSSGLLLPTTVSNVATTSIDADISSMPLGTSGFPNPLYSYVQDSTDLLHNVGQAD
AQTVPRTFVKVYKSASLGRSLDITRFNSYHELRQELGQMFGIEGFLENPQRSGWQLVFVDRENDVLLLGDDPWEEFVNN
VWYIKILSPEDVQKLGKEEVGSLNRGPPERMSSNNSADGRDFMSGLPSIGSLDY

>Solyc04g081240

MGSVEEKNKPGSLVSGAHTLLEEMKLLKEMQDHTGGGRKLISSELWHACAGPLVTLPQVGSLVYYFPQGHSEQVAVSTN
RTATSQIPNYPNLASQLLCQVHNVTLHADKETDEIYAQMSLQPVNSEKDVFPIPDFGLKPNKHPTEFFCKTLTASDTST
HGGFSVPRRAAEKLFPPLDYSMQPPTQELVVRDLHDNTWTFRHIYRGQPKRHLLTTGWSMFVGAKRLRAGDSVLFIRDE
KSQLLLGVRRANRQQTSLPSSVLSADSMHIGVLAAAAHAAANRSTFTIFYNPRACPSEFVIPLAKYRKSIYNTQLSVGM
RFGMMFETEESGKRRYMGTISGISDLDPLRWPSSKWRCLQVEWDEPGCGDKQNRVSPWEVETPESLFIFPSLTAGLKRP
YQSTFLGAQTEWDSLMQHRPFMRVPENVYGDLQSSSISNLWSEQLMKMLIRPPPGLTGLQCGVPTVQDIKVALPQEARN
VVQPAGNQKPELITVEATPAQSETNSEVVLNQPVGVVNSISSQQATLQAKSKPPEKVETDIIGKNSEPRKETSNSSVKL
DQFQCNEDKVAIKPASPHDLPTDASVTASHHNSFSQLQASPWLIPHNPQIDSAASNNILQCPTNNEWNMSSLQSAAGLL
KYPVSTSTLTKHDNSFMLPDTIGHGLAPIGQDLWDHQLNDVKCFSQTNLQVPLDITNMQFLPDSYGFKDLSEESHNQSD
IYSCLNFDSNSGSTVIDNSVSSTVLDEFCNLKHTDFQNPSDFLLGNISSSQDVQSQITSASLADSQNFSVQEFADNSGG
ASSSNVNFDECNLLQNSSWQQVAPRVRTYTKIQKTGSVGRSIDVSGFKNYEELRSEIERMFGLEGLLNDTRGSSWKLVY
VDFENDVLLVGDDPWEEFVGCVRCIRILSPTEVQQMGEEGMQLLNSAGLQSINGSTSEFPN

>Solyc02g037530

MKLSTSGMGQQAHEGGEKKCLNSELWHACAGPLVCLPTVGSRVVYFPQGHSEQVAATTNKEVDAHIPNYPNLSPQLICQ
LHNVTMHADVETDEVYAQMTLQPLTPEEQKDTYLPVEFGIPSKQPTNYFCKTLTASDTSTHGGFSVPRRAAEKVFPPLD
FSQTPPAQELIARDLHDVEWKFRHIFRGQPKRHLLTTGWSVFVSAKRLVAGDSVLFIWNEKNQLLLGIRRAVRPQTVMP
SSVLSSDSMHIGLLAAAAHAAATNSCFNVFFNPRASPSEFVIPLSKYIKAVYHTRVSVGMRFRMLFETEESSVRRYMGT
ITGIGDLDPVRWANSHWRSVKVGWDESTAGERQPRVSLWEIEPLTTFPMYPSLFPLRLKRPFYQGTSSYQDSNNEAINR
MSWLRGNAGELGHHSMNLQSFGMLPWMQQRVDSTILPNDINQHYQAMLATGLQSFGSGDLLKQQLMQFQQPVQYLQHAS

TENSILHQQQQQQQQIMQQAVHQHMLPAQTQMLSENLQRQSQHQSNNQSEEQAHQHTYQEAFQLPHDQLQQRQPSNVTS
PFLKADFADLTSKFSASVAPSGVQNMLGSLCSEGSNNSLNINRTGQSVIIEQSPQQSWMSKFTESQLNTCSNSSSLPTY
GKDTSNPRGNCSLDSQNQALFGANIDSSGHLLPTTVSNVTTTCADMSLMPLGASGYQNSLYGYVQDSSELLHNAGQIDP
PNATHTFVKVYKSGCVGRSLDITQFHSYHELRRELGQMFGIEGFLEDPQRSGWQLVFVDRENDILLLGDDPWEAFVNNV
WYIKILSPEDVQKLGKEEAESLNRGAVERMSSTNADDRDLISGMPSLGSLEY

>Solyc07g042260

MKAPSNGYLPNSGEGERKLMNSELWHACAGPLVSLPPVGSLVVYFPQGHSEQVAASMQKETDGIPSYPNLPSKLICMLH
NVTLHADTETDEVYAQMTLQPVNKYDQEALLLSEMGLKQNRQPAEFFCKTLTASDTSTHGGFSVPRRAAEKIFPPLDYA
MQPPAQELMARDLHDQTWTFRHIYRGQPKRHLLTTGWSVFISSKRLCAGDSVLFIRDEKSQLLLGIKRTNRQQPALSSS
VISSDSMHIGILAAAAHAAANNSPFTIFYNPRASPSEFVIPLAKYNKAMYAQVSLGMRFRMMFETEESGVRRYMGTITG
VSDLDPIRWKSSQWRNLQVGWDESTAGERPSRVSIWDIEPVVTPFYICPPPFFRPKFPKQPSFPGDESDIENVLKRGMP
WINDELGLKDAQNSIFPGLSLVQWMSMQQNNHVPVAQSGLPSVLHSNIGSDDHSKLLNFQSPALATPGLQFNKPNQLNQ
PFGQIQQPPLAWTQEQQQSLQSPVSAQQQQQQQHTLQQQQQQQHTLQQQQQQRHTLQQQQQQTLQQQQQQQQQHTL
QQQQQQQHTLQHQQQHTLQQPQQHMLQQQPQFHQQAQQQLQQQQRPSQQQQLTGNSSPVNRCVSPNQIPNQTFPQAAVY
GQLQQQQVLSASTQSQQNVPVSRNSFPSTSLAQDFQFQQQMEQQSNLLQKSQQQQTIPQQAPLQLLQQSLMQRSQVQPS
SQQSLTEQQLQLQLLNKLQQQQQQQQAQLLSPVSSTLEPRMPQQQQNRQPQELQFAHQQLSSNIVTTVTHLQSTHHAFN
QLQNHHKSPITIKALSGGTEGDAPSCSTSPSTNNFQVSPPNFLTRNQGQAILVDESVVDPSQEQNKSECRIKHELVFSK
GSELSKYKGNNTENLEAASSTTSYGLDSSGFNFSLPALCVDGDVQSHSRNILPSAANNIDGLNPDALLSRDYDSGKDIQ
NLFSPFGNAPRDIETELSDAGINSQQFGVPNMSYKPRCANDLAVNDNGILNNNAWTNQTQRMRTYTKVQKRGSVGRTID
VTRYIGYDELRHDLARMFGIEGQLEDPQRTEWKLVYVDHENDILLVGDDPWEEFVSCVQSIKILSCAEVQQMSLNGDLG
NVPVPNQASSGTDSGNAWKGHYDDNSAASFN

>Solyc02g077560

MMCGLIDLNTVDNDDAGEETTAPVSLDSPASSSAASGSSDLTSSTTPAVASVCMELWHACAGPLISLPKKGSAVVYLPQ
GHLEHLSEYPSIACNLPPHVFCRVVDVKLQADAATDEVYAQVSLVPDNQQIEQKWKDGDIDADIEEEEIEGAGKSITPH
MFCKTLTASDTSTHGGFSVPRRAAEDCFAPLDYRQQRPSQELVAKDLHGIEWKFRHIYRGQPRRHLLTTGWSAFVNKKK
LVSGDAVLFLRTGDGELRLGVRRAAQAKTCSSYLAPCSKPLNVSGIVDAVNVISSRNAFNICYNPRDSSSDFIVPYHKF
SKTLAHPFSAGMRFKMRVETEDAAEQRFTGLVVGVSNVDPVRWPGSKWRCLLVRWDDLDVSRHNRVSPWEIEPSGSAPV
PSSLVMPSAKRTRVGFPISKADFPIPREGIAVSDFGEPSRFQKVLQGQEILRMHAPYGGLDARSPRPAGTRCFPGFPSS
GISRMGNSIRPLFGDTDKSHESIGFSESLRFNKVLQGQEIFTSPPYGRAQAGIQMQEKSRTGIFVGIQVPNHGNRWPAP
NQDNNTPCKPINPVSASSPPSALNFQHPSPPASKFQAMFNHKHDLVNQASLDLSENCCRYPYLSSGSHTEDISQKEGTQ
GISSFGFLKEQKQTGLSYLSLGTQSSFKGNQNLVSTCKTGCRIFGFPLTESKISATRADTPSEAVYSHGLETTFLPSSD
GKLQPGPPLMTNVVGTNFTKVNDLYAARDVLLDIAL

>Solyc11g069190

MEIDLNHALVSEVEKNVCCNEECDKGGGGGCVNCSLYTSTTSSCSSNVSSSSSLALTSIYKELWHACAGPLTSLPKKGN
VVVYFPQGHMEEAVSAFPFSPVKIDLPTFGLQPQIFCRVEDVQLLANKENDEVYTQLTLLPLPESMAISLEGKEHEDSG
TDEEGNGVNPGKSASHMFCKTLTASDTTTHGGFSVPRRAAEDCFPPLDYKEQRPSQELIAKDLHGVEWKFRHIYRGQPR
RHLLTTGWSIFVSQKNLVSGDAVLFLRGEGGNLRLGIRRAARPRNGLPESIIKSQYSGPDVLSSVATALSAKSTFHVFY
SPRASHADFVVPYQKYVKAINSRIPVGTRFKMKFDLDDSPERRYSGVVTGISDMDPFRWPNSKWRCLMVRWDEDIMSNH
QERVSPWEIDSSVSLPPLSIQSSPRLKKLRTSQQAPSVLDSHFAGGSALLDFEESIRSSKVLQGQENLGLISPPYGCDK
PVRPLDFELQRVARHNLMPNGVENIIVGDFVKTQPPTTYTGFLESNRFPKVLQGQEICSLRSLTGKGDVNFGAWGKPEF
GCNVFGTYQRPRANFYPLASEGARNVFLPYNAMYRAGQDPVVPSYSTNFQRENPTLNQNSIQNVVRREEVGMPKFVNEQ
RPPEMSKVSIPENHFKNENDDSFNAQAPCKLFGFSLTKEPSTPSSQSSGKRSCTKVHKQGSLVGRAIDLSRLNGYDDLL
VELERLFNMEDLLRDPNKGWRILYTDSENDMMVVGDDPWHEFCEVVSKIHIYTQEEVEKMTIEGISDDTQSCLEEAPAI
MDVSKSSSSVGQPDSSPTVIRI

>Solyc07g016180

MKTPVNTAGVQQQHTVNGNPGEVEKKSINPELWQACAGPLVNLPAAGTHVVYFPQGHSEQVAASMKKDVDAQIPNYPNL
PSKLVCLLHNITLHADPETDEVYAQMTLQPVPSFDKEALLRSDLSMKANKPQTEFFCKTLTASDTSTHGGFSVPRRSAE
KIFPPLDYSMQPPAQELVARDLHDNLWTFRHIYRGQPKRHLLTTGWSLFVSGKRLFAGDSVLFIRDEKQQLLLGIRRAN

RQPTNLSSSVLSSDSMHIGILAAAAHAAANNSPFTVFYNPRASPSEFVIPLAKYYKATYSSQVSLGMRFRMMFETEESG
TRRYMGTITGISDLDPVRWKNSQWRNLQVGWDESTAGERRNRVSIWEIEPVTAPFFICPTPPFFRSKRPRLPGMPDDDC
SDLDGLFKRTMPWLGDDFGMKDPQGLPGLSLVQWMNMQQNPSLANSMQPNYLHSLSGSVLQNVGGGADLSRQLCLPAPQ
LPQQNTLQFGSQRPTQQVQQLDQLQKIPTTTLSPAGSIMQPQQQLSDISQQPRQNLINQSVPTNHVQAQLLQAQSLVQS
QNVLQQQQSFQNQLQRNLPQNLPQQQQIMNQTQQQSFMQPQPSDPLNQQLHFSDNQLQMQLLQKLQQQSLLAQQSLLQQ
PSQLMPIQDQQKHLDVSQNFSRSLATSQMLDMSQTTSNSTSLSQPQVAQQQMTINNSQSNLRFAQPNQHMKQQQQQQP
GILPEIPGQVGQILPPTTNQLSANCSSFLTGAVGGGQSVVTDDIPSCSTSPSTNNCQNVVQPIMNGRIHRGTAAAEETT
QSSLPLLSSSGLEAMSPNRNLVKDLQQKPDVKPSMNISKSQNHGFSTPQTYLNNAVPQMDYLDSSSSATSVYFSQNDVQ
LQQTTNPMSFSSQAIVFRDSQDGEVQGDPRHSVAFGANMDNQLGISMMPDSLITNSLVGSRKDVSNNISSGGGMLSSYE
NPKDAQPELSSSMVSQSFGVPDMAFNSIDSTINEGSFMNRGAWAPPPQMPRMRTFTKVHKRGAVGRSIDIARYSGYEEL
KQDLARRFGIEGQLEDRQRIGWKLVYVDHENDVLLVGDDPWEEFVNCVRCIKILSPQEVQQISLDGDFGNNVQNQACSS
SDGGNV

>Solyc05g047460

MKTPGNGAGGGNVNPAEGEKKNLNPELWQACAGPLVNLPVAGTHVVYFPQGHSEQVAASIKKDVEAQIPNYPNLPAKLI
CLLHNVTLHADPETDEVYAQMTLQPVPSFDKEALLRSDLSMKANKPQPEFFCKTLTASDTSTHGGFSVPRRAAEKIFPP
LDYSLQPPAQELVARDLHDNIWTFRHVYRGQPKRHLLTTGWSLVVSGKRLFAGDSVLFIRDEKHQFLLGIRKANRQPTN
LSSSVLSSDSMHIGILAAAAHAAANNSPFTVFYNPRAGPSEFVIPLAKYYKATYSSQISLGMRFRMMFETEESGTRRYM
GTITGISDLDPVRWKNSQWRNLQVGWDESTAGERINRVSIWEIEPITAPFLICSSPFFSSKRPRQPGMPDGDYSDMDGM
FKRTMPWLGDDFGMADPQGLPGLSLIQWMNMQKNPSLANPMIPNYMNSLSGSALQNLAGADLSRQLGMAAPQFQQQQM
QHNLQFNNAHRPNQQLDQLQKLPAAALNSLDSIMQSQQQLSDVSQQPRQNLTTQSLPTTQVHTQHIQAQSLGQSQNVLP
PQQSVQNQNQLQRNLPQSLSQQHPQQQILGQTQQQSFMSSQAPDPVNQQQHFSDNQAQLQMLQKLHQQQKSLLAQQSGL
QQPSQLGSIQDHQKQLMDASQNFSRSLATNQMLDASQTTSTSLPHSQVVQQQMTRINSPSNLRFSQPTQQPKLQQQQSG
NLSDLSGPVNYPLPRTSYQLSANGSNLTGTAGGGQSVVIDDVPSWSTSVSTNNCHSVVQPNMNGRITGARDEMTHCSGP
LEVMSANNNLQPKSDVKPSVNVVSKSQNHGFLAPQTLNTSGIQFDYLDSSSSATSACLSQNDVQLQQTATDPLSGSSQP
LIFRDSPDGGEVQGDPRNNVAFGAANMENNQLGLPMIPDPLITKSSMGSRKDFSDNLSSGGGMLSSYENPKEAQPELLA
SMASDYVTFNSIDSTINDGSFMDRGAWEPPPQLPRLRTYTKVYKRGAVGRSIDIARYSGYEELKLDLARRFGIEGQLED
RQRIGWKLVYVDHENDVLLVGDDPWEEFVSCVRCIKILSPQEVQQMSLDGDFGGSVLQNQDCSSSDAGDV

>Solyc05g056040

MVDQLRFSQQKYLNHMPFKGDDDLCREIWKACSGSLLDVSKAGERVYYFPRLHVEQLEQSSNQELIEKLQLSNLPPKIL
CRVLHIRLLVEHETEEVYAETILIPNQDQNEPTAADFSPLDNPRPQFQSFCKCLTQSDIKSNWGLSVPLKDAVKCFPPL
DMRQEKPCQELIAKDLKGNEWRFKHAHQGQPRRHSLTNGWSTFVTSKKLLAGDLVVFLRDETGKLHVGIRRLSYQHCSV
GASTFSRQSMEVVLAVASHAFATKSLFFVYQKPCYNKSSQFIMSMSKYFEGGNHGIGVGMISRMQIESEDYCHVRRTND
LEQISLSQSQQTTNFMLEEDQYMQDSEAVLDCAQSTMIDLEIRQQTIGSLNNIHCFAPVEDNGLQLHAAVARENNEGSI
PKDTVSTHTRDEDLYELFKDFDFPDSTNTSLDTIALDLGSDWFRSTLQEQEETVTPSQPNASEDHSRRTSSVLEHPNSF
RVMSISSNPSQIPYEGPGRGDEQVPWGGYQVARRTLPILSRVVSRYPDSLVNFRVASSILQSVYLEILAELVYFLDNLT
IVNLSKDQFNVARQHIWDLKLSGIEIGWLENRLLHIDEVFSMESLLQRRQALTRRMEETFKQLDGELGCIDKELHELSL
KVGPNSPMRLQSVLEGLL

>Solyc11g069500

MKEVLEKCVDSQLWHACAGGMVQIPPVNSKVYYFPQGHAEHTLMNVDFSALPRSPALILCRVAAVKFLADPETDEVYAK
IRVVPVGNKGNDFDDDDDILGSNESGTAEKPNSFAKTLTQSDANNGGGFSVPRYCAETIFPRLDYTADPPVQTVTAKDV
HGESWKFRHIYRGTPRRHLLTTGWSSFVNQKKLVAGDSIVFLRAENGELCVGIRRAKRGGIGGPEAPSGWNSGAGNYGG
FSAFLREEMSKNGNLTSPTRSLRGKGRVRPESVVEAAYLASSGQPFEVVYYPRANTPEFCVRASSVNAAMRIQWCSGMR
FKMAFETEDSSRISWFMGTISSIQLADPIRWPNSPWRLLQVAWDEPDLLQNVKHVSPWLVELVSNMPVIHLSPFSPPRK
KLRLPPDFSLDSQFQLPSFSGNPLRSSSPFCCLSDNITAGIQGARHAQFGVPLLDLHLSNKLPSGLLPPSFQRVAANSQ
LPNVINKCQNDRNDNISCLLTMGTSSKTLDKNDSVNTPRFLLFGQPILTEQQISNGCSVSAPQVVQTGKDLGRIQPINE
KHPSEQKGSIQDNLSSATFFWNRGYHAAELGVLNTGHCKVFLESEDVGRTLDLSVMGSYEELYKRLANMFGLERPDMLT
RVLYHDATGAVKHTGDEPFSDFVKSAKRLTILMNSSSNIKRKWLTGLATAERGLDSSNQAGPLSIFA

>Solyc09g007810

MITFMDPKEKVKEVEKCLDSQLWHACAGSMVQMPSISSKVFYFPQGHSEHASGNVDFRSSIRIPSYIPCKVSAIKYMAD
PETDEVFAKIRLIPVGRNEVEFDDDGVVGMNGSDNQDKPSSFAKTLTQSDANNGGGFSVPRYCAETIFPRLDYSADPPV
QTILAKDVHGETWKFRHIYRGTPRRHLLTTGWSTFVNHKKLVAGDSIVFLRAENGDLCVGIRRAKRGIGGGPETSSGWN
PAGGNCMVPYGGFSSFLREDENKLMRNGNGNNGGNLMNKGKVKAESVIEAANLAASGQPFEVIYYPRASTPEFCVKSSL
VKSALQIRWCSGMRFKMPFETEDSSRISWFMGTISSVQVSDPIRWPDSPWRLLQVTWDEPDLLQNVKRVSPWLVELVSN
MPTIHLSPFSPPRKKLRLPQHPDFPLDGHLPMPAFSGNHLLGPNSPFGCLPDNTPAGMQGARHAQYGLSLSDLHFNKLH
SSLFPVGFPPLDQAAAAPRRPLNSPMISKPCNNENISCLLTMGNSAHSTKKSDIGKAPQLVLFGQPILTEQQISLSCSG
DTVSTVRTGNSSSDGNADKIGNVSDGSGSALNQRGLTERSPCDTFQSDPNTEIGHCKVFMESEDVGRTLDLSLLGSYEE
LCRKLANMFGIDNSEMLNHVLYRDTTGSVKQLGDEPFSDFMKTARRLTILTDSSSDNVGIRE

>Solyc10g086130

MFLQNLLMADNKSNCFDSRLWQACAGTMVKMPAVDSIVLYFPQGHAEHAGVNVEFRSDVKIPSYIPCRVSSIKYMAERE
TDEVFAKIRLTPVRLSEFFETPEEEGMVKIGSDNSRKPLSFAKTLTQSDANNGGGFSVPKNCADTIFPTLDYNVNPPVQ
TLSATDIHGKSWQFRHIYRGTPERHLLTTGWSTFVNQKKLVAGDSIVFLRNENDKISIGIRRIKKKSVAMEPETSPWWF
PSVGNLTIPRGGFSAFLRDDHNTNSSWSLINRGNVKAESVIEATKLATNGQPFEVIFYPQSTTPEFFVKASRVKAALQI
PWCSGMRFKMPFETEDLVISWFMGTISSVQANDPSQWPDSPWRMLQVTWDEPNLLHNVMCVNPWLVEPVSNMPTINFNP
YTPPLKKLRLSHTSDFPLNGHLPMSGFPNNHLEFSIDPPMSSFPIHHLLRPYGCPPNNTPVGMQGARHAPYNLSLPDIH
TNNLLSSLSPVGFPSLHHVVASPNTSNNTMIPKPSRNAGISSLLTLGSSTQTNKKFDSEKTTQFVLFGQPIVIEQQTSQ
SNSRISVSPRHATNSFSDGNEYKKENTSDSSDTSFVHNSVPDYLPSKSFRSEENVEIGHSKVFIESEDIGQTKQLGEW

>Solyc06g075150

MEVVEEKCVDSLFWHVCTGSMVQIPPVNSKVFYFPQGYAEHTFTNVDFTVLARIPAMILCRVDAVKFLADTETDEVYAK
IRLIPVEDFEDDSVVEETEKPAFFAKTLTQSDANNGGGFSVPRYCAETIFPKLDFTADPPVQVVKAKDVHGVTWNFRHI
YRGTPRRHLLTSGWSAFVNKKKLVAGDSVVFVKAENDELCVGIRRVKRGGIGGPETQSGWKSTACSYGGFVTEDENSST
NGNLISYGERFRDKGKVSPDEVVRASCLAANGQPFEIVYYPGASTPEYCVKASSVRAAMSVQWCSGMRFKMAFETEDFS
QISWFMGSISSVQVVDPIRWPHSLWRLLQVTWDEPDLLQNVKSVNPWLVELVSNMPDINLSHNSPPRKRLCLPQEFPFD
GQFPLPSFSGNPLTSSSYSRYPSDSITAGIQGARHVRFGVPLLDLHRSEKLQLGVLQPPVSQQADADSEIPIGTSKVQK
ESNENISCLLTMGTSSQMEKADNVKTPRFLLFGQPILTEQQMSSVLSTHAPPQVQTERNSDWAQLKTERITPDWKCLSE
SLSSTFLWNKGYHAAELGASTDHCKVFLDSEDVGRTLDLSVLGSYAELYKRLADMFEMERLDMVTRVLYLDATGASKQI
GDEPFSDFIKTAKRLTILKKSGNSATRKWLTDLPILNVV

>Solyc12g006350

MKVSTSGFNSQPEEGEKKSLNSELWHACAGPLVSLPHVGTRVVYFPQGHSEQVAASTNKEINGHIPSYPGLPPQLICQL
HNVTMDADVETDEVYAQMTLQPLTPQEQKDVCLLPAELGTLSKQPSNYFCKTLTASDTSTHGGFSVPRRAAEKVFPPLD
YSQQPPVQELIGKDLHGNEWKFRHIFRG

>Solyc12g006340

MKVSTSGFNSQPEEGEKKSLNSELWHACAGPLVSLPHVGTRVVYFPQGHSEQVAASTNKELNGHIPSYPGLPPQLICQL
HNVTMDADVETDEVYAQMTLQPLTPQEQKDVCLLPAELGTPSKQPSNYFCKTLTASDTSTHGGFSVPRRAAEKVFPPLD
YSQQPPVQELIGKDLHGNEWKFRHIFRGEFLFRELIE

>Solyc00g196060

MYNSLCIYKGINCIICQPKRHLLTTGWSVFVSAKRLVAGDSVIFIWNENNQLLLGIRRANRPQTVLPSSVLSSDSMHIG
LLAAAAHAAATNSRFTIFFNPRACPSEFVIPLAKYAKAVYHTRVSVGMRFRMLFETEESSIRRYMGTITGIGDLDPVRW
PNSHWQSVKVGWDESTAGERQPRVSLWEIEPLTTFPMYPSPFSLRLKRPWPPGLPSFPGLSNGDMTMNSQLPWLHGGMG
DQGIQSLNFQGFGVTPFMQPRFDASMLGLQPDILQAMAALDSSKLANQPLMQFQHIPSTSASSIQSQLLHPSNLQHTFL
QGLPENQLISQAQMLQQQLQCHQSYNTQQQQLQRQXXX

>Solyc11g013480

MQAPTVADREVDPIVWRAIAGNSVKIPPVGTRVYYFPQGHAEHATFTSPAVMSPGMPAFILCRVLSVRFLAESDTDEVY
ARIFLHPISQSEVDEVTMREEEVVEDEIVSFVKILTPSDANNGGGFSVPRFCADSIYPRLDFEAEPPVQNLSIRDIKGV

AWEFRHIYRGTPRRHLLTTGWSKFVNSKQLVAGDSAVFMRRTANNQLYVGVRRAIRRNDDSQKWTSSFLMREHINNGGS
PDVSWGIRKGRMTMEAVAAVAEKAARGVPFEVSCYPRDAWAGFVVKAQEVQMALNMPWTVGMRVKMAVEAEDSSRTACY
QGTVSSVILNESGPWRGSPWRMLQII

5. *S. tubersomem* ARFs

>StARF6a

MRVSSSGFNPQQEEAGEKKCLNSELWHACAGPLVSLPPVGSRVVYFPQGHSEQVAASTNKEVDAHIPNYPGLPPQLICQ
LHNLTMHADVETDEVYAQMTLQPLSPQEQKDVCLLPAELGIPSKQPTNYFCKTLTASDTSTHGGFSVPRRAAEKVFPPL
DYSQQPPCQELIAKDLHGNEWKFRHIFRGQPKRHLLTTGWSVFVSAKRLVAGDAVIFIWNENNQLLLGIRRANRPQTVM
PSSVLSSDSMHIGLLAAAAHAAATNSRFTIFYNPRASPSEFVIPLAKYAKAVYHTRISVGMRFRMLFETEESSVRRYMG
TITGISDLDPVRWPNSHWRSVKVGWDESTAGERQPRVSLWEIEPLTTFPMYPSPFSLRLKRPWPSGLPSLPGFPNGDMT
MNSPLSWLRGDMGDQGMQSLNFQGFGVTPFMQPRMDASMLGLQPDILQTMAALDPSKLANQSLMQFQHSIPNSSAPLSQ
SQMLQPSHSQQNLIQGFSENHLISQAQMLQQQLQRRQNFNDQQQLLQPQLQRHQEVNSQFQHQQQTKTISGLSQMASAT
HPHLSHLQVLSSTGSPQTFSDILGNHVNASSNSNMQSLLSSFSCDGASTVLNVHETHPLVSSSSSSKRIALESQLPSRV
TPFVVSQPEDVIAHNTKVSDLSSLLPPFPSRESFSDYRGVEDSQSNALYGFTDSLNILQTGMSNMKGSSGDNGSLSIPY
ATSTFTSTVGNEYPLNSDMTASSCVDESGFLQSSENGDQANPTNRIFVKVQKSGSFGRSLDISKFSSYHELRSELARMF
GLEGLLEDPERSGWQLVIVDRENDVLLLGDDPWQEFVNNVWYIKILSPHEVQQMGKEGLDLPNGVQAQTQTLPGNVNGC
DDYMNQKGSRNTMNGIPLGSLDY

>StARF6b

MKVSTSGFNSQPEEAGEKKCLNSELWHACAGPLVSLPHVGTRVVYFPQGHSEQVAASTNKEINGHIPNYPGLPPQLICQ
LHNVTMDADVETDEVYAQMTLQPLTPQEQKDVCLLPAELGTLSKQPNNYFCKTLTASDTSTHGGFSVPRRAAEKVFPPL
DYSQQPPVQELIGKDLHGNEWKFRHIFRGQPKRHLLTTGWSVFVSAKRLVAGDSVIFIWNENNQLLLGIRRANRPQTVL
PSSVLSSDSMHIGLLAAAAHATATNSRFTIFFNPRACPSEFVIPLAKYAKAVYHTRVSVGMRFRMLFETEESSIRRYMG
TITGIGDLDPARWPNSHWQSVKVGWDESTAGERQPRVSLWEIEPLTTFPMYPSPFSLRLKRPWPPGLPSFPGLSNGDMT
MSSQLPWLHGGMGDQGIQSLNFQGFGVTPFVHPRFDASMLGLQPDILQAMATLDSSKLANQPLMQFQHIPSGSASSIQN
QLLHPSNLQHTFLQGLPENQLISQAQMLQQQLQCHQSYNAQQQQLQRQQLYHDQQLQEPHQVHLQRQDQQQTKAQLCSA
TQSQLSHLQVLGSTGSQQTFSDLVGNHINTSNNRSTMQSLLSSFSRNGASTFLNMPETNSLVSPSSSSKRIALESQIPS
QAPYIVTQAEVLTVPNTKVSDFSTLFSPNPGRQVLDYQAVAVSQNNALFGVNGMSNLKGNSPENRSLPMPYATSAFTST
VGSEYPVTSDMTTSSCVDESGVLQSSENVDQANSLTETFVKVYKSESFGRSLDISKFSSYNELRSELARMFGLEGLLED
PERSGWQLVFVDRENDVLLLGDDPWHEFVNSVWYIKILSPLEVQQMGKEGLDLPSAGKTQRITSNGNGNGCDDFMNRNH
SCNIMNGIPLGSLDY

>StARF8

MTLQPLTPQEQKDTYLPVELGIPSRQPTNYFCKTLTASDTSTHGGFSVPRRAAEKVFPPLDFSQTPPCQELIARDLHDI
EWKFRHIFRGQPKRHLLTTGWSVFVSAKRLVAGDSVLFIWNEKNQLFLGIRRATRPQTVMPSSVLSSDSMHIGLLAAAA
HAAATNSCFTVFFNPRASPSEFVIPLSKYIKAVYHTRVSVGMRFRMLFETEESSVRRYMGTITGIGDLDPVRWANSHWR
SVKVGWDESTAGERQPRVSLWEIEPLTTFPMYPSLFPLRLKRPWYPGTSSFQENNSEAINGMAWLRGESSEQGPHLLNL
QSFGGMLPWMQQRVDPTMLRNDLNQQYQAMLASGLQNFGSGDLMKQQLMQFPQPVQYVQHAGSLNPLLQQQQQQAMQQ
TIHQHMLPAQTQDNLQRQQQQHVSNQTEEQSHHHSYQEAYQIPNSQLQQKQPSNVPSPSFSKPDIADPSSKFSASIAPS
GMPTALGSLCSEGTSNFLNFNILGQQPVIMEQQQQQQQQQKSWMAKFAHSQLNTGSNSPSLSGYGKDTSNSQETCSLD
AQNQSLFGANVDSSGLLLPTTVSNVATTSIDADISSMPLGTSGFSNSLYGYVQDSSDMLHNVGQVDAQTAPRTFVKVYK
SASLGRSLDITRFNSYHELRQELGQMFGIEGFLEDPQRSGWQLVFVDRENDVLLLGDDPWEEFVNNVWYIKILSPEDVQ
KLGKEEVGSLNRGLPERMSSNNSADGRDFMSGLSSIGSLEY

>StARF19a

MKAPSNGYLPNSGEGERKLMNSELWHACAGPLVSLPPVGSLVVYFPQGHSEQVAASMQKETDGIPSYPNLPSKLICMLH
NVTLHADTETDEVYAQMTLQPVNKYDQEALLLSDMGLKQNRQPAEFFCKTLTASDTSTHGGFSVPRRAAEKIFPPLDYA
MQPPAQELMARDLHDQAWTFRHIYRGQPKRHLLTTGWSVFISSKRLCAGDSVLFIRDDKSQLLLGIKRTNRQQPALSSS

VISSDSMHIGILAAAAHAAANNSPFTIFYNPRASPSEFVIPLAKYNKAMYAQVSLGMRFRMMFETEESGVRRYMGTITG
VSDLDPIRWKSSEWRNLQVGWDESTAGERPSRVSIWDIEPVVTPFYICPPPFFRPKFPKQPNFPGDESDIENVLKRGMP
WINDELGLKDSQNSIFPGLSLVQWMSMQQNNHVPVAQSGLPSVLHSNIGSDDHSKLLNFQSPTLATPGLQFNKPNQLNQ
PFGQIQQPPLAWAQQQQQSLQSPVSAQQQQPTLQQQQQQHTLQQQQPTLQQQQHTLQQQQHTLQQQQQQQQQQQQHTLQQ
QQQQQHTLQQQQQQHTLQQQQQQQHTLQHQQQHTLQQSQQHMLQQQPQLHQQAQQQLQQQQRPSQQQQLTGNSSPVNRC
VSPNQIPNQTFPQAAVYGQLQQQQVLSASTQSQQNVPVNRNPFPSTSLAQDFQFQQQVEQQSNLLQKSQQQQTIPQQVP
LQLLQQSLMQRPQVQPSSQQSLTEQQLQLQLLNKLQQQQQQQQQQAQLLSPVSSTLEPRMPQQQQNRQPQELQIAHQQL
SSNIVTTATHLQSTHHAFNQLQSQHKSPITIKALSGGTDGDAPSCSTSPSTNNFQVSPPNFLTKNQGQAILVDESVVDP
SQEQNKSEFRIKHELVFSKGSEQSKYKGNNTENLEAASSTTSYGLDSSGFNFSLPALCVDGDVQSHSRNSLPSAANNID
GLNPDALLSRDYDSGKDMQNLFSPFGNAPRDIETELSDAGINSQQFGVPNMAYKPRCTNDLAVNDNGVLNNNAWTNQTQ
RMRTYTKVQKRGSVGRTIDVTRYIGYDELRHDLARMFGIEGQLEDPQRTEWKLVYVDHENDMLLVGDDPWEEFVSCVQS
IKILSSAEVQQMSLDGDLGNVPVPNQASSGTDSGNAWKGHYDDNSAASFNR

>StARF19b

MKTPANTAGVQQQQQHTVNGNHPAEVEKKSINPELWQACAGPLVNLPAAGTHVVYFPQGHSEQVAASMKKDVDAQIPNY
PNLPSKLVCLLHNITLHADPETDEVYAQMTLQPVPSFDKEALLRSDLSMKLNKPQTEFFCKTLTASDTSTHGGFSVPRR
SAEKIFPPLDYSMQPPAQELVARDLHDNLWTFRHIYRGQPKRHLLTTGWSLFVSGKRLFAGDSVLFIRDEKQQLLLGIR
RANRQPTNLSSSVLSSDSMHIGILAAAAHAAANNSPFTVFYNPRASPSEFVIPLAKYYKATYSCQVSLGMRFRMMFETE
ESGTRRYMGTITGISDLDPVRWKNSQWRNLQVGWDESTAGERRNRVSIWEIEPVTAPFFICPTPPFFRSKRPRLPGMPD
DDCSDLDGLFKRTMPWLGDDFGMKDPQGLPGLSLVQWMNMQQNPSLANSMQPNYLHSLSGSVLQNVGGGADLSRQLGLP
APQLPQQNTLQFGAQRPTQQVQQLDQLQKLPTTTLSPAGSIMQSQQQLSDISQQPRQNLINQSVPTNHVQAQLLQAQSL
VQSQNVLQQQQSFQNQLQRNLPQNLPQQQQIMNQTQQQSFMPPQPNDPLNQQLHFSDNQLQMQLLQKLQQQSLLAQQSL
LQQPSQLMPIQDQQKHIDVSQNFSRSLATSQMLDMSQTTSTSTTLSQPQVAQQQMTINNSQSNLRFAQPNQHMKQQQQQ
QPGILPEIPGQVGQILPPTTNQLSANCSSFLTGAVGGGQSVVTDDIPSCSTSPSTNNCQNVVQPIMNGRIHRGTAAADE
TTQSSLPLLSSSGLEAMSPNRNLVKDLQQKPDVKPSLNISKSQNHGFSTPQTYLNTAVPQMDYLDSSSSATSVYFSQND
VQLQQTTNPMSFSSQAVVFRDSQDGEVQGDPRNSVAFGANMDNQLGISMMPDSLITNSLVGSRKDVSNNISSGGGMLSS
YENPKDAQPELSSSIVSQSFGVPDMAFNSIDSTINEGSFMNRGAWAPPPQMPRMRTFTKVHKRGAVGRSIDITRYSGYE
ELKQDLARRFGIEGQLEDRQRIGWKLVYVDHENDDLLVGDDPWEEFVNCVRCIKILSPQEVQQMSLDGDFGYNVQNQAF
SSSDGGNM

>StARF19c

MKTPGNGAGGGNVNPAEGEKKSINPELWQACAGPLVNLPVAGTHVVYFPQGHSEQVAASIKKDVEAQIPNYPNLPSKLI
CLLHNVTLHADPETDEVYAQMTLQPVPSFDKEALLRSDLSMKANKPQPEFFCKTLTASDTSTHGGFSVPRRAAEKIFPP
LDYSLQPPAQELVARDLHDNIWTFRHVYRGQPKRHLLTTGWSLVVSGKRLFAGDSVLFIRDDKHQFLLGIRKANRQPTN
LSSSVLSSDSMHIGILAAAAHAAANNSPFTVFYNPRAGPSEFVIPLAKYYKATYSSQISLGMRFRMMFETEESGTRRYM
GTITGISDLDPVRWKNSQWRNLQVGWDESTAGERINRVSIWEIEPITAPFLICSSPFFSSKRPRQPGMPDGDYSDMDGM
FKRTMPWLGDDFGMTDPQGLPGLSLIQWMNMQKNPSLANPMIPNYMNSLSGSALQNLAGADLSRQLGMAAPQFQQQQMQ
HNLQFNNAHRPNQQLDQLQKLPAATLNPLDSIMQSQQQLSDVSQQPRQNLTNQSLPTTQVHTQHMQAQSLVQSQNVLPP
QQSVQNQNQLQRNLPQSLPQQHPQQQILSQTQQQSFMPSQPPDPVNQQQHFSDNQAQLQMLQKLHQQQKSLLAQQSGLQ
QPSQLGPIQDHQKQLMDASQNFSRSLATNQMLDASQTMSTSLPHSQVVQQQQMTRINSPSNLRFSQSTQQPKLQQQQSG
NLSDLSGPVNYSLPRTSYQLSTNGSNLTGTAGGGQSLVIDDVPSWSTSVSTNNCHSVVQPNMNGRITGARDEMTHCSGP
FEVMSANNNLQPKSDVKPSVNVVSKSQNHGFLAPQTLNTSGIQFDYLDSSSSATSACLSQNDVQLQQTATDPLSCSSQP
LIFRDSPDGGEVQGDPRNNVAFGATNMNNNQLGLPMIPDPLITKSSMGSRKDFSDNLSSGGGMLSSYENPKEAQPELLA
SMASEYVTFNSIDSTINDGSFMDRGAWEPPPQLPRLRTYTKVYKRGAVGRSIDIARYSGYEELKLDLARRFGIEGQLED
RQRIGWKLVYVDHEKDVLLVGDDPWEEFVSCVHCIKILSPQEVQQMSLDGDFGGSVLQNQDCSSSDAGDV

>StARF5

MGSVEEKNKPGSLVSGAHTLLEEMKLLKGMQDHTGGGRKHISSELWHACAGPLVTLPQVGSLVYYFPQGHSEQVAVSTN
RTATSQIPNYPNLASQLLCQVHNVTLHADKETDEIYAQMSLQPVNSEKDVFPIPDFGLKPNKHPTEFFCKTLTASDTST
HGGFSVPRRAAEKLFPPLDYSMQPPTQELVVRDLHDNTWTFRHIYRGQPKRHLLTTGWSMFVGAKRLRAGDSVLFIRDE
KSQLLLGVRRANRQQTSLPSSVLSADSMHIGVLAAAAHAAANRSTFTIFYNPRACPSEFVIPLAKYRKSVYNTQLSVGM
RFGMMFETEESGKRRYMGTISGISDLDPLRWPGSKWRCLQVEWDEPGCGDKQNRVSPWEVETPESLFIFPSLTAGLKRP
YQSTFLGAPTEWDSLMQHRPFMRVPENVYGDLQSSSISNLWSEQLMKMLIRPPPGLTGLQCGVPTVQDIKVALPQEARN

VIQPAGNQKPELITVEATPAQSETNSEVILNQPVGVVNSISSQQATLQAKSQPLEKVETDVIGKSYEPRKETCNSSVKL
DQFQCNEDKVTIKPASPHDLPTASATASHHNSFSQLQATPWLIPHNPQIDSAGSNNILQCPTNNEWNLSSLQSAAGLLR
YPVSTSTLTKHDNSFMLPDTIGHGLAPIGQDLWDHQLNDVKCFSQTNLQVPLDITNMQFLPDSYDFKDLSEESHNQSDI
YSCLNFDSNSGSTVIDNSVSSTVLDEFCNLKHTDFQNPSDFLLGNISSSQDVQSQITSASLADSQNFSVQEFADNSGGA
SSSNVNFDECNLLQNSSWQQVAPRVRTFTKIQKTGSVGRSIDVSGFKNYEELRSEIERMFGLEGLLNDTRGSSWKLVYV
DFEHDVLLVGDDPWEEFVGCVRCIRILSPTEVQQMGEEGMQLLNSAGLQGINGSTSEFPN

>StARF3

MMCGLIDLNTVDNDDAGEETAAPVSLDSPASSSVASGSSDLTSSTTPAVASVCMELWHACAGPLISLPKKGSAVVYLPQ
GHLEHLSEYPPIACNLPPHVFCRVVDVKLQADAATDEVYAQVSLVPDNQQIEQKWKDGDIDADTEEEEIEGAGKSITPH
MFCKTLTASDTSTHGGFSVPRRAAEDCFAPLDYRQQRPSQELVAKDLHGIEWKFRHIYRGQPRRHLLTTGWSAFVNKKK
LVSGDAVLFLRTGDGELRLGVRRAAQAKTCSSYLARYSKPLNVSGIEDAVNVISSRNVFNICYNPRGNSSDFIVPYHKF
SKTLAHPFSAGMRFKMRVETEDAAEQRFTGLVVGFSDVDPVRWPGSKWRCLLVRWDDLDVSRHNRVSPWEIEPSGSAPV
SSSLVMPSAKRTRVGFPISKADFPIPREGIAVSDFGESSRFQKVLQGQEILRMHAPYGGLDARSPRPAGIRCFPGFPSS
GISRMGNSIRPLLGDTDKSHESIGFSESLRFNKVLQGQEIFTSPPYGRAQAGIQMQEKGRTGIFVGIQVPNHGNRWPAP
NQDNNTRCKPINPVSASSPPSALTFQHPSPPASKFQAMFNHKHGKHDLGNQASLDMSENCCRYLTSGSHTEDISRKEGT
QGMSSFGFLKEQKQTGISYLSLGTQSSFKGNQNLVSTCKTSCRIFGFPLTESKISATRAETPSEAVYSLGLETTFLPSG
DGKLQPGPPLVTNVVGTNFTKVNDLYAARDVLLDIAL

>StARF4

MEFDLNHALVSEVEKNVCCNEECDKGGGGGCVNCSLYTSTTSSCSSNVSSSSSLALTSIYKELWHACAGPLTSLPKKGN
VVVYFPQGHMEEAVSAFPFSPIKIDFPTFGLQPQIFCRVEDVQLLANKENDEVYTQLTLLPLPESMAISLEGKEHEDSG
TDEEGNGVNPGKSASHMFCKTLTASDTSTHGGFSVPRRAAEDCFPPLDYKEQRPSQELIAKDLHGVEWKFRHIYRGQPR
RHLLTTGWSIFVSQKNLVSGDAVLFLRGEGGDLRLGIRRAARPRNGLPESIIKSQYSGPDVLSSVASALSAKSTFHVFY
SPRASHADFVVPYQKYVKAINTRIPVGTRFKMKFDLDDSPERRYSGVVTGISDMDPFRWPNSKWRCLMVRWDEDIMSNH
QERVSPWEIDSSVSLPPLSIQSSPRLKKLRTSQQAPSVLDGHFAGGSALLDFEESIRSSKVLQGQENLGLISPPYGCDK
PVRPLDFELQRVARHNLMPNGVENIIVGDFVKTQPPTTYTGFLESNRFPKVLQGQEICSLRSLTGKGDVNFGAWGKPEF
GCNVFGTYQRPRANFYPLASEGARNVFLPYNAMYRAGQDPVVPSYITTFQRENPTLNQNSIQNVVRREEVGMPKFVNEQ
RPPEMSKVSIPENHFKNENDGSFNAQASCKLFGFSLTKEPSTPSSQSSGKRSCTKVHKQGSLVGRAIDLSRLNGYDDLL
VELERLFNMEDLLRDPNKGWRILYTDSENDMMVVGDDPWHEFCEVVSKIHIYTQEEVEKMTIEGISDDTQSCLEEAPAI
MDVSKSSSVGQPDSSPTVIRI

>StARF13

MVDQPWFSQQKYPNHMSFEGDDGLRREIWKVCSGSSLDVPKAGERVYYFPRLHVEQLEQSSNQELIERLQLSNLPPKML
CRVLHIRLLVEHETEEVYAEIRLLPNQDQNEPTAPDFSPLDTPRPQFQSFCKCLTQSDIKSNWGLFVPLKDAVKCFPPL
DMRQEKPSQELIATDLQGNEWRFKHAYQGQPRRHSLTNGWSTFVTSKKLLAGDLVVFLRDETGKLHVGIRRLSYQRCSI
GASTFSRQSVEGALAVASHAFATRSLFSVYHKPCYNKSSQFIMSLSKYFEGGNHGRGVGMISRMQHEGEDSSHVRRTND
LDQISLSQGQQTTNLMLEEDQYMQDSEAVLDCAQSTMTDLEIRQQTTGSHNNVHCFSPVEDNGLQLHTAVARENNEGSI
PNDTVSIQTRDEDLYELFEDFDFPDSINTSLDTIALDLGSDWFRSTLQEQEETITPSHPNASEDHSRRTSSVLEHPTSF
QVMSISTNPSQIPYEGPGRGDEQVPWGGYQVARRTLPILSRVVSRYPDSLVNFRVASSILQSVYLEILAELVYFLNNLT
IVNLSKDQFNVARQHIWDLKLSGIEIGWLENRLLHIDEVFIMESLRQRRQALTRRMEETFKQLDGELGCIDKELHELSL
KVGPNPPMRLHSVLEGLL

>StARF17

MQAPTASDREVDPIVWRAIAGKSVQIPPVGSRVYYFPQGHAEHATFTSPGVLSPGIPAFILCRVLSVRFLAESDTDEAF
AKIFLHPISPSEVDEVTVSEEEEVEDKIVSFVKILTPSDANNGGGFSVPRFCADSIFPRLNFEAEPPVQNLSIRDIKGG
VWEFRHIYRGTPRRHLLTTGWSKFVNSKQLVAGDSAVFMRRTANDQLYVGVRRAIKRNDNSQKWTSSFLMREHINSGGS
PDVSWGIRKGRMTMEAVAAVAEMAARGVPFEVSCNPRDDWADFVVKAQAVEMALNIPWTVGTRVKMAVETEDSSRTACF
QGTVSSVILTESGPWRGSPWRMLQDRQVRVATELVVGQRTALKQL

>StARF16

MDPKDKVKEIEKCLDSQLWHACAGSMVQMPSISSKVFYFPQGHSEHASGNVDFRSSIRIPSYIPCKVSAIKYMADPETD
EVFAKIRLIPVGRNEVEFDDDGVVGMNGSDNQDKPSSFAKTLTQSDANNGGGFSVPRYCAETIFPRLDYSADPPVQTIL
AKDVHGETWKFRHIYRGTPRRHLLTTGWSTFVNHKKLVAGDSIVFLRAENGDLCVGIRRAKRGIGGGPETSSGWNPAGG
NCMVPYGGFSSFLREDENKLMRNGNGNGNNGGNLMNKGKVKAESVVEAANLAASGQPFEVIYYPRASTPEFCVKSSLVK
SALQIRWCSGMRFKMPFETEDSSRISWFMGTISSVQVSDPVRWPDSPWRLLQVTWDEPDLLQNVKRVSPWLVELVSNMP
TIHLSPFSPPRKKLRLPQHPDFPLDGHLPMPAFSGNHLLGPNSPFGCLPDNTPAGMQGARHAQYGLSLSDLHFNKLHSS
LFPVGFPPLDQAAAAPRRPLNIPMISKPCNNENISCLLTMGNSAHSTKKSDIGKAPQLVLFGQPILTEQQISLSCSGDT
VSTVRTGNSSSDGNADKIGNVSDGSGSALNQRGLTERSPCDTFQSEPNTEIGHCKVFMESEDVGRTLDLSLLGSYEELC
RKLANMFGIDNSEMLNHVLYRDTTGSVKQLGDEPYSDFMKTARRLTILTDSSSDNVGIRE

>StARF10a

MKEVLEKCVDSQLWHACAGGMVQIPPVNSKVYYFPQGHAEHTLMNVDFSALPRSPALILCRVAAVKFLADPETDEVYAK
IRVVPVGNKGNDFDDDDDILGSNESGTTEKPNSFAKTLTQSDANNGGGFSVPRYCAETIFPRLDYTADPPVQTVTAKDV
HGETWKFRHIYRGTPRRHLLTTGWSSFVNQKKLVAGDSIVFLRGENGELYVGIRRAKRGGIGGPEAPSGWNSGAGNYGG
FSAFLREEMSKNGNLSSPTGSLRGKVRVRPESVVEAAYLAASGQPFEVVYYPRANTPEFCVRASSVNTAMRTQWCSGMR
FKMAFETEDSSRISWFMGTISSIQLADPIRWPNSPWRLLQVAWDEPDLLQNVKHVSPWLVELVSNMPVIHHSPFSPPRK
KLRLPPDFSLDSQFQLPFFSGNPLRSSSPFCCLSDNITAGIQGARHAQFGVPLLDLHLSNNLPSGLLPPSFQRVAANSQ
LPNVINKYQNDRNDNISCLLTMGTSSKTLEKNDSVNTPRFLLFGQPILTEQQISNGCSVTAPQVVQTGKDLGRIQLINE
KHPPEQKGSIQDNLTSATFFWNRGYHAAELGVLDTGHCKVFLESEDVGRTLDLSVMGSYEELYRRLAKMFGLERPDMLT
RVLYHDATGAVKHTGDEPFSDFVKSAKRLTILMNSSSNIKRKWITGLATAERGLDSSNQAGPLSIFA

>StARF10b

MEVVEEKCVDSQFWHACTGSMVQIPPVNSKVFYFPQGHAEHTYTNVDFTLLPRIPAMILCRVDTVKFLADTETDEVYAK
IRLIPVEDFEDDSVVEKTEKPTFFAKTLTQSDANNGGGFSAPRYCAETIFPKLDLKADPPVQVVKAKDVHGETWNFRHI
YRGTPRRHLLTSGWSAFVNKKKLVAGDSVVFLKAENDELCVGIRRVKRGGIGGPETQSGWNSTTGSYGGFLTEDENSMI
RSCSNENLISYGGRFRDKGKVSPDEIVRDSYLAASGQPFEIVYYPGASTPEYCVRASSVHAAMSVQWCSGMRFKMAFET
EGSSRISWFMGSISSVQVADPIRWPHSPWRLLQVTWDEPDLLQNVKSVNPWLVELVSNMPDINLSHFSPPRKRLCLPQE
LALDRQFPLPSFSGNPLRSSSPFCYPSDNSTAGIQGARHVQFGVPLLDLHRSEKLQLGVLQPPASQQVDADSESPIGTS
KVQKESNENVSCLLTMGTSSQMEEADNVKTPRFLLFGQPILTEQQMSSVLSTHAPPQVQAERDSDWAQLKTERISQGWK
CLSESLSSTTFLWNKGYHTAELGASTDHCKVFLDSEDVGLTLDLSVLGSYAELYKRLADMFEMERLDMVTRVLYLDATG
ASKQIGDEPFSDFIKTAKRLTILKKSGNSATRKWLTDLPILNVV

>StARF18a

MGSEDLYRDLWKACAGPLVDVPRDAERVYYFPQGHIEQLEASTNQAVNQQIPQFNLSSKILCRVVHVQLLAETETDEVY
AQITLHPEAEQEEPSKPDPCPPDLPKRTVHSFCKILTASDTSTHGGFSVLRKHANECLPQLDMTQATPTQDLVAKDLHG
YEWRFKHIFRGQPRRHLLTTGWSTFVTSKRLVAGDAFVFLRDDNGELRVGVRRLARQQSPIPQSVISSQSMHLGVLATA
SHAITTQTRFVVYYKPRTSQFIVGLNKYLEAVSHGFSVGMRFRMRFEGEDSPERRFTGTIVGTGDISSQWSESKWRSLK
IQWDEPASMVRPDRVSPWEIEPFVASTCVDVAQPGIKSKRPRPLDLPRTEIAVASAASPFWCPGSGPTLEVSHLGGITD
VQSHDNQLFWSSKQNSCLSNGVSNTSCRTHLSGAWQHSMLANGSLNLLRDSIEDNKQLITRSALLDYGSPMSSKASSGL
LHDQVNRGNKREISSGCRLFGIDLRNNSNNTSTKAKEILGPNITSNCADEAPIVHDESEIDKDQNVEHLNPSDEKKQVQ
LEALPKDTLKQGPTSSRTRTKVQMQGVRVGRAVDLTALSGYDDLISELEKIFDIKGELCPRNKWEVVYTDDEGDMMLVG
DDPWLEFCNMVRRIFIYSSEEVKKMTPRCKLPILSLEGEGTMPSVDSELKAEG

>StARF18b

MASKNGCYQSQQKKNSSGKDDLYHELWQLCAGPIVDVPKEGESVYYFPQGHMEQLVASINQEMDQRVPSFNLKSKILCR
VINSHFLAEEDNDEVYVQITLMPEAPDKAEPTNPDPFLPEPVKPKVHSFCKVLTASDTSTHGGFSVLRKHANECLPPLD
MNQQIPTQELIAKDLHDMEWHFKHIFRGQPRRHLLTTGWSNFVSSKRLVAGDSFVFLRGDNGELRVGVRRLVRQQSSMP
SSVISSQSMHLGVLATASHAVTTQTLFVVYYKPRTTQFIVSLNKYLEAVNHGYSVGMRFKMQFEAEENPDRRFMGTIVG
VDDLSSQWKDSAWRSLKVRWDEPAAIARPDRVSPWEIEPYVSSIPNALVPPTAGKNKRHRLHSEIKISEPASSIASAVW
NPSLDSPQFNTSGINSSTNCTLTSRTESGWPLPHLNTAGMLVDETEDSRSASTWCGFPCVLAPQFGQGTNQPIVIPTDE
RKCNTKTTCRLFGIDLKKTSISTTEALLPPQPADISRVSAERAPPNMAPAGDSDQKSDLSVDFKDQMQGHLRLPLKEVQ

SKQSCSTRSRTKVQMQGVAVGRAVDLTILKGYNELTKELEEMFEIQGELQSRQKWGILFTDDEGDTMLMDDYPWQDFCN
VARKIFICSSQDMKKLTLSRADS

>StARF18c

MNFEGEEIPEKRFTGTIVGVEDSSSQWKDSKWRSLKVQWDEPASVSRPDRVSPWDIEPFVASVATPLVPPMGVKNKRHR
AHNEPKTSEPVPAAALAAWIPSTQFNPVIEGQSSDNPFSLHTSQTHSTTNSTFKVHEDGIWSASKVSASLNMLLDETEG
SKSASPRPAFPSFASSQFGKQNDLLLPCLDDERKRDTITSCRLFGIDLKSPSFGSVNENPPLEPANNSDGSAEGCSGNT
TSAGDSEDNSGLSRDSEDQKQEQLNLPPKEVHIKQISSTRTRTKVQMQGVAVGRAVDLTKLSGYDELLKELEEMFDIQE
ELHTRNKWEIVFTDDEGDMMLMGDYPWPEFCNIAKRIFICSSQDMKSFSARTKSPSCLESETTTST

>StARF1

MAHVAANHFGGGTHPGVSANGALYKELWHACAGPLVTVPREGERVYYFPQGHMEQLEASTHQGVDQHLPSFNLPAKILC
KVMNVQLRAESETDEVYAQITLLPEQDQGEITSPDPPPPEPEKCTVHSFCKTLTASDTSTHGGFSVLRRHADECLPQLD
MSQQPPWQELVASDLHGNEWHFRHIFRGQPRRHLLTTGWSVFVSAKKLVAGDAFIFLRGDSGELRVGVRRLMRQLNNMP
SSVISSHSMHLGVLATASHAISTGTLFSVFYKPRTSQSEFIVSVNKYLEARNHKLSVGMRFKMRFEGEEVPERRFSGTI
VGVGDNPSSRWPDSEWRSLKVHWDEPSSILRPDRVSPWDMEPLVAATPTNTQPPQRNKRARPSVLPSPVQELPALGMWK
SPVDSPSSFSYCDPSRGRDLYPSPKLSSAAKGLGYGENGSMPLSTKTMYWSSQSETCTESVAPASEKRPANGCRLFGIE
LRDCPTIDESSSVAMPSAVMEDLPAPSLNIDSDRNSEPSNPIPSVSCEPEKSSLRSTHESQSKQIRSCTKVHMQGKAVG
RAVDLTRLDSYEDLLKKLEEMFEIEGELRGSTKKWQVVYTDNEDDMMMVGDDPWHEFCSMVRKIYVYTAEEAKKLSPKI
KLPVDDVKPVSDAGIVSNEEKA

>StARF2b

MATSESCRNATGAGKVDAEKALYTELWRACAGPLVTVPCEDELVFYFPQGHIEQVEASTNQASDQQMPVYNLRSKILCR
VINVLLKAEPDTDEVYAQVTLLPEPNQDENVVSKEPMPSPPPRFHVHSFCKTLTASDTSTHGGFSVLRRHADECLPPLD
MSRQPPTQELVAKDLHANEWRFRHIFRGQPRRHLLQSGWSVFVSSKRLVAGDAFIFLRGENGELRVGVRRAMRQQGNAP
SSVISSHSMHLGVLATAWHAIQTKTLFTVYYKPRTSPAEFIVPYDQYMESLKNNYSIGMRFKMRFEGEEAPEQRFTGTI
VGIENADLKRWPESKWRCLKVRWDETSAIPRPDRVSPWKVEPALSPPALDPLPIPRQKRPRSNVLPSSPDSSVLTREGS
SKVTVDPSQASGFSRVLQGQEISTLRGNFVENNESDSSEKPPVWQPLLDDEKADVHSASRKCISDKRFPLARPESSLTD
LLSGFGGQSSSSHGFHSPTRGQTAPASWVKRQALDKETDFSLLGKQWSLVSSGLSLNLMDSGLKGADTLYQMRGTSRLN
GFNEYPTLPGHRTDNQQGSWLMSPSVLPYIQMSTRSGEIMPKPMASPQPEAMKPKEGNCKLFGIPLVSKCATIDPVMLR
KNSPIDSTSNMHFGIHPHQFPITESDQRSEQSKGSKLPDDGITVHDQEEQFQTSHPGTRDREGKVLVNSTRSCTKVHKQ
GTALGRSVDLAKFNNYEELIAELDHIFDFNGELKARNKNWLVVYTDDEGDMMLVGDDPWQEFCGMVRKIFIYTKDEVQR
MNPGTLNSKGEDISSVAEGSDGKEVKNLQLHSDSSPEDS

>StARF2a

MAASEVSIQGYSEPSDGSRPVSDTVRSSSGVGRVDADTALYTELWRSCAGPLVTVPREGELVYYFPQGHIEQVEASTNQ
VADQQMPSYNLPSKILCRVVNVLLKAEPDTDEVYAQVTLMPEPNQDENTVKKEPMRPPPPRFHVHSFCKTLTASDTSTH
GGFSVLRRHADECLPQLDMSRQPPTQELVAKDLHGNEWRFRHIFRGQPRRHLLQSGWSVFVSSKRLVAGDAFIFLRGEN
GDLRVGVRRAMRQQGNAPSSVISSHSMHLGVLATAWHAIQTKTMFTVYYKPRTSPTEFIVPYDHYMESVKNNYSIGMRF
KMRFEGEEAPEQRFTGTIVGIEDADPQRWLESKWRCLKVRWDENSSIPRPDRVSPWKIEPALSPPALNAPPVARPKRPR
SSILPSSPDSSVLTREGSSRVTADHSQASGFPRVLQGQELSTFRGGFAESNETDLSEKPMIWQPSVNDEKNDIHSASKR
YLPDKWLPLGRPESSLTDLLSGFGVPNNSSHGFCPSADQAAFGASLVKQQTQDQEKDFSLLGKPWSLLSSGLSLNLMDS
GSKAPGIGGDTPYQMRGDARYSGYGEFSVLPGHRVANQQGSWIMPQPVSPYMHSREMMHKPTVVKQPEAVKPKEGNCKL
FGIPLTSNVCTDPVMMRKSSLIDPASDMNIGIHPHQSLATDSDQRSEQSKGSKVDDGIAANDHDKQFHTFHLSARDRDG
KGHSSSTRSCTKVHKQGTALGRSVDLAKFNNYDELIAELDQLFDFNGELKARSKSWLVVYTDDEGDMMLVGDDPWQEFC
GMVRKIFIYTKEEVQRMNPGTLNSKGEDTSSVAEGSDAKEVKNLQLPSESGQAES

6. *Arabidopsis thaliana* ARFs

>AtARF19

MKAPSNGFLPSSNEGEKKPINSQLWHACAGPLVSLPPVGSLVVYFPQGHSEQVAASMQKQTDFIPNYPNLPSKLICLLH
SVTLHADTETDEVYAQMTLQPVNKYDREALLASDMGLKLNRQPTEFFCKTLTASDTSTHGGFSVPRRAAEKIFPPLDFS
MQPPAQEIVAKDLHDTTWTFRHIYRGQPKRHLLTTGWSVFVSTKRLFAGDSVLFVRDEKSQLMLGIRRANRQTPTLSSS
VISSDSMHIGILAAAAHANANSSPFTIFFNPRASPSEFVVPLAKYNKALYAQVSLGMRFRMMFETEDCGVRRYMGTVTG
ISDLDPVRWKGSQWRNLQVGWDESTAGDRPSRVSIWEIEPVITPFYICPPPFFRPKYPRQPGMPDDELDMENAFKRAMP
WMGEDFGMKDAQSSMFPGLSLVQWMSMQQNNPLSGSATPQLPSALSSFNLPNNFASNDPSKLLNFQSPNLSSANSQFNK
PNTVNHISQQMQAQPAMVKSQQQQQQQQQQHQHQQQQLQQQQQLQMSQQQVQQQGIYNNGTIAVANQVSCQSPNQPTGF
SQSQLQQQSMLPTGAKMTHQNINSMGNKGLSQMTSFAQEMQFQQQLEMHNSSQLLRNQQEQSSLHSLQQNLSQNPQQLQ
MQQQSSKPSPSQQLQLQLLQKLQQQQQQQSIPPVSSSLQPQLSALQQTQSHQLQQLLSSQNQQPLAHGNNSFPASTFMQ
PPQIQVSPQQQGQMSNKNLVAAGRSHSGHTDGEAPSCSTSPSANNTGHDNVSPTNFLSRNQQQGQAASVSASDSVFERA
SNPVQELYTKTESRISQGMMNMKSAGEHFRFKSAVTDQIDVSTAGTTYCPDVVGPVQQQQTFPLPSFGFDGDCQSHHPR
NNLAFPGNLEAVTSDPLYSQKDFQNLVPNYGNTPRDIETELSSAAISSQSFGIPSIPFKPGCSNEVGGINDSGIMNGGG
LWPNQTQRMRTYTKVQKRGSVGRSIDVTRYSGYDELRHDLARMFGIEGQLEDPLTSDWKLVYTDHENDILLVGDDPWEE
FVNCVQNIKILSSVEVQQMSLDGDLAAIPTTNQACSETDSGNAWKVHYEDTSAAASFNR

>AtARF5

MMASLSCVEDKMKTSCLVNGGGTITTTTSQSTLLEEMKLLKDQSGTRKPVINSELWHACAGPLVCLPQVGSLVYYFSQG
HSEQVAVSTRRSATTQVPNYPNLPSQLMCQVHNVTLHADKDSDEIYAQMSLQPVHSERDVFPVPDFGMLRGSKHPTEFF
CKTLTASDTSTHGGFSVPRRAAEKLFPPLDYSAQPPTQELVVRDLHENTWTFRHIYRGQPKRHLLTTGWSLFVGSKRLR
AGDSVLFIRDEKSQLMVGVRRANRQQTALPSSVLSADSMHIGVLAAAAHATANRTPFLIFYNPRACPAEFVIPLAKYRK
AICGSQLSVGMRFGMMFETEDSGKRRYMGTIVGISDLDPLRWPGSKWRNLQVEWDEPGCNDKPTRVSPWDIETPESLFI
FPSLTSGLKRQLHPSYFAGETEWGSLIKRPLIRVPDSANGIMPYASFPSMASEQLMKMMMRPHNNQNVPSFMSEMQQNI
VMGNGGLLGDMKMQQPLMMNQKSEMVQPQNKLTVNPSASNTSGQEQNLSQSMSAPAKPENSTLSGCSSGRVQHGLEQSM
EQASQVTTSTVCNEEKVNQLLQKPGASSPVQADQCLDITHQIYQPQSDPINGFSFLETDELTSQVSSFQSLAGSYKQPF
ILSSQDSSAVVLPDSTNSPLFHDVWDTQLNGLKFDQFSPLMQQDLYASQNICMSNSTTSNILDPPLSNTVLDDFCAIKD
TDFQNHPSGCLVGNNNTSFAQDVQSQITSASFADSQAFSRQDFPDNSGGTGTSSSNVDFDDCSLRQNSKGSSWQKIATP
RVRTYTKVQKTGSVGRSIDVTSFKDYEELKSAIECMFGLEGLLTHPQSSGWKLVYVDYESDVLLVGDDPWEEFVGCVRC
IRILSPTEVQQMSEEGMKLLNSAGINDLKTSVS

>AtARF6

MRLSSAGFNPQPHEGEKRVLNSELWHACAGPLVSLPPVGSRVVYFPQGHSEQVAASTNKEVDAHIPNYPSLHPQLICQL
HNVTMHADVETDEVYAQMTLQPLNAQEQKDPYLPAELGVPSRQPTNYFCKTLTASDTSTHGGFSVPRRAAEKVFPPLDY
SQQPPAQELMARDLHDNEWKFRHIFRGQPKRHLLTTGWSVFVSAKRLVAGDSVLFIWNDKNQLLLGIRRANRPQTVMPS
SVLSSDSMHLGLLAAAAHAAATNSRFTIFYNPRASPSEFVIPLAKYVKAVYHTRVSVGMRFRMLFETEESSVRRYMGTI
TGICDLDPTRWANSHWRSVKVGWDESTAGERQPRVSLWEIEPLTTFPMYPSPFPLRLKRPWPPGLPSFHGLKEDDMGMS
MSSPLMWDRGLQSLNFQGMGVNPWMQPRLDTSGLLGMQNDVYQAMAAAALQDMRGIDPAKAAASLLQFQNSPGFSMQSP
SLVQPQMLQQQLSQQQQQLSQQQQQQQQLSQQQQQQLSQQQQQQLSQQQQQQLSQQQQQQAYLGVPETHQPQSQAQSQS
NNHLSQQQQQVVDNHNPSASSAAVVSAMSQFGSASQPNTSPLQSMTSLCHQQSFSDTNGGNNPISPLHTLLSNFSQDES
SQLLHLTRTNSAMTSSGWPSKRPAVDSSFQHSGAGNNNTQSVLEQLGQSHTSNVPPNAVSLPPFPGGRECSIEQEGSAS
DPHSHLLFGVNIDSSSLLMPNGMSNLRSIGIEGGDSTTLPFTSSNFNNDFSGNLAMTTPSSCIDESGFLQSSENLGSEN
PQSNTFVKVYKSGSFGRSLDISKFSSYHELRSELARMFGLEGQLEDPVRSGWQLVFVDRENDVLLLGDDPWPEFVSSVW
CIKILSPQEVQQMGKRGLELLNSAPSSNNVDKLPSNGNCDDFGNRSDPRNLGNGIASVGGSFNY

>AtARF13

MENNGEMNAQPELSVDITKTYMYEKLWNICAGPLCVLPKPGEKVYYFPQGHIELIENSTRDELDHIRPIFDLPSKLRCR
VVAIDRKVDKNTDEVYAQISLMPDTTEVMTHNTTMDTRRPIVYFFSKILTASDVSLSGGLIIPKQYAIECFPPLDMSQP
ISTQNLVAKDLYGQEWSFKHVFRGTPQRHMFTSGGGWSVFATTKRLIVGDIFVLLRGENGELRFGIRRAKHQQGHIPSS
VISANCMQHGVIASVVNAFKTKCMFNVVYKPSSSQFVISYDKFVDAMNNNYIVGSRFRMQFEGKDFSEKRYDGTIIGVN
DMSPHWKDSEWRSLKVQWDELSPFLRPNQVSPWDIEHLIPSSDISQSSLKKKKHWLQLNEIGATLSNLWTCQEIGQRSM
NSPISVPEFSYPNAIEDSKFLSGLLLNHSLLAIPNENYNSDQMIQPRKEDITTEATTSCLLFGVDLTKVSKSKDSICPI
ESCKKSEISKLKNQKATTSCLKIKSLTKPNL

>AtARF12

MESGNVVNAQPELSGIIDGSKSYVYEQLWKLCAGPLCDIPKLGEKVYYFPQGHIELVETSTREELNELQPICDLPSKLQ
CRVIAIHLKVENNSDETYAEITLMPDTTQVVIPTQNENQFRPLVNSFTKVLTASDTSAHGGFFVPKKHAIECLPSLDMS
QPLPAQELLAIDLHGNQWRFNHNYRGTPQRHLLTTGWNAFTTSKKLVAGDVIVFVRGETGELRVGIRRARHQQGNIPSS
IVSIDCMRHGVVASAKHAFDNQCMFTVVYKPRSSKFIVSYDKFLDAVNNKFNVGSRFTMRLEGDDFSERRCFGTIIGVS
DFSPHWKCSEWRSLEVQWDEFTSFPGPKKVSPWDIEHLMPAINVPRSFLLKNKRLREVNEIGSSSSHLLPPILTQGQEN
EQLSVASPMNISLRYRDATEDAMNPSKLLMSYPVQPMPKLNYNNQMVTEMEENITTKTGTNFRLFGVTLDTPPVIKDPI
EEIGSEISKLTEGKKFGLSQTLRSPTEIQNKQFSSSRTCTKVQMQGVTIGRAVDLSVLNGYDQLILELEKLFDIKGQLQ
TRNQWEIAFTDSDEDKMLVGDDPWPEFCNMVKKIFIQKRR

>AtARF22

MESGNIVNAQPELSGIIDGSKSYMYEQLWKLCAGPLCDIPKLGEKIYYFPQGNIELVEASTREELNELKPICDLPSKLQ
CRVIAIQLKVENNSDETYAEITLMPDTTQVVIPTQNENQFRPLVNSFTKVLTASDTSGGFFVPKKHAIECLPPLDMSQP
LPTQELLATDLHGNQWRFNHNYRGTPQRHLLTTGWNAFTTSKKLVAGDVIVFVRGETGELRVGIRRAGHQQGNIPSSII
SIESMRHGVIASAKHAFDNQCMFIVVYKPRSSQFIVSYDKFLDAVNNKFNVGSRFTMRFEGDDFSERRYFGTIIGVSDF
SPHWKCSEWRNLEVQWDEFASFSRPNKVSPWEIEHLMPALNVPRPSLLKNKRLREVNEIGSSSSHLLPPILTQGQEIGQ
LSVASPMNISLTYRDTTEDVMNPSRLLMSYPVQPMPKLNYNNQMVTQIEENITTKTGTNFRLFGVSLVTPSVIKDPIEE
IGSEISKLTEGKKFGQSQTLRSPTEIQSKQFSSTRTCTKVQMQGVTIERAVDLSVLNGYDQLILELEELFDLKGQLQTR
NQWEIAFTDSDDDKMLVGDDPWPEFCNMVKKILIFKRGGQKLEVQ

>AtARF21

MESGNIVNAQPKLSGIIDGSKSYMYEQLWKLCAGPLCDIPKLGENVYYFPQGNIELVQASTREELNELQPICDLPSKLQ
CRVIAIHLKVENNSDEIYAEITLMPDTTQVVIPTQSENRFRPLVNSFTKVLTASDTSAYGGFSVPKKHAIECLPPLDMS
QPLPAQEILAIDLHDNQWRFRHNYRGTPQRHSLTTGWNEFITSKKLVKGDVIVFVRGETGELRVGIRRARHQQGNIPSS
IVSIDCMRHGVIASAKHAFDNQCIFIVVYKPRSSQFIVSYDKFLDAVNNKFNVGSRFTMRFEGDDFSERRYFGTIIGVS
DFSPHWKCSEWRSLEVQWDEFASFSRPNKVSPWEIEHLVPALNVPRSSLLKNKRLREVNEFGSSSSHLLPPILTQGQEI
GQLSVASPMNISLRYRDTTEAAMNPSRLLMSYPVQPMPKLNYNNQMVTQIEENITTKAGTNFRLFGVTLDTPPMIKDPI
KQIGSDISKLTERKKFGQSQTLRSPIEIQSKQFSSSRTCTKVQMQGVTIGRAVDLSVLNGYDQLILELEKLFDIKGQLQ
TRNQWKIAFTDSDGYEMLVGDDPWPEFCKMVKKILIYSKEEVKNLKSSKSLSS

>AtARF20

METGNVVNAQPELSGIIDGSKSYMYEQLWKLCAGPLCDIPKLGENVYYFPQGNIELVDASTREELNELQPICDLPSKLQ
CRVIAIHLKVENNSDETYAEITLMPDTTQVVIPTQSENQFRPLVNSFTKVLTASDTSAYGGFFVPKKHAIECLPPLPLP
AQELLAKDLHGNQWRFRHSYRGTPQRHSLTTGWNEFTTSKKLVKGDVIVFVRGETGELRVGIRRARHQQGNIPSSIVSI
DCMRHGVIASAKHALDNQCIFIVVYKPSIRSSQFIVSYDKFLDAMNNKFIVGSRFTMRFEGDDFSERRYFGTIIGVNDF
SPHWKCSEWRSLEVQWDEFASFSRPNKVSPWEIEHLMSALNVPRSSLLKNKRLREVNEFGQEIGQLSVASPMNTSLRYR
DTTEDAMNPSRLLMSYPVQPMPKLNYNNQMVTQIEENITTKAVTNFRLFGVSLAIPLVIKDPIEEIGSDISKLTEGKKF
GQSQTLRSPIEIQSKQFGSTRTCTKVQMQGVTIGRAVDLSVLNGYDQLILELEKLFDLKGQLQTRNQWKIAFTDSDGYE
MLVGDDPWPEFCKMVKKILIYSKEEVKNLKSSKSLSS

>AtARF15

METGNVVNAQPELSGIIDRSKSYMYEQLWKLCAGPLCDIPKLGEKVYYFPQGNIELVEASTREELNELQPICDLPSKLQ
CRVIAIHLKVENNSDETYAKITLMPDTTVSENLQVVIPTQNENQFRPLVNSFTKVLTASDISANGVFSVPKKHAIECLP
PLDMSQPLPAQELLAIDLHGNQWSFRHSYRGTPQRHLLTTGWNEFTTSKKLVKGDVIVFVRGETGELRVGIRRARHQQG
NIPSSIVSIDCMRHGVIASAKHAFDNQCMFIVVYKPRSSQFIVSYDKFLDAVNNKFNVGSRFTMRFEGDDLSERRYFGT
IIGVSNFSPHWKCSDWRSLEVQWDEFASFLRPNKVSPWEIEHLMPALNVPRSSFLKNKRLREVNEFGSSSSHLLPPILT
QGQEIGQLSVASPMNISLLYRETTEDAMNPSRLLMSYPVQPMPKRNYNNQMVTQIEENITTKAGTNFRLFGVSLATPPV
IKDPIEQIGSDISKLTEGKKFGQSQTLRSPTKIQSKQFSSTRTCTKVQMQGVTIGRAVDLSVLNGYDQLILELEKLFDL
KGQLQTRNQWKIIFTGSDEDEMLVGDDPWPEFCNMVKRIYIQKRR

>AtARF14

MESGNVVNTQPELSGIIDGSKSYMYEQLWKLCAGPLCDIPKLGEKVYYFPQGHIELVEASTREELNELQPICDFPSKLQ
CRVIAIQLKVENNSDETYAEITLMPDTTQVVIPTQNQNQFRPLVNSFTKVLTASDTSVHGGFSVPKKHAIECLPPLDMS
QPLPTQEILAIDLHGNQWRFRHIYRGTAQRHLLTIGWNAFTTSKKLVEGDVIVFVRGETGELRVGIRRAGHQQGNIPSS
IVSIESMRHGIIASAKHAFDNQCMFIVVYKPRSSQFIVSYDKFLDVVNNKFNVGSRFTMRFEGDDFSERRSFGTIIGVS
DFSPHWKCSEWRSLEVQWDEFASFPRPNQVSPWDIEHLTPWSNVSRSSFLKNKRSREVNEIGSSSSHLLPPTLTQGQEI
GQQSMATPMNISLRYRDITEDAMTPSRLLMSYPVQPMAKLNYNNVVTPIEENITTNAVASFRLFGVSLATPSVIKDPVE
QIGLEISRLTQEKKFGQSQILRSPTEIQSKQFSSTRTCTKVQMQGVTIGRAVDLSVLNGYDQLILELEKLFDLKGQLQA
RNQWEIAFTNNEEDKMLVGEDPWPEFCNMVKKIFIYSKEEVKNLKSRKSLSS

>AtARF1

MAASNHSSGKPGGVLSDALCRELWHACAGPLVTLPREGERVYYFPEGHMEQLEASMHQGLEQQMPSFNLPSKILCKVIN
IQRRAEPETDEVYAQITLLPELDQSEPTSPDAPVQEPEKCTVHSFCKTLTASDTSTHGGFSVLRRHADDCLPPLDMSQQ
PPWQELVATDLHNSEWHFRHIFRGQPRRHLLTTGWSVFVSSKKLVAGDAFIFLRGENEELRVGVRRHMRQQTNIPSSVI
SSHSMHIGVLATAAHAITTGTIFSVFYKPRTSRSEFIVSVNRYLEAKTQKLSVGMRFKMRFEGEEAPEKRFSGTIVGVQ
ENKSSVWHDSEWRSLKVQWDEPSSVFRPERVSPWELEPLVANSTPSSQPQPPQRNKRPRPPGLPSPATGPSGPVTPDGV
WKSPADTPSSVPLFSPPAKAATFGHGGNKSFGVSIGSAFWPTNADSAAESFASAFNNESTEKKQTNGNVCRLFGFELVE
NVNVDECFSAASVSGAVAVDQPVPSNEFDSGQQSEPLNINQSDIPSGSGDPEKSSLRSPQESQSRQIRSCTKVHMQGSA
VGRAIDLTRSECYEDLFKKLEEMFDIKGELLESTKKWQVVYTDDEDDMMMVGDDPWNEFCGMVRKIFIYTPEEVKKLSP
KNKLAVNARMQLKADAEENGNTEGRSSSMAGSR

>AtARF17

MSPPSATAGDINHREVDPTIWRACAGASVQIPVLHSRVYYFPQGHVEHCCPLLSTLPSSTSPVPCIITSIQLLADPVTD
EVFAHLILQPMTQQQFTPTNYSRFGRFDGDVDDNNKVTTFAKILTPSDANNGGGFSVPRFCADSVFPLLNFQIDPPVQK
LYVTDIHGAVWDFRHIYRGTPRRHLLTTGWSKFVNSKKLIAGDSVVFMRKSADEMFIGVRRTPISSSDGGSSYYGGDEY
NGYYSQSSVAKEDDGSPKKTFRRSGNGKLTAEAVTDAINRASQGLPFEVVFYPAAGWSEFVVRAEDVESSMSMYWTPGT
RVKMAMETEDSSRITWFQGIVSSTYQETGPWRGSPWKQLQITWDEPEILQNVKRVNPWQVEIAAHATQLHTPFPPAKRL
KYPQPGGGFLSGDDGEILYPQSGLSSAAAPDPSPSMFSYSTFPAGMQGARQYDFGSFNPTGFIGGNPPQLFTNNFLSPL
PDLGKVSTEMMNFGSPPSDNLSPNSNTTNLSSGNDLVGNRGPLSKKVNSIQLFGKIITVEEHSESGPAESGLCEEDGSK
ESSDNETQLSLSHAPPSVPKHSNSNAGSSSQG

>AtARF10

MEQEKSLDPQLWHACAGSMVQIPSLNSTVFYFAQGHTEHAHAPPDFHAPRVPPLILCRVVSVKFLADAETDEVFAKITL
LPLPGNDLDLENDAVLGLTPPSSDGNGNGKEKPASFAKTLTQSDANNGGGFSVPRYCAETIFPRLDYSAEPPVQTVIAK
DIHGETWKFRHIYRGTPRRHLLTTGWSTFVNQKKLIAGDSIVFLRSESGDLCVGIRRAKRGGLGSNAGSDNPYPGFSGF
LRDDESTTTTSKLMMMKRNGNNDGNAAATGRVRVEAVAEAVARAACGQAFEVVYYPRASTPEFCVKAADVRSAMRIRWC
SGMRFKMAFETEDSSRISWFMGTVSAVQVADPIRWPNSPWRLLQVAWDEPDLLQNVKRVSPWLVELVSNMPTIHLSPFS
PRKKIRIPQPFEFPFHGTKFPIFSPGFANNGGGESMCYLSNDNNNAPAGIQGARQAQQLFGSPSPSLLSDLNLSSYTGN
NKLHSPAMFLSSFNPRHHHYQARDSENSNNISCSLTMGNPAMVQDKKKSVGSVKTHQFVLFGQPILTEQQVMNRKRFLE
EEAEAEEEKGLVARGLTWNYSLQGLETGHCKVFMESEDVGRTLDLSVIGSYQELYRKLAEMFHIEERSDLLTHVVYRDA
NGVIKRIGDEPFSDFMKATKRLTIKMDIGGDNVRKTWITGIRTGENGIDASTKTGPLSIFA

>AtARF3

MGGLIDLNVMETEEDETQTQTPSSASGSVSPTSSSSASVSVVSSNSAGGGVCLELWHACAGPLISLPKRGSLVLYFPQG
HLEQAPDFSAAIYGLPPHVFCRILDVKLHAETTTDEVYAQVSLLPESEDIERKVREGIIDVDGGEEDYEVLKRSNTPHM
FCKTLTASDTSTHGGFSVPRRAAEDCFPPLDYSQPRPSQELLARDLHGLEWRFRHIYRGQPRRHLLTTGWSAFVNKKKL
VSGDAVLFLRGDDGKLRLGVRRASQIEGTAALSAQYNQNMNHNNFSEVAHAISTHSVFSISYNPKASWSNFIIPAPKFL
KVVDYPFCIGMRFKARVESEDASERRSPGIISGISDLDPIRWPGSKWRCLLVRWDDIVANGHQQRVSPWEIEPSGSISN
SGSFVTTGPKRSRIGFSSGKPDIPVSEGIRATDFEESLRFQRVLQGQEIFPGFINTCSDGGAGARRGRFKGTEFGDSYG
FHKVLQGQETVPAYSITDHRQQHGLSQRNIWCGPFQNFSTRILPPSVSSSPSSVLLTNSNSPNGRLEDHHGGSGRCRLF
GFPLTDETTAVASATAVPCVEGNSMKGASAVQSNHHHSQGRDIYAMRDMLLDIAL

>AtARF11

MANVEADFRTSGSNDDELYTELWKACAGPLVEVPRYGERVFYFPQGHMEQLVASTNQGVVDQEIPVFNLPPKILCRVLS
VTLKAEHETDEVYAQITLQPEEDQSEPTSLDPPLVEPAKPTVDSFVKILTASDTSTHGGFSVLRKHATECLPSLDMTQP
TPTQELVARDLHGYEWRFKHIFRGQPRRHLLTTGWSTFVTSKRLVAGDAFVFLRGETGDLRVGVRRLAKQQSTMPASVI
SSQSMRLGVLATASHAVTTTTIFVVFYKPRISQFIISVNKYMMAMKNGFSLGMRYRMRFEGEESPERIFTGTIIGSGDL
SSQWPASKWRSLQIQWDEPSSIQRPNKVSPWEIEPFSPSALTPTPTQQQSKSKRSRPISEITGSPVASSFLSSFSQSHE
SNPSVKLLFQDPATERNSNKSVFSSGLQCKITEAPVTSSCRLFGFDLTSKPASATIPHDKQLISVDSNISDSTTKCQDP
NSSNSPKEQKQQTSTRSRIKVQMQGTAVGRAVDLTLLRSYDELIKELEKMFEIEGELSPKDKWAIVFTDDEGDRMLVGD
DPWNEFCKMAKKLFIYPSDEVKKMRSKSLLGDKGTIVNLESDQRTVHV

>AtARF18

MASVEGDDDFGSSSSRSYQDQLYTELWKVCAGPLVEVPRAQERVFYFPQGHMEQLVASTNQGINSEEIPVFDLPPKILC
RVLDVTLKAEHETDEVYAQITLQPEEDQSEPTSLDPPIVGPTKQEFHSFVKILTASDTSTHGGFSVLRKHATECLPSLD
MTQATPTQELVTRDLHGFEWRFKHIFRGQPRRHLLTTGWSTFVSSKRLVAGDAFVFLRGENGDLRVGVRRLARHQSTMP
TSVISSQSMHLGVLATASHAVRTTTIFVVFYKPRISQFIVGVNKYMEAIKHGFSLGTRFRMRFEGEESPERIFTGTIVG
SGDLSSQWPASKWRSLQVQWDEPTTVQRPDKVSPWEIEPFLATSPISTPAQQPQSKCKRSRPIEPSVKTPAPPSFLYSL
PQSQDSINASLKLFQDPSLERISGGYSSNNSFKPETPPPPTNCSYRLFGFDLTSNSPAPIPQDKQPMDTCGAAKCQEPI
TPTSMSEQKKQQTSRSRTKVQMQGIAVGRAVDLTLLKSYDELIDELEEMFEIQGQLLARDKWIVVFTDDEGDMMLAGDD
PWNEFCKMAKKIFIYSSDEVKKMTTKLKISSSLENEEYGNESFENRSRG

>AtARF9

MANRGGEYLYDELWKLCAGPLVDVPQAQERVYYFPQGHMEQLEASTQQVDLNTMKPLFVLPPKILCNVMNVSLQAEKDT
DEVYAQITLIPVGTEVDEPMSPDPSPPELQRPKVHSFSKVLTASDTSTHGGFSVLRKHATECLPPLDMTQQTPTQELVA
EDVHGYQWKFKHIFRGQPRRHLLTTGWSTFVTSKRLVAGDTFVFLRGENGELRVGVRRANLQQSSMPSSVISSHSMHLG
VLATARHATQTKTMFIVYYKPRTSQFIISLNKYLEAMSNKFSVGMRFKMRFEGEDSPERRYSGTVIGVKDCSPHWKDSK
WRCLEVHWDEPASISRPNKVSPWEIEPFVNSENVPKSVMLKNKRPRQVSEVSALDVGITASNLWSSVLTQPHEFAQSCI
TSQWSSPQQCHRDANEDAKKSDWLNNSYSVSNVAKDSTLNDQMVSPVEQKKPETTANYRLFGIDLMSSSLAVPEEKTAP
MRPINISKPTMDSHSDPKSEISKVSEEKKQEPAEGSPKEVQSKQSSSTRSRTKVQMQGVPVGRAVDLNALKGYNELIDD
IEKLFDIKGELRSRNQWEIVFTDDEGDMMLVGDDPWPEFCNMVKRIFIWSKEEVKKMTPGNQLRMLLREVETTLTTTSK
TDNHSN

>AtARF16

MINVMNPMKGGTEKGLDPQLWHACAGGMVRMPPMNSKVFYFPQGHAENAYDCVDFGNLPIPPMVLCRVLAIKYMADAES
DEVFAKLRLIPLKDDEYVDHEYGDGEDSNGFESNSEKTPSFAKTLTQSDANNGGGFSVPRYCAETIFPRLDYNAEPPVQ
TILAKDVHGDVWKFRHIYRGTPRRHLLTTGWSNFVNQKKLVAGDSIVFMRAENGDLCVGIRRAKRGGIGNGPEYSAGWN
PIGGSCGYSSLLREDESNSLRRSNCSLADRKGKVTAESVIEAATLAISGRPFEVVYYPRASTSEFCVKALDARAAMRIP
WCSGMRFKMAFETEDSSRISWFMGTVSAVNVSDPIRWPNSPWRLLQVAWDEPDLLQNVKRVNPWLVELVSNVHPIPLTS
FSPPRKKMRLPQHPDYNNLINSIPVPSFPSNPLIRSSPLSSVLDNVPVGLQGARHNAHQYYGLSSSDLHHYYLNRPPPP
PPPSSLQLSPSLGLRNIDTKNEKGFCFLTMGTTPCNDTKSKKSHIVLFGKLILPEEQLSEKGSTDTANIEKTQISSGGS
NQNGVAGREFSSSDEGSPCSKKVHDASGLETGHCKVFMESDDVGRTLDLSVLGSYEELSRKLSDMFGIKKSEMLSSVLY
RDASGAIKYAGNEPFSEFLKTARRLTILTEQGSESVVV

>AtARF7

MKAPSSNGVSPNPVEGERRNINSELWHACAGPLISLPPAGSLVVYFPQGHSEQVAASMQKQTDFIPSYPNLPSKLICML
HNVTLNADPETDEVYAQMTLQPVNKYDRDALLASDMGLKLNRQPNEFFCKTLTASDTSTHGGFSVPRRAAEKIFPALDF
SMQPPCQELVAKDIHDNTWTFRHIYRGQPKRHLLTTGWSVFVSTKRLFAGDSVLFIRDGKAQLLLGIRRANRQQPALSS
SVISSDSMHIGVLAAAAHANANNSPFTIFYNPRWAAPAEFVVPLAKYTKAMYAQVSLGMRFRMIFETEECGVRRYMGTV
TGISDLDPVRWKNSQWRNLQIGWDESAAGDRPSRVSVWDIEPVLTPFYICPPPFFRPRFSGQPGMPDDETDMESALKRA
MPWLDNSLEMKDPSSTIFPGLSLVQWMNMQQQNGQLPSAAAQPGFFPSMLSPTAALHNNLGGTDDPSKLLSFQTPHGGI
SSSNLQFNKQNQQAPMSQLPQPPTTLSQQQQLQQLLHSSLNHQQQQSQSQQQQQQQQLLQQQQQLQSQQHSNNNQSQSQ
QQQQLLQQQQQQQLQQQHQQPLQQQTQQQQLRTQPLQSHSHPQPQQLQQHKLQQLQVPQNQLYNGQQAAQQHQSQQAST
HHLQPQLVSGSMASSVITPPSSSLNQSFQQQQQQSKQLQQAHHHLGASTSQSSVIETSKSSSNLMSAPPQETQFSRQVE
QQQPPGLNGQNQQTLLQQKAHQAQAQQIFQQSLLEQPHIQFQLLQRLQQQQQQQFLSPQSQLPHHQLQSQQLQQLPTLS

QGHQFPSSCTNNGLSTLQPPQMLVSRPQEKQNPPVGGGVKAYSGITDGGDAPSSSTSPSTNNCQISSSGFLNRSQSGPA
ILIPDAAIDMSGNLVQDLYSKSDMRLKQELVGQQKSKASLTDHQLEASASGTSYGLDGGENNRQQNFLAPTFGLDGDSR
NSLLGGANVDNGFVPDTLLSRGYDSQKDLQNMLSNYGGVTNDIGTEMSTSAVRTQSFGVPNVPAISNDLAVNDAGVLGG
GLWPAQTQRMRTYTKVQKRGSVGRSIDVNRYRGYDELRHDLARMFGIEGQLEDPQTSDWKLVYVDHENDILLVGDDPWE
EFVNCVQSIKILSSAEVQQMSLDGNFAGVPVTNQACSGGDSGNAWRGHYDDNSATSFNR

>AtARF8

MKLSTSGLGQQGHEGEKCLNSELWHACAGPLVSLPSSGSRVVYFPQGHSEQVAATTNKEVDGHIPNYPSLPPQLICQLH
NVTMHADVETDEVYAQMTLQPLTPEEQKETFVPIELGIPSKQPSNYFCKTLTASDTSTHGGFSVPRRAAEKVFPPLDYT
LQPPAQELIARDLHDVEWKFRHIFRGQPKRHLLTTGWSVFVSAKRLVAGDSVIFIRNEKNQLFLGIRHATRPQTIVPSS
VLSSDSMHIGLLAAAAHASATNSCFTVFFHPRASQSEFVIQLSKYIKAVFHTRISVGMRFRMLFETEESSVRRYMGTIT
GISDLDSVRWPNSHWRSVKVGWDESTAGERQPRVSLWEIEPLTTFPMYPSLFPLRLKRPWHAGTSSLPDGRGDLGSGLT
WLRGGGGEQQGLLPLNYPSVGLFPWMQQRLDLSQMGTDNNQQYQAMLAAGLQNIGGGDPLRQQFVQLQEPHHQYLQQSA
SHNSDLMLQQQQQQQASRHLMHAQTQIMSENLPQQNMRQEVSNQPAGQQQQLQQPDQNAYLNAFKMQNGHLQQWQQQSE
MPSPSFMKSDFTDSSNKFATTASPASGDGNLLNFSITGQSVLPEQLTTEGWSPKASNTFSEPLSLPQAYPGKSLALEPG
NPQNPSLFGVDPDSGLFLPSTVPRFASSSGDAEASPMSLTDSGFQNSLYSCMQDTTHELLHGAGQINSSNQTKNFVKVY
KSGSVGRSLDISRFSSYHELREELGKMFAIEGLLEDPLRSGWQLVFVDKENDILLLGDDPWESFVNNVWYIKILSPEDV
HQMGDHGEGSGGLFPQNPTHL

>AtARF4

MEFDLNTEIAEVEEEENDDVGVGVGGGTRIDKGRLGISPSSSSSCSSGSSSSSSSTGSASSIYSELWHACAGPLTCLPK
KGNVVVYFPQGHLEQDAMVSYSSPLEIPKFDLNPQIVCRVVNVQLLANKDTDEVYTQVTLLPLQEFSMLNGEGKEVKEL
GGEEERNGSSSVKRTPHMFCKTLTASDTSTHGGFSVPRRAAEDCFAPLDYKQQRPSQELIAKDLHGVEWKFRHIYRGQP
RRHLLTTGWSIFVSQKNLVSGDAVLFLRDEGGELRLGIRRAARPRNGLPDSIIEKNSCSNILSLVANAVSTKSMFHVFY
SPRATHAEFVIPYEKYITSIRSPVCIGTRFRMRFEMDDSPERRCAGVVTGVCDLDPYRWPNSKWRCLLVRWDESFVSDH
QERVSPWEIDPSVSLPHLSIQSSPRPKRPWAGLLDTTPPGNPITKRGGFLDFEESVRPSKVLQGQENIGSASPSQGFDV
MNRRILDFAMQSHANPVLVSSRVKDRFGEFVDATGVNPACSGVMDLDRFPRVLQGQEICSLKSFPQFAGFSPAAAPNPF
AYQANKSSYYPLALHGIRSTHVPYQNPYNAGNQSSGPPSRAINFGEETRKFDAQNEGGLPNNVTADLPFKIDMMGKQKG
SELNMNASSGCKLFGFSLPVETPASKPQSSSKRICTKVHKQGSQVGRAIDLSRLNGYDDLLMELERLFNMEGLLRDPEK
GWRILYTDSENDMMVVGDDPWHDFCNVVWKIHLYTKEEVENANDDNKSCLEQAALMMEASKSSSVSQPDSSPTITRV

>AtARF2

MASSEVSMKGNRGGDNFSSSGFSDPKETRNVSVAGEGQKSNSTRSAAAERALDPEAALYRELWHACAGPLVTVPRQDDR
VFYFPQGHIEQVEASTNQAAEQQMPLYDLPSKLLCRVINVDLKAEADTDEVYAQITLLPEANQDENAIEKEAPLPPPPR
FQVHSFCKTLTASDTSTHGGFSVLRRHADECLPPLDMSRQPPTQELVAKDLHANEWRFRHIFRGQPRRHLLQSGWSVFV
SSKRLVAGDAFIFLRGENGELRVGVRRAMRQQGNVPSSVISSHSMHLGVLATAWHAISTGTMFTVYYKPRTSPSEFIVP
FDQYMESVKNNYSIGMRFKMRFEGEEAPEQRFTGTIVGIEESDPTRWPKSKWRSLKVRWDETSSIPRPDRVSPWKVEPA
LAPPALSPVPMPRPKRPRSNIAPSSPDSSMLTREGTTKANMDPLPASGLSRVLQGQEYSTLRTKHTESVECDAPENSVV
WQSSADDDKVDVVSGSRRYGSENWMSSARHEPTYTDLLSGFGTNIDPSHGQRIPFYDHSSSPSMPAKRILSDSEGKFDY
LANQWQMIHSGLSLKLHESPKVPAATDASLQGRCNVKYSEYPVLNGLSTENAGGNWPIRPRALNYYEEVVNAQAQAQAR
EQVTKQPFTIQEETAKSREGNCRLFGIPLTNNMNGTDSTMSQRNNLNDAAGLTQIASPKVQDLSDQSKGSKSTNDHREQ
GRPFQTNNPHPKDAQTKTNSSRSCTKVHKQGIALGRSVDLSKFQNYEELVAELDRLFEFNGELMAPKKDWLIVYTDEEN
DMMLVGDDPWQEFCCMVRKIFIYTKEEVRKMNPGTLSCRSEEEAVVGEGSDAKDAKSASNPSLSSAGNS

7. *Chlamydomonas reinhardti* ARF-like protein

>Cre13.g562400

MGDQVGGIVLPTTGDAGDGAGPPNFAALAGLLGGHPGAAAGFAPAPGAPGQPHPALSGLALSGMSHLAHMDPSTALLLS
GLTAQQPGGGDAAAAQMQFLHNPTAAYAAAAAAAAAAAAAAGQPAGVPVVDHGHHHAHHAHPVAGGADGGASGVKVEHD
GGGGGGVDGGEENAAVRAAAAAAAAAADPSDPEAVAAALAGRMAALGASVIFEKSLTASDVSGGGRVVVPKSIAEQYFP

RLEAPSGVTISAADLEGRAYTFKWRFWVNNSSRMYLLEGAGELHRNYGLEVGDVMVFAQKQDGSLVVAGRCANKADMVK
KQPVKRPNTAAAATAAVQAQPGPAAGRGGGRDARSGRTPARTSTPAAELAAPVTAQSPGAAAAAAAAAAAAAATAAGVAM
GGVRNGAGMGAGAGAAAAAADRGRKRKGPGSGAGQWAGGAVDGGRGGAARSGGRAGGGGGYPGYGYEGGAGVGAGGEEV
CLSVLDMEAPSDGVFRAVVLPANGTSGGHASTGAGTGGSSPGVGVGVALARNNRWTATLDVAGELYQAYFDCRDDAVEA
LTAAGASP
